# The brain of the female *Anopheles gambiae* mosquito at spatial and single-cell resolution

**DOI:** 10.1038/s41467-026-76839-z

**Published:** 2026-09-17

**Authors:** Irene Z. Rossi, Cécile Cassan, Johannes W. Bagnoli, Sümeyye Özhan, Adeline Valente, Quentin J. Delorme, Geert-Jan van Gemert, Chiara Andolina, Wouter Graumans, Richárd Bártfai, Avinash R. Shenoy, David Carrasco, Emmanuel Perisse, Teun Bousema, Sarah H. Merkling, Anna Cohuet, Arthur M. Talman

**Affiliations:** 1 MIVEGEC, Université de Montpellier, IRD, CNRS, Montpellier, France; 2 Institut Pasteur, Université Paris Cité, CNRS UMR2000, Insect Infection & Immunity Unit, Paris, France; 3 Sorbonne Université, Collège Doctoral, Paris, France; 4 Department of Medical Microbiology, Radboud University Medical Center, Nijmegen, the Netherlands; 5 Department of Molecular Biology, Radboud University, Nijmegen, the Netherlands; 6 Department of Infectious Disease, Imperial College, London, UK; 7 Institute of Functional Genomics, University of Montpellier, CNRS, Inserm, Montpellier Cedex 5, France

**Keywords:** Neuroscience, Microbiology, Systems biology

## Abstract

Blood feeding in *Anopheles* mosquitoes is at the root of all malaria infections in humans and is supported by a range of physiological and behavioral adaptations. Since behavior is orchestrated at the level of the central nervous system, we surveyed the cellular diversity and spatial organization of the female *Anopheles gambiae* brain and compared it to other insect species. We then describe transcriptional changes associated with mosquito life history events with single brain and single-cell RNA sequencing. We did not identify expansion of specific cell populations, but a modulation of neurotransmission in the mated mosquitoes, and a metabolic and immune response associated with glia, Kenyon cells and immune cells after bloodmeal, and detected signatures of aging in the mosquito brain. We did not find an effect of *Plasmodium falciparum* infection. Our atlas will empower the study of non-model insect neurobiology, neural basis of vector behavior, and mechanisms underlying malaria transmission.

## Abstract (web summary)

Mosquito behavior drives malaria transmission, yet the Anopheles brain remains largely uncharted. Here, authors show a single-cell and spatial atlas of the female An. gambiae brain and its transcriptional changes across key life-history events.

## Introduction

The nervous system is responsible for perception and integration of both endogenous and environmental cues, enabling decision-making and the generation of adaptive physiological and behavioral responses. The cellular diversity supporting this network is almost uncharted in insects, and most of the knowledge on the insect brain has been derived from *Drosophila melanogaster*^1,2^. In such a model system, cellular diversity can be interpreted in the light of decades of studies on cell lineages and developmental processes. However, highly diverse behaviors computed by specific cell networks are found among insect species^3,4^.The organization and diversity of cell types in the brain of non-model organisms and insect vectors has just started to be explored and contrasted to model organisms by means of cell type taxonomy methods and cross-species cellular annotation^5–7^. In these species, the progressive characterization of brain cell types is revealing the cellular basis of behavioral states. For instance, differences in Kenyon cell (KC) type composition and abundance in the mushroom bodies (MBs) are linked to behavioral states and social function in ants^5^. In bees, gene regulatory networks within defined KC populations underlie foraging behavior^8^, whereas in *Aedes* mosquitoes, blood feeding is accompanied by a massive transcriptional shift in glial cells^7^. These studies underscore the need for cell-resolved data for understanding how behavior emerges from neural transcriptional states.

In mosquitoes, blood feeding has evolved as an essential trait for nutrient acquisition, supporting egg development, but has also been exploited by pathogens as a means to gain access to new hosts. *Anopheles* females are the sole vectors of human *Plasmodium* parasites, and long-term ecological interactions have shaped their coevolution^9^*. Plasmodium falciparum* is also suspected to manipulate the host-seeking and biting behavior of its vector to enhance transmission success, albeit with conflicting evidence^10–12^. Yet, in *Anopheles,* very little information exists on the structure and composition of the brain, in spite of the central role of behavior in determining vectorial capacity, and, prior to this study, molecular characterization was limited to only two general neuronal markers and a single glial marker^13–15^. Here, we determine the cellular diversity of the female *An. gambiae* brain, spatially resolve the organization of the brain within mosquito heads, and establish transcriptional signatures of female mosquito aging and life history events associated with malaria parasite transmission. This approach allowed us to curate the first extensive annotation of the adult female *Anopheles* brain cell-type atlas and provide a comprehensive overview of the brain transcriptional processes associated with life-history traits.

## Results

### A cellular atlas of the female *An. gambiae* brain

We dissociated six pools of the brains of female *An. gambiae* mosquitoes (either virgin, mated or blood-fed), and analyzed them using single-cell RNA-seq (scRNA-seq) (10x Genomics) (Fig. S1A). After quality control, the *An. gambiae* brain atlas consisted of 48,806 high-quality cells (~20% of the expected number of cells in a single brain^14^), with an average of 5, 350 unique molecular identifiers (UMIs) and 1, 678 genes per cell (Fig. S2A–D). Using a conservative approach aimed at avoiding overclustering, we identified 45 cell clusters (hereafter Ag clusters). All cell clusters were present in all experimental replicates, indicating their consistent representation in the pools (Fig. S2E, F).

**Fig. 1:**
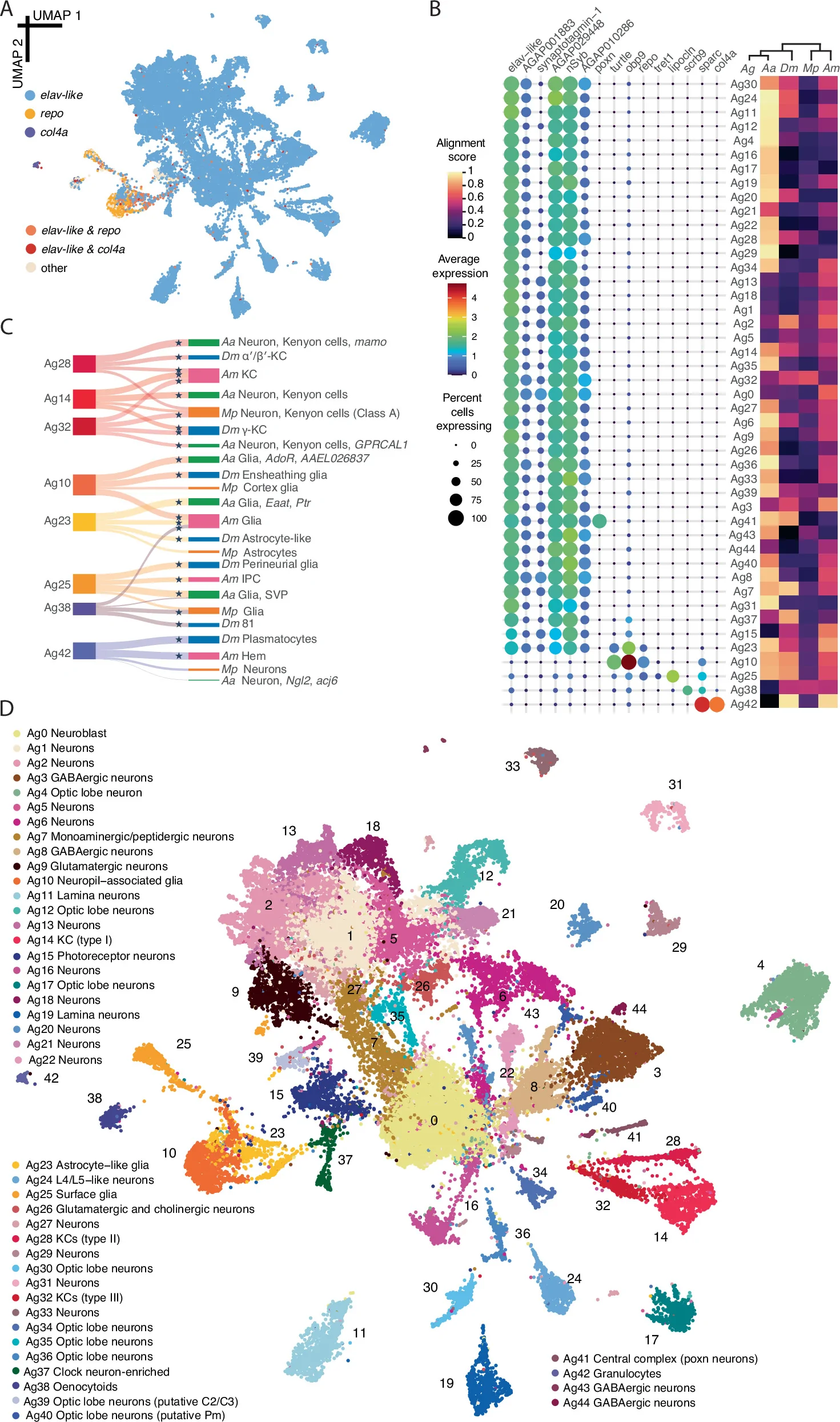
A cellular atlas of the female *An. gambiae* mosquito brain. **A** Uniform Manifold Approximation and Projection (UMAP) of 48,806 high-quality single-cell transcriptomes (from three experimental conditions with two experimental replicates each, for a total of six samples, each derived from ten brains) in the atlas, highlighting expression levels of *elav-like*, *repo* and *col4a*. **B** Notable marker gene expression in the 45 clusters of the atlas (cluster ID on top), alongside a heatmap showing the strength of alignment of Ag clusters to *Ae. aegypti* (Aa), *D. melanogaster* (Dm), *M. pharaonis* (Mp), and *A. mellifera* (Am) cell types. Neural markers: *elav-like* (*AGAP000965*)*, AGAP001883, synaptotagmin-1, AGAP029448, nSyb, AGAP010286*, *poxn*; glial markers: *turtle, obp9, repo, tret1, lipocln*; immune cell markers: *scrb9, sparc, col4a*. **C** Example of Ag clusters top alignments to cell types of the multispecies SAMap panel. Alignment values > 0.4 are displayed with a star. **D** UMAP of the brain atlas displaying consensus annotation of the Ag clusters.

We first mapped the expression of orthologs of general cellular markers of neurons (multiple genes, among which the most expressed was *elav-like*) and glia (*repo*), as these markers are widely conserved across the animal kingdom^16,17^, including in dipterans^14^. As expected, a large majority of cells expressed *elav-like* and lacked significant expression of *repo*, and were grouped in 40 neuronal cell clusters. In contrast, *repo* was expressed in a minority of cells belonging to three clusters (Fig. 1A, B). Additionally, two clusters were devoid of *elav-like* or *repo* expression, including Ag42, which expressed *collagen type IV alpha (col4a)* (Fig. 1A, B), an ortholog of the *D. melanogaster* marker (hereafter Dm markers) of hemocyte *col4a1*^18^. Neurotransmitter profiling identified widespread cholinergic and glutamatergic functions, while seven clusters were strongly GABAergic (Fig. 1B, Fig. S3A and Supplementary Data 1).

We next conducted a cross-species cell type mapping (SAMap)^6^ against a panel of six single-cell and single-nucleus RNA-seq datasets from brains of various insects, including three different datasets from the fruit fly *D. melanogaster*^19–21^, as well as atlases from the mosquito *Aedes aegypti*^7^, the honeybee *Apis mellifera*^8^ and the ant *Monomorium pharaonis*^5^. The cross-species comparison revealed cell type matches consistent with the evolutionary divergence^22–24^. Ag clusters had the highest similarity to *Ae. aegypti* clusters, followed by *D. melanogaster* and *A. mellifera* cell types, while they most differed from *M. pharaonis* ones (Fig. 1B, C). Most cell types were consistently matched across species (Fig. 1B, C and Fig. S3B, C, and Supplementary Data 2). We concomitantly identified marker genes for each cluster and genes with orthologs with known functions in other systems, and combined these results with the SAMap cell type assignment (Fig. 1D and Supplementary Data 1, 2). We leveraged cell type homology scores for the following cellular marker inspection. We observed a high concordance of our marker-based cell type assignment with the algorithm-predicted clusters (Fig. S3A, B and Supplementary Data 2, 3), and the analysis revealed some strong, consistent cross-species homologies (Fig. 1B, C). For instance, Ag41 was assigned with high confidence to Poxn neurons (Supplementary Data 2) and consistently was marked by expression of *poxn*^25^ (Fig. 1B). Similarly, general neural, glial and immune cell identity was confirmed by both marker analysis and cross-species cell type homology (Fig. 1A–C, Fig. S3, and Supplementary Data 2). Interestingly, Ag38 specifically expressed *scrb9*, a univocal marker of *An. gambiae* oenocytoids in hemolymph cells^26^, and did not have a consistent assignment to other insect species cell type (Fig. 1B, C). Altogether, our brain atlas provides the first comprehensive map of the cellular diversity in the *An. gambiae* female brain.

### Spatial organization of the female *An. gambiae* head

**Fig. 2:**
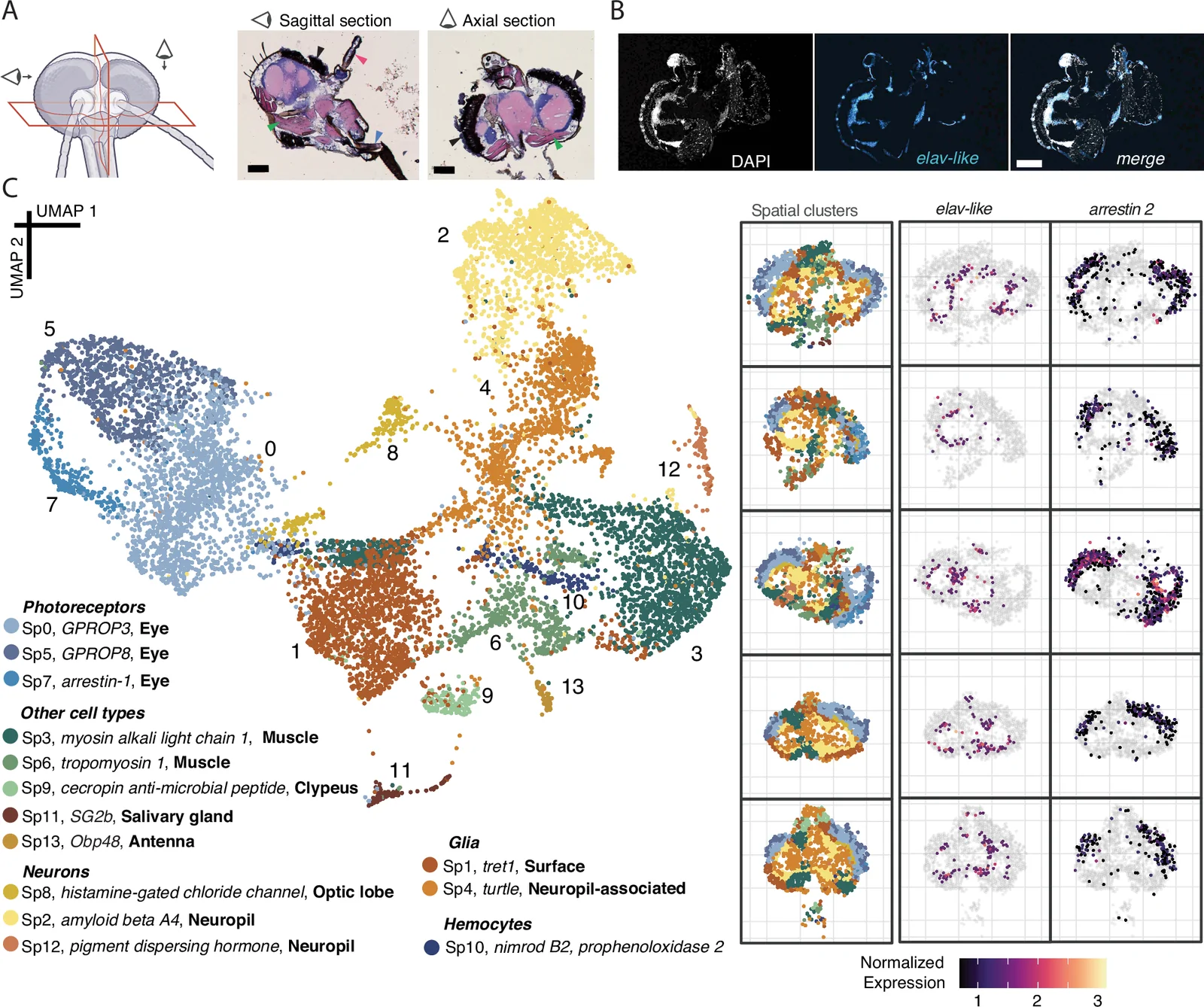
Spatial organization of the female *An. gambiae* head. **A** Schematic of sectioning orientation of mosquito heads. H&E staining of a sagittal and an axial cut of *An. gambiae* female heads; eye (black arrows), muscle (green arrows), antenna (magenta arrow), and proboscis (blue arrow) are annotated (scale bar = 10 µm). **B** ISH imaging of axial histological sections of a mosquito head (front of the head on the top, scale bar = 100 μm), highlighting nuclei (DAPI) and the neuronal marker *elav-like* (oriented with the front of the head on top and the right side of the head on the right). **C** Left panel: UMAP representation of 9, 914 high-quality spatially-resolved transcriptomes. The transcriptomes were clustered and grouped by the predicted major cell types (bold italics). For each Sp cluster, notable marker genes (*italics*) and likely tissue of origin (bold) are indicated. Right panel: Sp clusters displayed in their spatial context in five high-quality aligned head regions (oriented with the front of the head on top and the right side of the head on the right). Normalized expression of the neuronal marker *elav-like* and the eye-associated marker *arrestin 2* are displayed.

We next sought to define the spatial organization of the brain within the head of *An. gambiae* female, as its neuroanatomy has not yet been fully investigated. We initially used confocal microscopy with a presynaptic staining on dissected brains to understand the anatomy of the *An. gambiae* brains, and to contrast it to other dipterans. In addition to canonical insect brain structures^15,27^, including the optic lobes, the subesophageal zone and the central complex, the *An. gambiae* MB closely resembled that of *D. melanogaster*. It comprised conserved vertical and horizontal lobes that were further subdivided into distinct KC subclasses^28,29^ and subcompartments, likely innervated by different neuromodulatory neuron types, such as dopaminergic neurons^30,31^. We also identified notable differences relative to *D. melanogaster*. Although variation in the number of antennal lobe glomeruli (Fig. S4A) is common across insect species^32^, the organization of central complex substructures and the superior protocerebrum surrounding the MB vertical lobes^33^ (Fig. S4B) appear to be distinctive features of the *An. gambiae* brain. We next performed histological sections followed by hematoxylin and eosin (H&E) staining, and observed hematoxylin-stained cell body rind regions as well as eosin-stained neuropils (Fig. 2A). Brain regions were more readily observed in the axial sections, and this orientation was selected for further experiments. We used RNAscope in situ hybridization (ISH) on head sections and observed a wide distribution of the neuronal marker *elav-like* in soma-rich regions of the brain (Fig. 2B), marking the optic lobe underlying the eye as well as deeper cell body rind regions. To systematically understand the composition of the brain, we complemented high-resolution ISH with a lower-resolution (10 µm), but untargeted, Slide-seq spatial transcriptomic dataset of 8 female heads from two arrays^34^ (Fig. S5A). After quality control, we recovered 9,914 high-quality spatially-resolved transcriptomes (Fig. S5B), that could be grouped in 14 spatial clusters (hereafter Sp clusters) (Fig. S5C, D). We further subsetted, oriented, and aligned five high-quality head datasets in space using the consecutive H&E-stained slices (Fig. S5A). Using a consensus classification approach combining (i) marker gene analysis (Supplementary Data 2), (ii) mapping of the spatial clusters onto the cell atlas (Fig. S5E) with 66% of the spots matching to one or more single-cell clusters and 50% of which were assigned to a single-cell cluster, (iii) an inter-spatial cluster distance metric (Fig. S5F), and (iv) the spatial distribution of the Sp clusters (Fig. S6), we were able to assign a tissue and cell composition to most spatial clusters (Fig. 2C and Supplementary Data 2). Sp1 and Sp4 were associated with glia, while Sp10 was assigned a hemocyte ontology. Neurons were grouped in Sp2 and Sp12, marked by expression of *elav-like* and situated within the cell body rind region (Fig. 2B, C). Sp11 and Sp13 were located outside the brain (Fig. S5D), could not be associated to a single-cell cluster and were respectively marked by expression of *salivary gland protein 2-like* and *odorant binding protein 48*, and they were therefore respectively assigned to salivary gland and antennae tissue. Two other clusters (Sp3 and Sp6), positioned in the anterior and posterior parts of the head sections, were not associated to any Ag cluster and were marked by expression of multiple myosins (Supplementary Data 3), and were therefore assigned as muscle tissue. Sp9 was consistently situated at the front of the head and was assigned to clypeus (Fig. 2C and Fig. S6). Finally, the eye region was defined by several clusters (Sp0, Sp5, Sp7, Sp8), which corresponded to optic lobe and photoreceptor single-cell clusters and were situated in proximity to spots with expression of vision-associated genes, such as *arrestin 2* (Fig. 2C and Fig. S5E), a classical retinal protein^35^.

### A spatially-resolved map of cellular diversity in the *An. gambiae* brain

We next combined our cell atlas and our spatially-resolved data to determine the ontology of different cell populations within the brain, understand marker conservation and diversity, and identify important structures, thereby providing the first comprehensive transcriptomic cartography of the female *An. gambiae* brain.

#### Glia

**Fig. 3:**
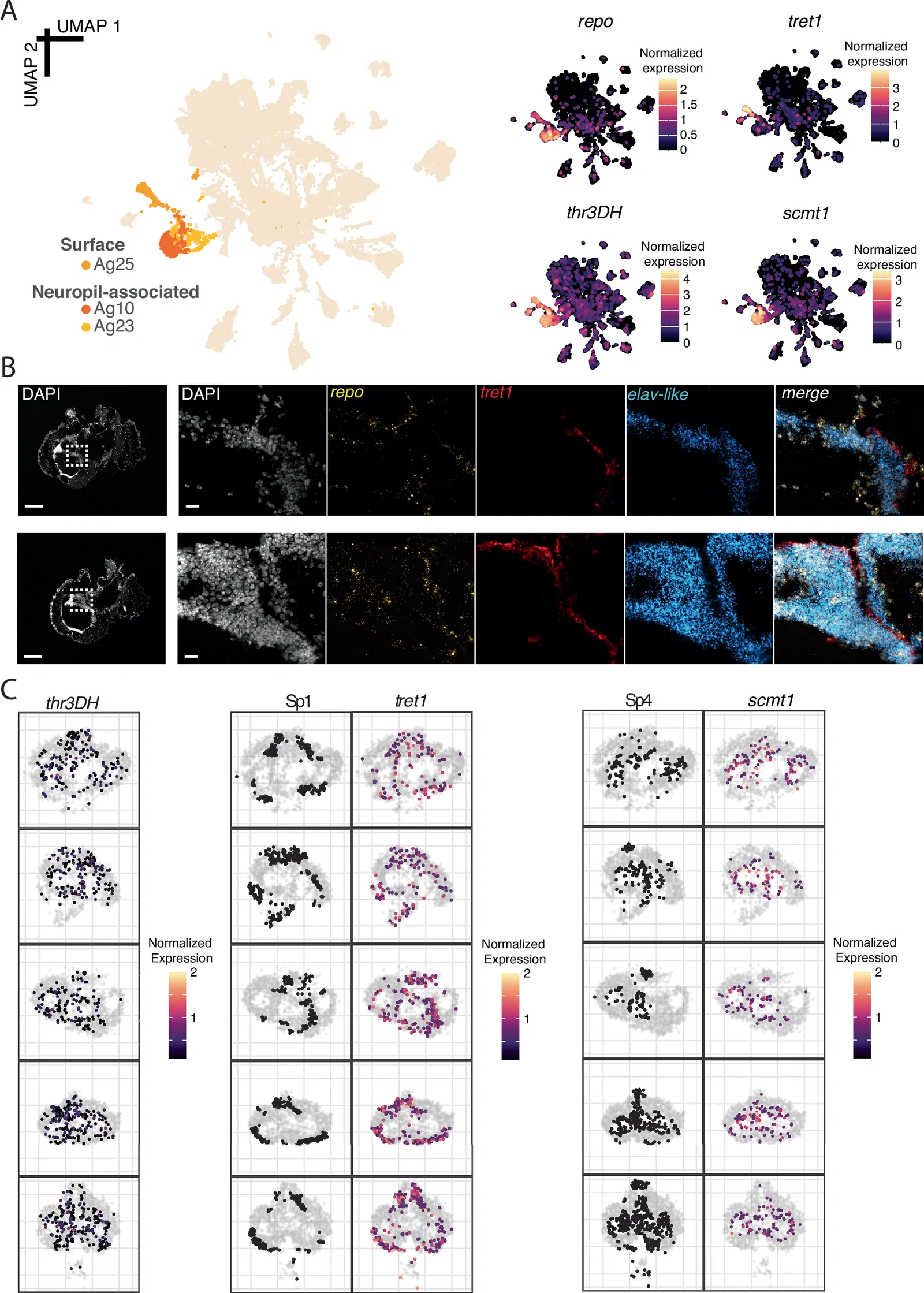
Diversity of glial cell types within the *An. gambiae* brain. **A** Annotated glia clusters and expression of generalist glia markers (*repo* and *thr3DH*), as well as surface glia (*tret1*) and neuropil-associated glia marker genes (*scmt1*). **B** ISH images of histological sections of mosquito heads. The whole section is shown on the left with nuclear staining (DAPI, scale bar = 100 μm, front of the head on top) and highlights the position of the inset that is displayed on the right; transcripts of glial marker genes (*repo* and *tret1*) are stained alongside those of the neuronal marker elav-like and nuclear staining (scale bar = 10 μm). **C** Five high-quality spatial sections (axial cuts, front of the head on top) highlighting the expression of generalist glial marker *thr3DH*, as well as the position of Sp glia clusters and reporter marker expressions associated with surface glia (Sp1 and *tret1*) and neuropil-associated glia (Sp4 and *scmt1*). Threonine 3 dehydrogenase: thr3DH; sodium-coupled monocarboxylate transporter 1: scmt1.

Glia are pivotal for neuronal development and function, and have been shown to be at the root of behavioral responses^21^. The three *repo*-expressing clusters Ag10, Ag23 and Ag25 were all strongly associated with glial cell types in the reference datasets (Fig. 1A–C and Figs. S3B, S7A). All glia clusters expressed *thr3DH (AGAP011948*) (Fig. 3A), an ortholog of Dm *CG5955*, which is involved in sleep regulation in the fly brain, a function strongly associated with glia^36,37^. Notably, all glial clusters expressed genes involved in lipid mobilization (Supplementary Data 3), a well-established glial function which fuels neural metabolism: they included *obp9*, an ortholog of Dm *obp44a* with a role in lipid trafficking in the astrocytes of the fly^38^, and *lipocln* (*AGAP005223*), an ortholog of Dm *glaz*, known to work as a glia-neuron lipid shuttle^39^ (Fig. S7B–D).

Surface glia have a unique role of insulating the brain from the hemolymph forming a blood-brain barrier. SAMap associated Ag25 to glia, perineurial and sub-perineurial glia (Supplementary Data 2), and the cluster expressed surface glia markers: *tret1*, an ortholog of Dm *tret1-1*, a marker of perineurial glia in the fly brain^40^, as well as *ABCB2 (AGAP005639)*, an ortholog of Dm *mdr65*, a marker of sub-perineurial glia^41^ (Fig. 3A and Fig. S7D). We used ISH to determine the distribution of *tret1-*expressing cells. Our probe panel measured RNA signals from *elav-like*, *repo* and *tret1* transcripts. We identified a dense neuronal cell-body rind using the *elav-like* signal and observed that *repo*-expressing cells were broadly distributed, primarily within the soma-rich region. In contrast, *tret1* had a continuous distribution on the surface of the cell body rind (Fig. 3B), suggesting that the *An. gambiae* brain is insulated by surface glia expressing *tret1*. Interestingly, the Sp1 spatial cluster partially matched Ag25 and was marked by surface glia markers (Supplementary Data 2). Despite an only partial overlap of Sp1 with *tret1* expression, the spatial cluster was distributed peripherally within the mosquito head (Fig. 3C), and located at the immediate proximity and at the surface of the cell body rind Sp2 (Figs. S5F, 6). We concluded that Ag25 and Sp1 confirm the presence and distribution of surface glia in the *An. gambiae* brain. Interestingly, we could not differentiate within Ag25 between perineurial and subperineurial glia, with more than a third of Ag25 cells expressing both *tret1* and *ABCB2*. This suggests a possible divergence in functional sub-compartmentalization between mosquito and fly surface glia.

The ISH distribution of *repo* confirmed the presence of glial cells within the cell body rind and the neuropil (Fig. 3B). Concordantly, Sp4 was associated with Ag10 and was distributed within both cell body rind and neuropil regions, and mostly mirrored the expression pattern of *scmt1* (*AGAP001185*) (Fig. 3B, C), an ortholog of the neuropil-associated glia marker Dm *rumpel*^42^. SAMap evidenced a strong similarity of Ag10 to *D. melanogaster* ensheathing, cortex and astrocyte-like glia, while Ag23 was mapped to astrocyte-like glia (Fig. S3B, Fig. S7, Supplementary Data 2). In addition, both of these single-cell clusters expressed known glial markers (Fig. 1B, Fig. S7B–D, and Supplementary Data 1). However, they did not align with specific markers of glia cell type in *D. melanogaster*, and we observed co-expression in the same cell of markers known to be exclusive of specific cell types in the fruit fly. For instance, we observe co-expression of orthologs of Dm *ebony* (*ebony*) and *rumpel* (*scmt1*), respectively markers of astrocyte-like glia^43^ and ensheathing glia^42^, in 45% of the cells in Ag10 and Ag23, as well as the wide expression of *turtle* in Ag10, an ortholog of Dm *bdl*, a marker of wrapping glia^44^. This suggests that functional markers of *D. melanogaster* neuropil-associated glia are not conserved in *An. gambiae*. However, we capture a rich transcriptional diversity within Ag10 and Ag23, and identify markers of different cell types (Supplementary Data 1, 3), which may be leveraged to understand unique glia biology in the mosquito brain.

#### Vision and circadian neurons

**Fig. 4:**
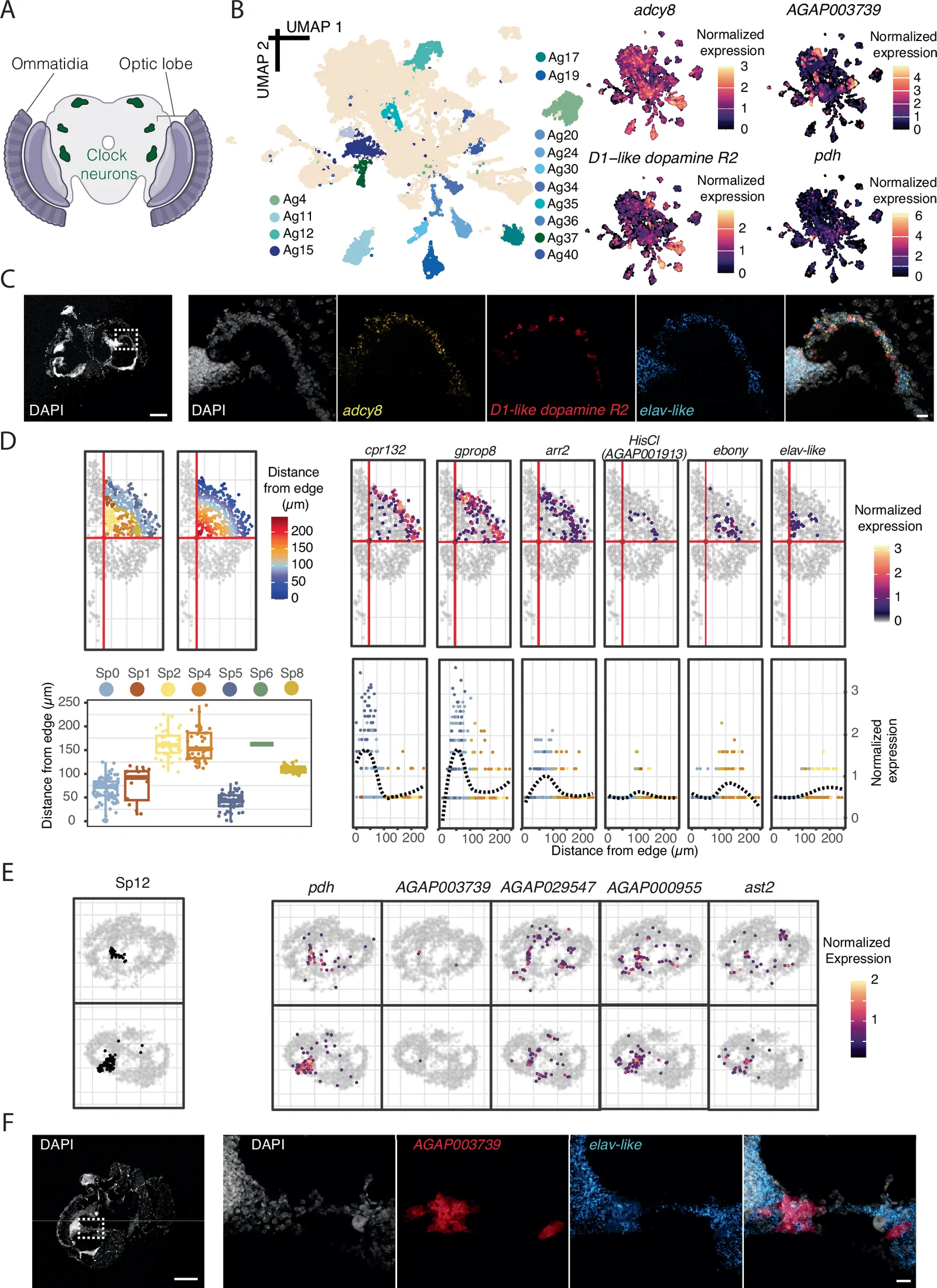
Optic lobe and circadian neurons in the visual system of *An. gambiae.* **A** Schematic of the expected positions of optic lobes and clock neurons in *An. gambiae* brain. **B** Annotated optic lobe and clock neuron-enriched clusters and expression of optic lobe markers (*adcy8* and *D1-like dopamine R2*), as well as clock neuron markers (*AGAP003739* and *pdh*). **C** ISH images of histological sections of mosquito heads. The whole section is shown on the left with nuclear staining (DAPI, scale bar = 100 μm, front of the head on top) and highlights the position of the inset that is displayed on the right; transcripts of optic lobe marker genes (*adcy8* and *D1-like dopamine R2*) are stained alongside those of the neuronal marker *elav-like* and nuclear staining (scale bar = 10 μm). **D** One section of a head, showing the distribution of clusters within this section as well as the distance of spots to a centrally-set reference. This reference was used to compute gene expression distribution by distance in the eye structure and the optic lobe of the mosquito and to identify genes with a distance responsive distribution (from the edge to the reference center point (red)) which may be involved in the visual system, example in situ gene expression patterns as well as distance computed distribution are displayed in the right panel, the full complement of distance responsive genes is given in Table S4.Each dot represents one spatial spot within the selected section. Each box represents the median and first and third quartiles per cluster with the whiskers indicating the most extreme data point, which is no more than 1.5 times of the interquartile range. **E** Distribution of Sp12 in two high-quality heads as well as normalized expression of five Sp12 markers (Table S3). **F** ISH images on histological sections of mosquito heads. The whole section is shown on the left with nuclear staining and highlights the position of the inset that is displayed on the right (bar = 100 μm, front of the head on top); transcripts of clock neurons marker gene *AGAP003739* are stained alongside those of the neuronal marker *elav-like* and nuclear staining (scale bar = 10 μm). Histamine-gated chloride channel: HisCl.

The insect visual system occupies a big volume in the brain (Fig. 4A) and consists of well-defined regions (photoreceptors, lamina, medulla, lobula complex), that are composed of distinct cell types^45–47^. Our single-cell atlas identified several putative vision-associated cell types with defined marker expression (Fig. S8A,C and Supplementary Data 2). They could be grouped in broad categories, including photoreceptors (Ag15) as well as various optic lobe-associated neurons comprising (i) lamina neurons (Ag11, Ag19 and Ag24), (ii) putative proximal medulla interneurons (Pm) (Ag40), (iii) putative C2/C3 cells (Ag39), and (iv) uncategorized optic lobe neurons (Ag4, Ag12, Ag17, Ag30, Ag34, Ag35, and Ag36). We also determined that the expression of either one of two markers identifies different sub-populations of the optic lobe neurons: *adcy8* (Dm *rut*) (Ag11, Ag19, Ag30, Ag36) and *D1-like dopamine R2* (Ag17, Ag24) (Fig. 4B). To validate the annotation and these marker genes, we performed multiplex ISH of these two markers in regions where both the ommatidia and the cell body rind (*elav-like* positive) were discernible, and showed that both markers were present in the lamina with distinct expression patterns, confirming them as appropriate markers of these cell populations and the annotation as optic lobe cell types (Fig. 4C). Interestingly, our spatial transcriptomic dataset captured the expression pattern of the whole eye from photoreception (Sp0, Sp5, Sp7) to signal integration in the lamina (Sp8) (Fig. 2C), and displayed a highly structured pattern (Fig. 4D). We reasoned that we could use these data to identify genes related to vision and signal integration. We therefore selected three regions where the tissue sectioning captured the full transition from the lens through the ommatidia and to the optic lobe neurons' soma in the cell body rind, and performed an expression-by-distance analysis to identify and contextualize vision-associated genes (Fig. 4D and Fig. S9). Using this approach, we identified 103 candidate genes linked to the eye region (Supplementary Data 4). These ranged from eye-specialized cuticular protein (e.g.,* cpr132*)^48^, peripheral opsins in the photoreceptor cell (e.g.,* gprop8*)^49^, through signal transduction factor (e.g.,* arr2*)^35^ to signal integration in the lamina (e.g.,* HisCl AGAP001913*, *elav-like*) (Fig. 4D, E). Opsins are key to photoreception and have been shown to have expanded in different dipterans^49^ but their specific role is unclear in *Anopheles*^50^. Here, we detected five dominantly expressed opsins (*gprop1, 3, 4, 6, 8*) in photoreceptors (Ag15) (Supplementary Data 3) and within the ommatidia (Figs. S8D, S9 and Supplementary Data 4). Among those there are four representatives of the *Rh6* (long wavelength sensitive *gprop1, 3, 4, 6*) that are responsive to green light in *Drosophila* species but have been shown to have undergone diversification in *Anopheles*^49^, and a single putative *Rh3/4* ultraviolet wavelength-sensing opsin.

Vision is intimately linked to circadian rhythms and is usually mediated by specialized interregional neurons with projections spanning multiple neuropils. Interestingly, Ag11 and Ag30 were characterized by *AGAP001726*, an ortholog of Dm *wake*, a clock gene expressed in latero-ventral clock neurons (LNvs)^51^. Although lamina and LNvs have different tissue localization and roles, lamina neurons have intimate interaction with clock neurons and express clock genes in *D. melanogaster*^52–55^. Circadian clock cell type composition in *D. melanogaster* includes approximately 150 neurons per brain, organized into lateral neurons and dorsal neurons^56^. Ag37 was strongly associated with D. *melanogaster* clock neurons (LNvs) in the multispecies dataset panel (Fig. S8A and Supplementary Data 2). In addition, Ag37 was strongly marked by *AGAP003739* (Dm *fer2*) (Fig. S8B and Supplementary Data 1), which is expressed in protocerebral anterior medial/lateral neurons and in LNvs^57,58^. The cluster was marked by *gprcal3* (Dm *pdfr*), and a subset of cells in the cluster expressed *zic* (Dm *opa*) and *pdh* (Dm *pdf*), confirming a clock neuron cell identity^56,59^. Furthermore, Ag37 expressed *cardioacceleratory peptide* (Dm *ccap)* (Supplementary Data 3), which is involved in the regulation of circadian behaviors^60,61^. In our spatial data, Sp12 was annotated as a clock neuron-enriched cluster (Supplementary Data 2). Sp12 was present in only two high-quality head sections, and was positioned in a lateral anterior focus within the cell body rind. The other heads were likely sectioned in a different plane devoid of Sp12 (Fig. 4E), suggesting that the distribution of cellular bodies of clock neurons has a spatially restricted focus in the *An. gambiae* brain. In agreement with these observations, we noted ISH foci of *AGAP003739*, one of our clock neuron markers, localized in soma-rich structures, in continuity with those lining the outer optic lobe (Fig. 4F). This location matches the one described for subsets of *D. melanogaster* clock neurons^51^ (Fig. 4A). Altogether, our data showed a cellular population enriched in clock neurons (Fig. S8B), with a focal location within the *An. gambiae* brain.

#### Kenyon cells

MBs are paired structures in insect brains, including *Anopheles*’^15,33^ (Fig. S4), involved in olfactory associative learning, memory and goal-directed behavior^62^. The MBs are primarily composed of KCs (Fig. S10A). *D. melanogaster* (Diptera) KC axons typically form horizontal and vertical α/β, α′/β′, and γ lobes, but can be further functionally subdivided into seven subtypes with a likely unique molecular profile^30^. *A. mellifera* (Hymenoptera) instead possesses five major KC types, divided in Class I (sKC, mKC, ℓKC types), and Class II (II-KC1 and II-KC2 types)^63^. We identified three distinct KC types of the *An. gambiae* MBs: Ag14, Ag28, and Ag32 were associated with KC clusters in the cross-species comparison (Fig. 1B, C and Fig. S3B) and expressed multiple KC-associated genes and markers (Fig. S10B–E and Supplementary Data 1–3). Ag28 expressed *AGAP000585* the ortholog of Dm *mamo* which specifies a cell’s fate in *D. melanogaster* α′/β′- and γ-KC^64,65^ (Fig. S10B, E and Supplementary Data 2); it showed the greatest similarity to *D. melanogaster* α′/β′ KCs and *Ae. aegypti* KCs, and low similarity to *A. mellifera* KC subtypes (Supplementary Data 2). We further noted that Ag28 and Ag32 were marked by *AGAP029446*, an ortholog of Dm *trio*, a marker of *D. melanogaster* α′/β′ and γ KCs^66^. Ag14, Ag28 and Ag32 were also marked by expression of genes important for MB development such as *drn-tre (*Dm *rn-tre*)^67^ and for memory formation in MBs, such as *nuclear receptor subfamily 2 group E member 3* (Dm *hr51*), *slingshot* (Dm *ssh*), *adcy8* and *D1-like dopamine R2* (Dm *dop1R2*), *gprdop1* (Dm *dop1R1*), and multiple protein kinase A subunits (*AGAP029637*, *AGAP006448* and *AGAP004940*)^68–74^ (Fig. S10C, E and Supplementary Data 3). Although not individually aligned with different KC subtypes in the reference datasets, the aforementioned transcriptional signature suggests conserved KC functions in the *An. gambiae* brain, including MB neuroblast proliferation (e.g.,* mamo*^64^), axon growth and guidance, and MB neural network wiring (e.g.,* trio*, *drn-tre*^66,67^). We further identified a small subcluster of Sp2 that was characterized by the expression of markers of Ag14, Ag28, and Ag32 (Supplementary Data 3). We isolated these 18 spots (Fig. S10F) and conducted a marker analysis, identifying 11 marker genes,eight of which were shared with KC single-cell clusters (Supplementary Data 5). These spots clustered in a single focus within the brain, which expressed KC markers and therefore corresponds to an *An. gambiae* MB. Mu and colleagues observed high similarity of hymenopterans (i.e*., A. mellifera* and *M. pharaonis*) sKCs and II-KC1 types with dipterans KCs (i.e*., D. melanogaster*)^8^. In concordance, our analysis showed that KCs of *An. gambiae* (Diptera) have the highest similarity to *A. mellifera* sKCs and II-KC1 types; however, we could also observe relatively high similarity of the KC cluster Ag14 to *A. mellifera* mKCs (Fig. S3 and Supplementary Data 2). Altogether, we defined three distinct KC types of the *An. gambiae* MBs associated with distinct and *An. gambiae*-specific transcriptional profiles.

#### Immune cells

Three types of hemocytes are described in *Anopheles*: the phagocytic granulocytes, the melanin-producing oenocytoids, and the prohemocytes, possible precursor cells of the two mature hemocyte types^75^. Ag42 was associated with *Drosophila* plasmatocytes and expressed numerous *An. gambiae* granulocyte markers, including *lrim16a*, *lrr8*, *col4a* and *sparc*^26^ (Fig. S11A–D, Supplementary Data 1). Ag38 specifically expressed *scrb9*, a univocal marker of *An. gambiae* oenocytoids among immune cells^26^ (Fig. S11B and D), and was the only cluster showing a purely dopaminergic profile (Fig. S3A) which is consistent with its immune function^76^. We did not identify prohemocytes, suggesting there is no hematopoietic differentiation in and surrounding the brain. Immune cells were also captured in our spatial data (Sp10), and were distributed throughout the head samples, but showed a preferential proximity to head muscles (Sp3, Sp6) and glia cells-containing foci (Sp1, Sp4) (Fig. 2C and Fig. S5F). We further confirmed granulocyte presence in the *An. gambiae* female head by ISH, which consistently showed *col4a*-positive cells associated with *elav-like*-negative nuclei at the back of the mosquito head (Fig. S11E). Interestingly, the ISH data also suggested that granulocytes can locate between the lamina and the ommatidia (Fig. S11E). Altogether, our results show for the first time mosquito immune cells located in the brain periphery, at the limit of the insect blood-brain barrier, and are consistent with a recruitment of mature hemocytes from the hemolymph compartment^77,78^.

#### Other cell types and structures

Ag0 expressed markers suggestive of a progenitor state (Supplementary Data 1) such as *numb*, which in *D. melanogaster* controls asymmetric division in sense organ precursors^79,80^ and planar cell polarity establishment in eyes^81^, suggesting a possible optic lobe progenitor cell type.

We reasoned that the tight packing of the cell body rind in combination with current technical restrictions of spatial transcriptomics could lead to multiple cell types being captured in the Sp2 cell body rind cluster and render the identification of specific structures difficult, especially those composed of a few neurons in each brain. To establish whether we could recover specialized structures within the cell body rind, we selected two marker genes of cell clusters captured in the atlas, *vmac*, among the top ten markers of Ag7 (enriched with monoaminergic/peptidergic neurons), and *mip*, a marker of MIP-neurons expressed in Ag4, which can regulate feeding and mating behaviors^82,83^. Robust expression of these markers identified sub-clusters within Sp2 (Fig. S12A, B), which had a discrete localization in the head, suggesting monoaminergic/peptidergic and MIP neurons mark discrete structures within the brain of *An. gambiae* as in *D. melanogaster*^83,84^.

### Life history is associated with distinct brain transcriptional states

**Fig. 5:**
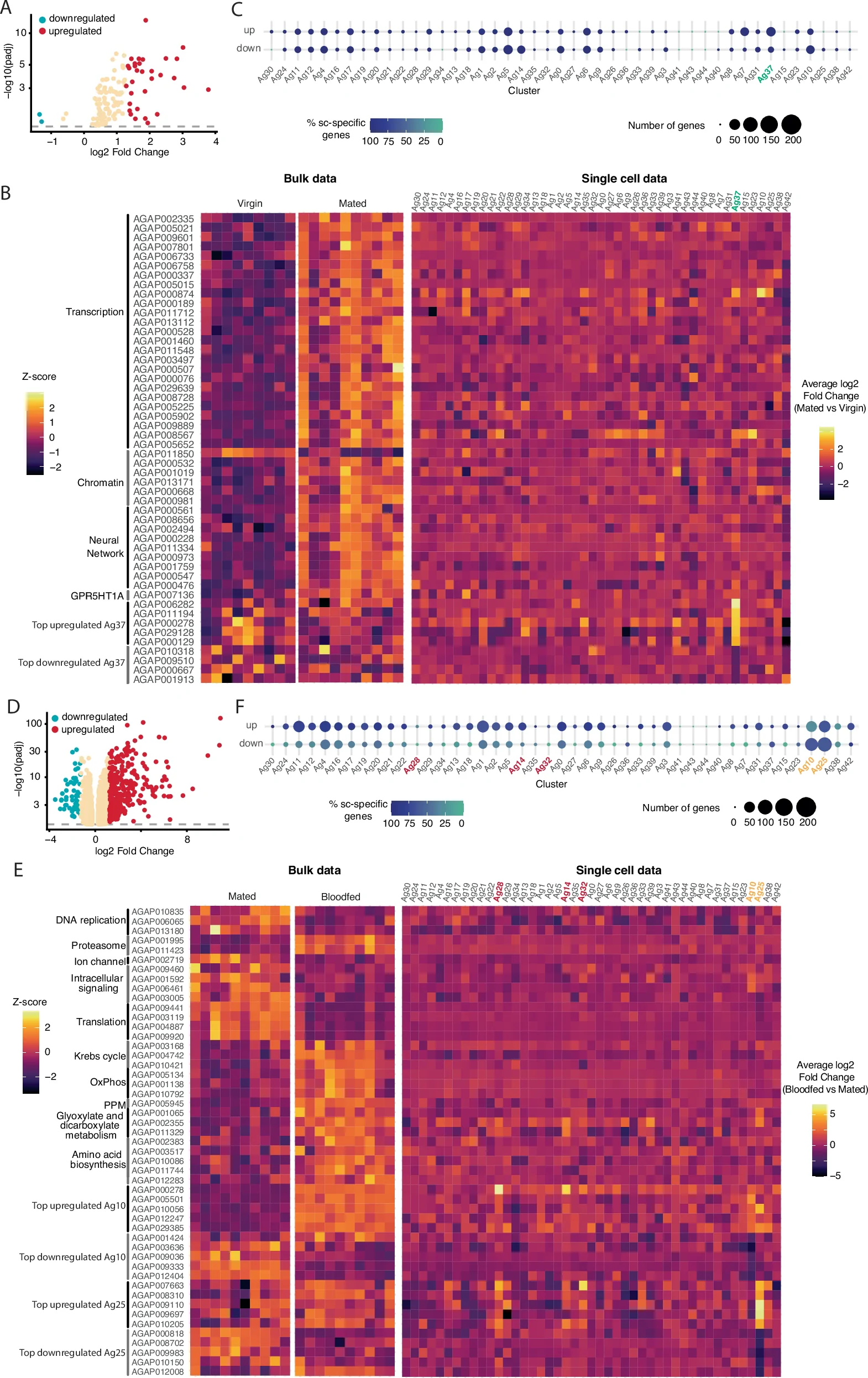
Different biological states in *An. gambiae* females are associated with distinct brain transcriptional states. **A** Volcano plot of DEGs (Benjamini-Hochberg (FDR) adjusted p-value < 0.05) between virgin and mated females; genes having absolute logarithmic fold-change > 1.25 are highlighted (red and blue). **B** Heatmap of selected genes from (**A**), grouped according to broad gene functions curated manually and based on gene ontology and KEGG pathway enrichment, and of top brain DEGs between virgin and mated mosquitoes in scRNA-seq clusters (clock neuron-enriched cluster, Ag37, in green). **C** Number of genes up and downregulated after mating (compared to a virgin status) in each of the clusters of the single-cell brain atlas, colored based on the percentage of genes that are DE in each cluster, but not DE in the single brain bulk RNA-seq data (% sc-specific genes). **D** Volcano plot of differentially expressed genes (Benjamini-Hochberg (FDR) adjusted *p*-value < 0.05) between mated sugar-fed or mated blood-fed females; **E** Heatmap of selected genes from (**D**) grouped according to broad gene functions curated manually and based on gene ontology and KEGG pathway enrichment, and of top brain DEGs between mated and blood-fed mosquitoes in scRNA-seq clusters (KC clusters in red, glial cell clusters in yellow). **F** Number of genes up and downregulated after bloodmeal (compared to a mated status) in each of the clusters of the single-cell brain atlas, colored based on the percentage of genes that are DE in each cluster, but not DE in the single brain bulk RNA-seq data (% sc-specific genes). Bulk RNA-seq data were obtained from three experimental conditions, per *N* = 1 experimental replicate, for a total of *n* = 29 single brain samples. ScRNA-seq data were obtained from three experimental conditions, per *N* = 2 experimental replicates, for a total of *n* = 60 pooled single brain samples. Putative serotonin 5HT-1a receptor: 5HT-1a R; oxidative phosphorylation: OxPhos; purine and pyrimidine metabolism: PPM.

The different life phases of adult female anophelines are associated with distinct behaviors such as mating or host-seeking, which are likely supported by distinct neuro-transcriptional states^8,85,86^. Typically, a virgin female will mate prior to blood feeding and may become infected by malaria parasites upon a bloodmeal on an infectious human. In the case of *An. gambiae* infection by *P. falciparum*, it will then take ~ 10–14 days for sporozoites to colonize the salivary glands and render the mosquito infectious to another human host, so that only older mosquitoes can transmit parasites^87^. We reasoned that these states may vary significantly among individuals, and therefore developed a protocol for single-brain RNA-seq (Fig. S1B). We first compared brains from virgin and mated females. Almost all the 121 differentially expressed genes (DEGs) in whole brains were upregulated after mating (Fig. 5A, Supplementary Data 6), including transcription factors with orthologs involved in circadian rhythm and behavioral regulation, cell fate specification, and axon guidance^88–90^, chromatin repressors and neural network remodelers^91,92^, suggesting that mating triggers active rewiring and signaling in the female mosquito brain (Fig. 5B). In addition to that, we identified a post-mating upregulation of a putative receptor for serotonin (GPR5HT1A), a neurotransmitter associated with mating refractoriness in *D. melanogaster*^93^. We further conducted differential expression analysis (DEA) in our scRNA-seq data, which revealed that neuronal clusters account for most of the transcriptional shift (Fig. 5C and Supplementary Data 7). In particular, mating appears to induce a strong downregulation of *cabut* (*AGAP009889*) in Ag37 (clock neurons-enriched), this gene has previously been shown to be involved in circadian clock activity and energy metabolism in response to sugar sensing in *D. melanogaster*^90^, as well as downregulation of malate dehydrogenase gene (*AGAP009510*), and of genes involved in signaling, such as guanine nucleotide binding protein subunit (*AGAP010318*), *D1-like dopamine R2*, and *HisCl AGAP001913*. Mating was associated with an upregulation of genes with orthologs involved in cell growth and adhesion, such as *AGAP006282* (Dm *Fs*), *AGAP011194* (Dm *klg*, a cell adhesion molecule) and *AGAP029128* (Dm *mfas*, involved in axonogenesis), as well as *obp9* and the aminopeptidase *AGAP000129*. We also note the upregulation of some genes classically involved in light signal processing, such as *arrestin-1* (*AGAP010134*), *GPROP1* (*AGAP013149*), *GPROP3* (*AGAP012982*), and *GPROP4* (*AGAP012985*) in Ag37.

We then compared the brains of age-matched sugar-fed mated females with those of mated females 24 h after a bloodmeal (Fig. 5D). Single brain differential expression analysis identified 1, 895 DEGs (Fig. 5D and Supplementary Data 6). Blood feeding induced a profound alteration in genes associated with translation activity. This was evidenced by an overall downregulation of genes encoding ribosomal proteins, translation initiation and elongation factors, and tRNA synthetases, suggesting a decrease in translation processes in the brain following blood feeding. This was also associated with an increase in transcription of proteasome proteins further pointing to a reshaping of proteostasis (Fig. 5E and Supplementary Data 6). A concomitant downregulation of genes involved in cell proliferation and intracellular signaling (e.g., putative GPCR receptors, kinases of the MAPK cascade, Ser/Thr kinases, ion channels) could indicate a lowered response to stimuli, consistent with the refractory/resting interval after a bloodmeal^94^ (Supplementary Data 6). On the other hand, we saw increased expression of genes associated with energy metabolism, such as enzymes of the purine, pyrimidine, amino acid biosynthesis, glyoxylate and dicarboxylate metabolism, the Krebs cycle, and oxidative phosphorylation after bloodmeal (Fig. 5E and Supplementary Data 6). Interestingly, we identified an odorant binding protein gene, *obp9* (*AGAP000278*), strongly upregulated following bloodmeal (Fig. 5E and Supplementary Data 6), consistent with previous proteomics studies on whole *An. gambiae* heads^95^. This gene is a robust marker of neuropil-associated glia (Ag10) in our atlas (Fig. S7 and Supplementary Data 3). The processes triggered by bloodmeal showed consistent trends in bulk and scRNA-seq data (Fig. 5E, F and Supplementary Data 8). The DEA per-cell cluster revealed a drastic glial transcriptional switch after bloodmeal, consistent with what has been observed for *Ae. aegypti*^7^. The top upregulated genes in glia clusters Ag10 and Ag25 are hemolymph proteins, and unexpectedly showed a similar pattern in KC clusters (Fig. 5E). For instance, *obp9* expression was strongly induced by bloodmeal in KCs, together with *AGAP007663*, an ortholog of multiple 27 kDa hemolymph protein-like genes in *Aedes* and *Anopheles* species. Blood feeding also enhanced immunity genes expression in the brain with upregulation of genes coding for (i) peptidoglycan recognition proteins (*pgrps1*, *pgrps3*) (ii) the Toll-pathway activator *spz1*, pro-inflammatory TNF-α receptor ligand *eiger*^96^ (iii) melanization (CLIP domain serine proteases, *srpn2*, *yellow protein*) (Iv) genes coding for antimicrobial peptides (*cec1*, *def1*)^97^ and (v) extracellular matrix-reshaping protein coding genes (Supplementary Data 6), which are involved in cell migration^98,99^. These genes are predominantly expressed by the hemocyte and the glia clusters in the cell atlas (Fig. S13A), with *clipB13* (*AGAP004855*) being significantly upregulated after bloodmeal in both Ag10 and Ag25 glia clusters (Supplementary Data 8), suggesting an intrinsic brain immune response to blood-feeding.

**Fig. 6:**
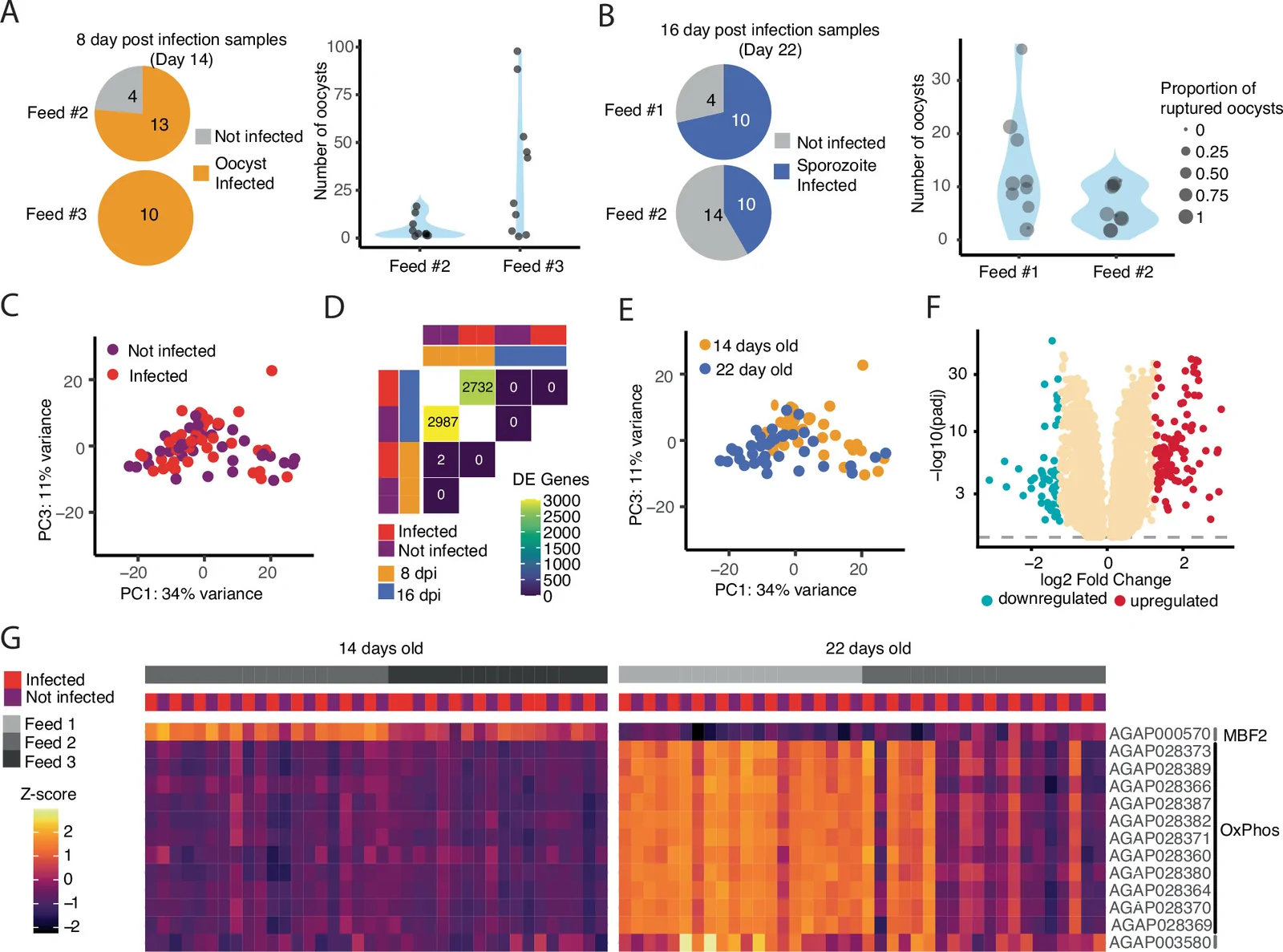
Impact of *Plasmodium falciparum* infection on the female *An. gambiae* brain. **A** Prevalence and intensity of infection were scored during sample dissection in female *An. gambiae* mosquitoes 8 days after the infection challenge (14 day-old individuals). **B** Prevalence and intensity of infection scored during sample dissection in female *An. gambiae* mosquitoes 16 days after the infection challenge (22 day-old individuals). **C** PCA of single brains transcriptomes from infected or non infected females. **D** Distance plot showing the number of DEGs found in one-to-one infection condition comparisons (infection status and age) (*ashr* method). **E** PCA of single brains transcriptomes from 14 or 22-day-old females. **F** Volcano plot of differentially expressed genes (adjusted *p*-value < 0.05) between 14 and 22-day-old females; genes having absolute logarithmic fold-change > 1.25 are highlighted (red and blue). **G** Heatmap of top differentially expressed genes from (**F**) (|log_2_FC | > 1.5 and FDR < 1E-20). Data were obtained from four experimental conditions, per *N* = 2 experimental replicates per condition, for a total of *n* = 78 single brain samples. Multiprotein bridge factor 2: MBF2; Oxidative phosphorylation: OxPhos; purine and pyrimidine metabolism: PPM.

Behavioral manipulation by parasites is a widespread adaptive strategy and several studies have shown that malaria parasites can alter mosquito feeding behavior in a way that favors their transmission^10–12^. We considered that such a manipulation would likely have effectors in the brain of the mosquito. We generated single brain transcriptomes of *P. falciparum*-infected and uninfected mosquitoes, with a wide range of infection intensities (Fig. S1C and Fig. 6A, B). We compared infected mosquitoes to their non-infected counterparts at (i) 8 days post infection (dpi), when parasites are at the oocyst stage and are not infectious, and (ii) 16 dpi, when parasites are at the infectious sporozoite stage in salivary glands. Surprisingly, our analysis revealed no consistent transcriptional difference at either time point in the brain transcriptional profile, with only *def1* being upregulated at 8 dpi when replicates are combined (Fig. 6C, D and Supplementary Data 6). All the infected mosquitoes had the same age when challenged with *P. falciparum* (i.e., 6 days old), and were dissected with their non-infected controls either 8 or 16 days later, when they were 14 and 22 days old, respectively. We therefore explored the influence of aging on the brain and identified a robust transcriptional difference between day 14 and day 22 brains independent of infection status (Fig. 6D–F, Supplementary Data 6). Interestingly, we observed signals known to be associated with aging in other systems. For instance, insulin-like peptides, associated with age-related nutrient sensing^100^, were all upregulated (7/7) in older mosquitoes (Supplementary Data 6). Additionally, immunity impairment with age is well established^101^ and we evidence an upregulation of CLIP serine proteases (FDR = 2.92E-07) and serpins (FDR = 3.72E-03) (Fig. S13), which have a role in modulating inflammation^102^, and are likely induced by immune cells and glia (Fig. S13B, C). However, the top upregulated genes with age were mainly linked to oxidative phosphorylation (OxPhos), genes that are expressed in most cell types (Fig. 6G and Fig. S13D). This result contrasts with a usual decline in OxPhos gene expression with age in insects^103,104^. Our observations may reflect an early compensatory mechanism linked to the onset of impaired mitochondrial function in the aging brain^105^. Taken together, these data suggest that aging in the female *An. gambiae* brain is initiated by a dysregulation of both mitochondrial function and immunity.

## Discussion

In this study, we present a survey of cell type composition and tissue gene expression patterns of the brain of the primary malaria vector. We confidently identified broad cell type categories (e.g., glia, KCs) within the *An. gambiae* brain. For many of them, we could ascribe a more granular identity. In some cases, cross-species homologies were supported by shared ortholog marker genes (e.g., surface glia, monoaminergic/peptidergic neurons), while other cell types possessed a signature specific to the *Anopheles* brain among the contrasted species (e.g., neuropil-associated glia). For all *Anopheles* cell types, we also defined marker genes, some of which show species-transcendent patterns while others show species-specificity. This represents a foundational map of the female *Anopheles* brain, supporting future functional characterization of neuronal populations and further characterization of known and novel cellular mechanisms in insect neurobiology.

We determined the diversity and spatial organization of the major cell populations of the brain, namely glial cell types, optic lobe neurons, immune cells, KCs, clock neurons and other central brain cell types, using high-resolution ISH of selected candidate markers and leveraging univocal assignments of 50% of the spatial transcriptomics spots to Ag clusters, combined with putative marker expression patterns. Our study lays the ground for future studies to hopefully comprehensively characterize gene expression in the *Anopheles* brain with a high-resolution 3D localization mapping.

A large cell population displays neuroblast-like features (Ag0), suggesting that it is composed of neural progenitors. While neurogenesis has been shown to take place in the brain of some adult insects both in physiological conditions and after injury^106–109^, to our knowledge it has not been reported in an adult mosquito brain.

Despite the evolutionary divergence between *Anopheles* and established model organisms, our data revealed broadly conserved gene expression and spatial organization consistent with patterns previously described in other species. Some clusters appear to be specific to *Anopheles* (e.g., Ag13), highly similar among mosquitoes (e.g., Ag29, Ag31, Ag43), shared across Diptera (e.g., Ag41), or conserved across orders included in this analysis (e.g., Ag25). Furthermore, we observed that dipteran species share a broadly concordant distribution of KC subtypes, whereas hymenopteran species exhibit KC subtypes (e.g., ℓKCs and II-KC2) that we did not detect in the mosquito samples. This is consistent with the hypothesis that specialized behavioral or sensory functions in Hymenoptera may be supported by lineage-specific cell types, although further sampling and functional validation would be required to confirm this interpretation. Such multi-species cell homology comparison may offer preliminary insight into the functional evolutionary history of insect nervous systems.

Our adult life-stage-resolved single-brain data mapped expression changes linked to transmission-related life history and highlighted that mating induced subtler variations in brain transcription than the massive shift caused by a bloodmeal. Interestingly, we show that mating induced alterations in gene expression associated with neural functions and signaling, including upregulation of light-perceiving genes in clock neurons. The mating-associated upregulation of these transcripts suggests that photic input to the circadian network may be dynamically tuned by reproductive state. Although this is consistent with established direct light sensitivity of deep brain regions and clock neurons in *Drosophila* and vertebrates^110,111^, functional validation will be required to demonstrate active light transduction in *An. gambiae* clock neurons. As *An. gambiae*, and many other mosquito species, have a tendency to mate at a specific time of the day (namely, at dusk), this finding opens new questions on whether and how light perception by central brain neurons can drive mating behaviors.

In our data, gene expression 24 h post-bloodmeal is consistent with programmed resting^94^. Switching from sugar to blood digestion corresponds to a massive shift in the amount and type of available metabolic resources. While previous studies identified a general downregulation of metabolism-related genes at the organism level^112^, our results show a metabolic switch in the brain that contrasts with the systemic responses to bloodmeal, and may support highly energy demanding processes such as nutrient metabolism and memory consolidation^113,114^. Our data also advocate for hormonal cross-talk or nutrient exchange guided by glia, and suggest KCs activate similar processes: the strong upregulation of *obp9* in both types of cells suggests it might have a non-olfactory role, perhaps associated with lipid transport, as has been hypothesized for the *obp9* ortholog in *Ae. aegypti*^115^.

Moreover, our data ruled out an extensive transcriptional response to parasite infection in mosquito brains. We can hypothesize that parasitic manipulation did not occur in our parasite-mosquito strains combination and/or lab conditions, knowing that parasitic manipulation in the *An. gambiae* - *P. falciparum* couple is detected only in specific contexts^12,116^. However, parasitic manipulation could also occur via transcriptionally silent processes or processes that couldn’t be captured by our methodology. The development of a human malaria system in which manipulation is both robust and reproducible is a high priority for characterizing manipulation mechanisms.

Finally, our data provide a set of potential age-grading transcriptional markers of a mosquito’s third week of adult life. This window corresponds to the main life period when mosquitoes contribute to malaria transmission in the field^117^ and when age-discriminant signals among both field and laboratory reared mosquitoes can be detected by spectrometry methods^118,119^. Further exploration could provide relevant targets for the analysis of age structure of field populations.

Our survey of cell type diversity and spatial organization of the brain of the malaria vector provides a foundational effort to understand mechanisms supporting mosquito behavior with the potential of helping identify new strategies to disrupt malaria transmission.

## Methods

### Mosquito rearing

A colony of *An. gambiae* (Kisumu strain^120^) was maintained at 27 °C ± 1 °C and 70% ± 20% relative humidity (RH) and photoperiod of 12 h light/12 h dark in the Vectopole insectarium of IRD in Montpellier, France. Larvae were fed with grinded TetraMin (Tetra) powder and adult mosquitoes fed on 10% honey in water *ad libitum*. A subset of the colony was transferred to Radboudumc (Nijmegen, the Netherlands) for parasite infection, where eggs were collected in plastic mosquito hatching trays in tap water with a drop of Liquifry (Liquifry No1, Interpet). The larvae were fed with TetraMin baby (Tetra). Larvae were maintained at 27 °C ± 1 °C and 80% ± 10% RH and photoperiod of 12 h light/12 h dark and adult mosquitoes at 21 °C ± 1 °C and 80% ± 10% RH and photoperiod of 12 h light/12 h dark, and fed on 5% glucose (D+ Glucose Monohydrate, Sigma 49159) and 0.4% methylparaben (methyl 4-hydroxybenzoate, Sigma H3647-100G) *ad libitum*.

### Single-cell transcriptomics

#### Mosquito sample conditions

In order to capture the diversity of brain cell types along *An. gambiae* females, scRNA-seq experiments included individuals at different stages (i.e., virgin, mated, blood-fed) (Fig. S1A). The two biological replicates originated from two separate egg-laying batches. Starting from an initial pool of mosquitoes at emergence, a set of females was placed in a cage without males (virgin condition). The other female and male mosquitoes were kept together in a cage (mated condition), reaching a 40% of prevalence of mating after 4 days, and a ~ 70% prevalence of mated females after 7 days. We thus selected the latter age for sampling, as it best balanced high mating prevalence and a delayed blood meal after mating, a possible stressor. All cages were fed with 10% honey solution *ad libitum*. On day 6 post-emergence, a subset of the females reared with males were allowed to feed on rabbit blood provided at 37 °C through a glass feeder for 30 min. Engorged mosquitoes were then sorted to constitute the pool of blood-fed females. On the morning of day seven post-emergence, brains of 10 females per condition (virgin, mated, blood-fed) for each replicate were collected. The mosquitoes were anesthetized on ice, and brains were dissected in Dulbecco’s phosphate-buffered saline (DPBS) (Fisher 10217152) on glass slides under a stereomicroscope using steel Dumont #5 dissection forceps. The pharyngeal pump was not removed to avoid subesophageal ganglion damage. For each mosquito, the spermatheca was checked under an optical microscope for the presence/absence of spermatozoids to ensure that the mating state corresponded to the one required for each experimental condition (i.e., virgin = spermatozoids absent; mated/blood-fed = spermatozoids present). Between 8:30 AM and 11 AM, each of three operators dissected brains alternating among mosquitoes belonging to the different conditions. Each experimental condition sample was composed of ten brains, and was then analyzed in a single 10x run.

#### Single-cell dissociation and scRNAseq protocol

We adapted an existing protocol for insect brain cell dissociation^19^. Specifically, pools of 10 dissected brains were placed into 1.5 mL Protein loBind EP tubes (Eppendorf) in 50 μL of ice-cold Sf-900TM III SFM insect cell culture medium (Fisher 12568019). All the tubes in which biological samples were transferred during the protocol had been coated with Bovine serum albumin (BSA)-(Sigma-Aldrich A60003-25G), by incubating them in 1% BSA in sterile Milli-Q water at room temperature (RT) for 30 min, and subsequently emptied and dried under a biological safety cabinet for 30 min. Pools of brains were placed in 125 μL of DPBS (Fisher 10217152) containing 60 mg/mL of collagenase I (Fisher 11500536) and 1.2 mg/mL of dispase I (Sigma D4818-2MG). Samples were kept at 27 °C shaking at 1000 x g for 1 h, every 10 min the samples were pipetted gently ~ 150 times. Dissociation reactions were stopped by addition of 1 mL of DPBS containing 1% of UltraPure BSA (Invitrogen AM2616). The cell suspensions were filtered through a 40 μm mesh-size cell strainer (PluriStrainer 43-50040-51) into a polypropylene falcon tube. Each sample’s cell concentration was measured by staining the cells with an equal volume of Vybrant DyeCycle Green (Fisher 10134622), diluted 1:1000 in DPBS containing 1% UltraPure BSA. Cells were counted in a MilliCell hemocytometer (Sigma MDH-2N1-50PK) with a Zeiss Axio Imager 2 microscope, and inspected with DIC illumination on a glass slide for shape and integrity.

Cells were then pelleted by centrifugation at 800 x g for 5 min at 4 °C. Assuming a loss of ~ 50% of the counted cells, an adequate volume of chilled DPBS with 1% BSA was added to obtain a concentration of ~ 1300 cells/μL. The concentration of each sample was measured and recorded again(between 800 cells/ μL and 1300 cells/ μL). Single-cell suspensions were loaded on a 10x chip with a target recovery of 10,000 cells per sample. Transcriptomes were obtained with a 10x Genomics GemCode Single-Cell Instrument and a Single Cell 3’ Library & Gel Bead Kit v3 (10x Genomics) according to manufacturers’ recommendations (Single Cell 3′ reagent kits v3.1). Amplified cDNA was controlled with High Sensitivity dsDNA quantitation kit (Life technologies Q32851). Libraries were controlled with a Bioanalyzer 2100 (Agilent) and uniquely dual-indexed. Sequencing was multiplexed on a NovaSeq 6000 with paired-end (PE) sequencing (26 and 98 bp) and yielded ~ 1 billion reads per sample.

### Spatial transcriptomics

Seven day old *An. gambiae* virgin females were anesthetized on ice, and whole heads from four individuals per inclusion block (complete with mouthparts, antennae and part of the neck to avoid damaging internal structures) were collected always between 9 AM and 11 AM and aligned with Dumont #5 dissection forceps, then included in 5 × 5 mm plastic molds in optimal cutting temperature compound (OCT) (TFM-C). OCT was solidified by first holding the mold on the surface of isopentane cooled in dry ice, then lowering the whole sample in isopentane for 1 to 2 min. Two inclusion blocks were obtained with this procedure. Samples were stored for at least 12 h at −70 °C before slicing. They were cut axially in a cryostat at −18 °C to obtain 10 μm thick tissue slices, which were collected on glass slides that were stored at RT. Consecutive slices were alternatively used for spatial transcriptomics or stained with hematoxylin and eosin (H&E) to confirm the orientation of the heads and the localization of the anatomical structures. For staining, histological samples were plunged for 1 minute in methanol, followed by 1 min in isopropanol. Glass slides are then laid horizontally and covered for 2 min with hematoxylin, rinsed in tap water and covered for 30 s with eosin. Subsequently, the samples were rinsed again in tap water and dried on a heater at 50 °C. Spatial transcriptomics slices were placed on a Curio Seeker 3 × 3 mm tile (Curio Bioscience SK004), then covered with a 30 µm section of CryoCube provided by the supplier. The tiles were then processed according to the manufacturer’s protocol. Briefly, the tiles were removed from the tile holder and placed into microtubes, and mRNA was captured and hybridized. Reverse transcription was performed in situ, then the tissue was cleared, and indexed beads were dissociated from the tile and resuspended. The second strand synthesis and cDNA amplification were then performed. Libraries were prepared using the Curio bioscience protocol and were individually indexed with the Curio seeker Dual Indexing Primer kit v1 (Curio bioscience K006). Amplified cDNA and library quality was assessed with High Sensitivity dsDNA kit on a Bioanalyzer 2100 (Agilent) and a Qubit (Thermo Fisher). Libraries were pooled and sequenced on a NovaSeq 6000 S4 flow cell using PE150, yielding ~ 650 million reads per library.

### Single brain transcriptomics 

Brains of seven-day-old female mosquitoes that were either virgin (*n* = 9), mated (*n* = 10), or blood-fed and mated (*n* = 10) (as described above for scRNA-seq samples) were dissected (Fig. S1B). For *P. falciparum* infections, NF54 asexual and sexual blood stages were cultured in a semi-automated culture system^121,122^. Six days post-emergence, *An.Anopheles gambiae* females were offered either an infectious bloodmeal or a control, uninfected bloodmeal. Mosquito pots were placed under glass midi-feeders^123^ attached to a circulating water bath set at 39 °C, and allowed to feed in the dark for 10 min. The blood was collected in lithium-heparin tubes (BD 367526), obtained from the national blood bank (Sanquin Nijmegen, the Netherlands). The unfed and the partially fed mosquitoes were removed with a suction device, fully-fed mosquitoes were subsequently maintained at 26 °C ± 1 °C with 80 ± 10% RH and a 12 h/12 h light-dark photoperiod. An egg-laying bowl was placed in the cage and soaked pads with 5% non-sterile glucose solution were placed on top of the cages and changed regularly. Females that were kept until day 22 received a second uninfected bloodmeal 8 days post-infection (dpi) to accelerate oocyst and sporozoite development. Infected mosquitoes were obtained from three different infectious feeding assays, ~ 10 brains (range 8–10) were collected for each timepoint and for each of two biological replicates (Fig. S1C). During brain sample collection, we confirmed the infection status at the level of individual mosquitoes by checking for the presence of (i) oocysts in the midguts at 8 dpi, and (ii) sporozoites in salivary glands of individuals at 16 dpi. The midgut of individuals at 16 dpi were also dissected and intact and ruptured oocysts were counted. Dissected midguts were stained with 0.5% mercurochrome, and oocyst load was assessed by light microscopy. Salivary glands were dissected, ruptured in PBS, and sporozoites were observed by phase contrast microscopy.

Dissected single brains were isolated in 5 μL Sf-900TM III SFM medium (Fisher 11570446) in a well of a 96-well plate kept on ice. RNA was extracted by adding 60 μL volume of a 1.2X ice-cold lysis buffer (120 mM Tris-HCl pH 7.5, 600 mM LiCl, 12 mM EDTA pH 8, 3.6% w/v lithium dodecyl sulfate, 6 mM DTT in nuclease-free water (NFW)) to each well. The samples were left at RT for 1 min under a biological cabinet and subsequently mixed by pipetting up and down ~ 60 times, before storage at −70 °C for at least one night. Pull-down of mRNA in solution was performed with oligo-dT(25) Dynabeads (Invitrogen 61002). Dynabeads were equilibrated in a lysis buffer and 20 µl of beads were added to each well, each well was mixed thoroughly by pipetting and incubated for 15 min at RT while shaking at 1100 rpm. Using a magnet, bead-bound mRNA samples were subsequently washed with two different buffers: first with 10 mM Tris-HCl pH 7.5, 150 mM LiCl, 1 mM EDTA pH 8, LiDS 0.1% w/v in NFW; secondly with 10 mM Tris-HCl pH 7.5, 150 mM LiCl, 1 mM EDTA pH 8 in NFW. The wash buffer was removed and mRNA was eluted by incubation with 10 mM Tris-HCl pH 8 in NFW for 2 min. mRNA was reverse transcribed with an oligo dT30VN (5’-AAGCAGTGGTATCAACGCAGAGTACT_30_VN-3’; Integrated DNA Technologies) priming and a template-switching mechanism, using a SmartScribe kit (Takara 639538), with 5X first-strand buffer and 9.2 U/μL of SmartScribe reverse transcriptase, 2.8 μM LNA-oligonucleotide (5′-AGCAGTGGTATCAACGCAGAGTACATrGrG+G-3′; Qiagen), 0.9 U/μL of Superase Inhibitor (Fisher 10085994), 9 μM DTT (Fisher P2325), 1 M betaine (Sigma B0300-1VL), and 0.01 M MgCl_2_. RT-PCR was carried on with the following program: a 90-minute step at 42 °C, then ten cycles of 2 min at 50 °C and 2 min at 42 °C, with a final step at 72 °C for 15 min. cDNA was then amplified using a KAPA HiFi HS mix (Merck 3736549) and a primer (5’-AAGCAGTGGTATCAACGCAGAGT-3’) with a PCR program with an initial incubation step at 98 °C for 3 min, followed 14 PCR cycles (98 °C for 20 s, 67 °C for 15 s, 72 °C for 6 min) and a final step at 72 °C for 15 min. After a first purification with Agencourt Ampure XP beads (0.8:1), a low-input Nextera protocol including a step for sample dual indexing was used to index every single brain with a specific pair of primers of the Nextera DNA Unique Dual Indexes Set A and C (Illumina 20027213, 20027215). The libraries were again purified with Agencourt Ampure XP beads (0.8:1) and their quality was assessed with High Sensitivity dsDNA kit on a Bioanalyzer 2100 (Agilent) and a Qubit (Thermo Fisher). The libraries were pooled and sequenced on an Illumina HiSeq 4000 with PE150 with ~ 1 million reads per library.

### Confocal microscopy

Female *An. gambiae* were placed in 70% ethanol for five seconds before brain dissection in ice-cold Schneider’s Insect Medium (S2). Brains were fixed in 2% paraformaldehyde solution in S2 at room temperature for 60 min. Fixed brains were then washed 10 min in PBS containing 0.5% Triton-X (PBST), this step was repeated three times. Brains were then incubated in a blocking solution (5% goat serum (Jackson Immunoresearch) in PBST) for 90 min at room temperature. Brains were then incubated with a primary antibody, α-Bruchpilot, nc82, (1:30, mouse, DSHB) in PBST containing 5% goat serum for 72 h at 4 ˚C. Brains were washed for 10 min four times in PBST, and subsequently incubated with a secondary antibody, (Alexa Fluor 547-conjugated goat α-mouse; 1:400; Jackson Immunoresearch) overnight at 4˚C. After four 10 min washes in PBS, brains were mounted in Vectashield (Vector Labs) on a glass slide. *An. gambiae* brains were imaged with a Leica SP8-UV confocal microscope. Image resolution was 1024 × 512 with 0.5 µm step size and a frame average of 3 with a 20x objective. All images were analyzed using Fiji^124^.

### RNA fluorescence in situ hybridization

Sections of whole heads from seven days old *An. gambiae* of virgin females were prepared as above, by inclusion in OCT (TBS) and collection of 10 µm tissue slices. Mosquito heads were included in OCT in groups of five per mold, to ensure enough material was available for sample preparation. Seventy-six glass slides with four to six block slices were prepared from seven different inclusion blocks, and twenty-four glass slides obtained from five different inclusion blocks were used for ISH preliminary tests and main experiments. Glass slides pre-treatment and probe hybridization were carried out according to the ACD manufacturer protocol (UM 323100 Rev B). Samples were fixed for 30 min in formaldehyde 4% and subsequently dehydrated with increasing ethanol concentrations. Samples were permeabilized with RNAscope protease IV for 30 min. Staining was performed with an RNAscope Multiplex Fluorescent Reagent Kit v2. Hybridized probes consisted of different combinations of three of these probes: (i) ACD Aga-LOC1270460-O1 (ACD 1792161-C1) targeting *elav-like*; (ii) Aga-LOC1274238-O1 (ACD 1792148-C2) targeting *repo*; (iii) Aga-Tret1-1-O1 (ACD 1792131-C3) targeting *tret1*; (iv) Aga-Col4a1-O1 (ACD 1792151-C2) targeting *col4a*; (v) Aga-Fer2 (ACD 1800291-C3) targeting *AGAP003739*; (vi) Aga-Adcy8 (ACD 1800531-C2) targeting *adenylate cyclase 8*; and (vii) Aga-LOC1272412 (ACD 1800521-C3), targeting *D1-like dopamine R2*. C1, C2, and C3 in the probe codes represent three different fluorescence detection channels associated with three different fluorophores. Chosen fluorophores were Opal620 (Akoya FP1495001KT) for C1, Opal540 (Akoya FP1494001KT) for C2, and Opal650 (Akoya FP1496001KT) for C3. Opal fluorophores were reconstituted in DMSO according to manufacturer’s guidelines and diluted at a ratio of 1:1,500 in TSA buffer (ACD 322810). Nuclei were stained with DAPI provided in the RNAscope kit. Glass slides were mounted with Prolong Gold Antifade Mountant (Thermo Fisher, P10144) for 30 min at RT. Cryo-slices were kept at −80 °C or on dry ice until use. Presence of non-specific fluorescence was checked with an RNAscope negative control probe (ACD 320871), fluorophores alone, and with single probe labeling. Non-specific fluorescence associated with the presence of opal fluorophores was detected in association with ommatidia and keratin in all negative control conditions and was therefore ignored during the interpretation of the results. Imaging was performed on a Zeiss LSM 780 confocal microscope, with X10 (EC PlnN 10X/0.3 DIC I) and X63 (Pln Apo 63X/1.4 oil DIC III) objectives, and ZEN 3.11 software. Fluorophores assigned to the different channels were excited with lasers of wavelength 405 nm (30 mW) (DAPI); 512 nm (25 mW) (C2); 561 nm (20 mW) (C1); 633 nm (5 mW) (C3). The context of the whole head slice was captured for each image, and insets were subsequently captured at higher resolution. The images shown here are representative of the results obtained from each probe set and chosen among 115 relevant images. All images were analyzed using Fiji^124^.

### Bioinformatic data processing and analysis

#### Single cell transcriptomics

Data analysis was carried out with R (v4.2.2)^125^ and RStudio environment (v2023.06.1)^126^. Fastq files were demultiplexed and mapped to the *An. gambiae* PEST genome (AgamP4.14) with CellRanger (v7.0.0)^127^ and with an annotation file obtained from VectorBase (v57). Low quality transcriptomes were excluded if they had fewer than 1000 unique transcripts, less than 800 genes and more than 20% mitochondrial genes; genes were filtered out if they were present in less than five cells. Background noise from ambient RNA molecules and random barcode swapping was removed with CellBender^128^ (v3.0). Single-cell analysis was conducted with Seurat (v5.0.1)^129^. Doublets were identified and removed with DoubletFinder^130^ (v2.0.4). Transcriptomes were normalized and integrated using SCTransform. Clustering analysis was performed in Seurat kNN method based on Jaccard’s index with a resolution of 0.7 based on 40 principal components and on a *clustree* (v 0.5.1) branching, until cluster assignments became unstable across resolutions, to avoid overclustering. Positive cluster marker genes were identified by comparing all the clusters against each other (Seurat *FindAllMarkers* function with a MAST test^131^ and a base-2 logarithmic fold change (LFC) threshold of 0.25 and a minimum fraction of 25% of cells in either of the two populations expressing the gene). The same approach was used to assess DEGs among conditions for each cluster. Any significance value inferior to RStudio minimal representable floating number (<2.225074E-308) resulted as zeros in the analysis outputs. Cell type mapping was conducted with SAMap (v1.0.15)^132^ against annotated references of *D. melanogaster*^19–21^, *Ae. aegypti*^7^, *A. mellifera*^8^ and *M. paraonis*^5^. For the analysis, the chosen reference datasets were subset to keep only suitable sample types for an unbiased cell-type assignation as follows: only female flies were kept from the scRNA-seq data from Davie et al.^19^, sated flies data were kept from Park et al.^21^, only adult data n. 1 to n. 8 were kept from Özel et al.^20^, and we downsampled the number of cells for each cell type in the dataset from Li et al.^5^ (50 %). We did not select for specific adult life cycle stages (e.g., based on *D. melanogaster* age or on the choice between workers or queens in social insects) because we surmised that they could not introduce biases in our analysis. The coding sequences used were of *An. gambiae* obtained from VectorBase (v57), of *Ae. aegypti* obtained from VectorBase (v58) of *D. melanogaster* obtained from FlyBase (r6.55)^133^, of *A. mellifera* obtained from NCBI (based on genome assembly Amel_HAv3.1), of *M. pharaonis* (based on the genome assembly M.pharaonis_V2.0 and downloaded from the Li and colleagues FigShare repository^134^), trying to match the ones used to annotate the reference sc/snRNA-seq datasets. The reciprocal BLAST results were processed to keep only gene IDs as identifiers to match the reference datasets. SAMap pipeline was performed as recommended^6^. We empirically set a threshold of 0.4 of SAMap alignment score for manual cell type assignments to Ag clusters.

Gene orthology was based on orthogroups obtained on OrthoMCL^135^ release 6.21 (7 May 2024) from *D. melanogaster* whole transcriptome and the list of *An. gambiae* genes expressed in our data.

#### Spatial cell transcriptomics

Demultiplexed fastq files of each bloc were processed using Curio Seeker Pipeline (v2.0.0) within the Nextflow environment (v24.10.3, build 5933). STAR (c2.6.1 d) mapping against the *An. gambiae* PEST genome (AgamP4.14) was conducted by following the pipeline instructions for custom reference genomes using the generate_seeker_reference.sh and pygtftk (v1.6.2) to add missing “gene_names” to the GTF file obtained from VectorBase (v56). Downstream analysis of the data was carried out with RStudio environment (v2024.12.1) using R (v4.4.3). Data were cleaned from unused spots (selecting only spots > 100 UMIs, > 50 genes, <40% mitochondrial mapping reads), combined, and analyzed using Seurat^129^ (v5.3.0). Normalization and transformation were performed with SCTransform (v0.4.1) regressing out bloc and mitochondrial mapping rates. UMAP dimension reduction was performed on 40 principal components, and spots were clustered with the Seurat kNN method based on Jaccard’s index with a resolution of 0.5, based on a 50-dimensional PCA. Cluster marker genes were identified by comparing all the clusters against each other (Seurat FindAllMarkers function with a MAST test^131^ and a LFC threshold = 0.5 and a minimum fraction of 25% of spots in either of the two populations expressing the gene). Annotation of spots to mushroom bodies, MIP cells and monoaminergic/peptidergic neurons was performed by combining marker expression and reclustering of spots assigned to spatial cluster Sp2. Differentially expressed genes were obtained from the renormalized data of all spots of this cluster by contrasting assigned spots to all other spots within this cluster using the MAST algorithm as implemented in Seurat using the same parameters as before. Functional enrichment analysis of marker genes was performed using richr (v0.0.37). Deconvolution of spots against the single-cell dataset was performed using spacexr (v2.2.1). Assignment of spots to single brains was performed visually using the fhs function of GatePoints (v0.1.5), and spot coordinates for each brain were rotated around the center using the rotator function of ArtPack (v0.1.0) for representation. Five brain slices were selected for further analysis based on their integrity. Cluster distance analysis of these 5 slices was performed on non-rotated coordinates for every single brain separately by calculating the mean minimal Euclidean distance of each cluster to all other clusters. Non-directional distances were then calculated by taking the mean of each combination. Expression by distance analysis within eye regions was performed by calculating the Euclidean distance of each spot within the selected area to the selected point of origin. Gene expression by distance was modeled by fitting a natural cubic spline (splines package, 10 degrees of freedom) per eye area for all genes in the dataset. To identify genes showing specific spatial expression patterns they were subsequently filtered for a minimum expression range of 1.5 normalized UMIs and a R^2^ > 0.1 of the natural cubic spline fit. Cluster abundances within the eye section were estimated by the frequency of assigned spots to each cluster within a given distance bin of 10 pixels. Distributions were modeled by applying a natural cubic spline (10 degrees of freedom) fit over the binned occurrences of clusters.

#### Single-brain transcriptomics

Single brain bulk RNA-seq data were aligned to the *An. gambiae* PEST genome (AgamP4.14) with hisat2^136^ (v2.2.1) and read count performed with HTSeq^137^ (v2.0) with an annotation file obtained from VectorBase (v56). Downstream analysis of the data was carried out with RStudio environment (v2023.06.1) using R (v4.2.2). For life stage analysis, one virgin condition sample was filtered out as it had a read count that indicated a failed amplification (21,961 reads vs 12,748,569 reads on average in other samples). Data from all samples were pooled, normalized and fit to a linear model, and dispersion was estimated with the DESeq function of DESeq2^138^ (v1.36.0). Differential expression analysis (DEA) was carried out using LFC values shrinkage with the *apeglm* method^139^. Changes in gene expression were considered significant if associated with a Benjamini-Hochberg (FDR) value < 0.05 and an LFC > 0.25.

For infection data, one uninfected sample at day 16 was removed as outlier (9,055 reads vs 5,970,229 reads on average in other samples). For the infection data, the DEA was conducted with different permutations, for instance, with DESeq objects containing: (i) individual uninfected vs infected replicate, (ii) individual day 14 vs day 22 replicate, (iii) pooled samples by either infection status or age. The reference state was set for either infected samples or day 14 samples, depending on the test performed.

For all comparisons, data were normalized and fit to a linear model, and dispersion was estimated with the DESeq function of DESeq2^138^ (v1.36.0). DEA was carried out using LFC shrinkage both with the *apeglm* method^139^ and with the *ashr* method^140^ especially for more complex designs (i.e., combining infection and age), the method used is specified in the figure legend. Results of the two analyses were contrasted to both obtain highly reliable LFC estimates (*apeglm*) and to avoid model over-parameterization (*ashr*). Reasoning that infection could have subtle effects on brain transcriptomes, and that applying DESeq2 shrinkage methods corrects for low-count value dispersion artifacts, the LFC threshold was set to zero, and results having an FDR value < 0.05 were considered significant.

Gene function information was retrieved from gene annotation present in VectorBase database^141^. Immune gene family enrichment in aging was performed based on categorized gene families in the *An. gambiae* PEST genome annotation GCA_000005575.2, AgamP4.14 (VectorBase Release 68 in May 2025)^142^. To ensure only annotated members were counted, the genes belonging to each immune family were counted based on gene names, and not on gene product description, which could have been automatically assigned without manual curation. The genes considered to be DE for the analysis had a |log_2_FC | > 0.25. Hypergeometric p-values were calculated using the upper-tail (right-tail) probability P(X ≥ a). The FDR was adjusted across all known immune families present in the *An. gambiae* PEST genome using the Benjamini-Hochberg method.

### Statistics and reproducibility

H&E stainings on histological slices were repeated tens of times with similar results during both preliminary tests and main experiments. ISH stainings for each probe set was evaluated during both preliminary tests and main experiments over at least 20 different head slices each coming from a different mosquito head.

### Reporting summary

Further information on research design is available in the Nature Portfolio Reporting Summary linked to this article.

## Supplementary information

Supplementary Information

Peer Review file

Description of Additional Supplementary Files

Supplementary Data 1

Supplementary Data 2

Supplementary Data 3

Supplementary Data 4

Supplementary Data 5

Supplementary Data 6

Supplementary Data 7

Supplementary Data 8

Reporting Summary

## Source data

Source Data

## Data Availability

All raw sequencing data in this study have been deposited in the European Nucleotide Archive at European Molecular Biology Laboratory European Bioinformatics Institute (www.ebi.ac.uk/ena/) under accession number ERP173390. Additional data including source data is accessible on Zenodo (https://doi.org/10.5281/zenodo.21492585). All analysis code is available via Github (https://github.com/Parasitedevbio/Agambiae_Brain_Atlas.git - doi: 10.5281/zenodo.21487293). Source data are provided as a Source Data file. The datasets presented in the study can also be visualized and explored on https://vectorbase.org/ and https://cellxgene.cziscience.com/ associated with the publication reference. Source data are provided with this paper.

## Code availability

Analysis code is accessible via Github (https://github.com/Parasitedevbio/Agambiae_Brain_Atlas.git).
