# Supplementary material for: The brain of the female Anopheles gambiae mosquito at spatial and single-cell resolution
Source: Nat Commun. 2026 Sep 17;17:10940. doi: 10.1038/s41467-026-76839-z (PMC13680466; doi:10.1038/s41467-026-76839-z)
Supplement: Supplementary file 1 [file 41467_2026_76839_MOESM12_ESM.pdf]

Corresponding author(s): Arthur M. Talman, Anna Cohuet, Irene Z. Rossi

Last updated by author(s): 22/07/2026

Reporting Summary

Nature Portfolio wishes to improve the reproducibility of the work that we publish. This form provides structure for consistency and transparency in reporting. For further information on Nature Portfolio policies, see our Editorial Policies and the Editorial Policy Checklist.

Statistics

For all statistical analyses, confirm that the following items are present in the figure legend, table legend, main text, or Methods section.

n/a	Confirmed	
☐	☒	The exact sample size (*n*) for each experimental group/condition, given as a discrete number and unit of measurement
☐	☒	A statement on whether measurements were taken from distinct samples or whether the same sample was measured repeatedly
☐	☒	The statistical test(s) used AND whether they are one- or two-sided<br>*Only common tests should be described solely by name; describe more complex techniques in the Methods section.*
☐	☒	A description of all covariates tested
☐	☒	A description of any assumptions or corrections, such as tests of normality and adjustment for multiple comparisons
☐	☒	A full description of the statistical parameters including central tendency (e.g. means) or other basic estimates (e.g. regression coefficient) AND variation (e.g. standard deviation) or associated estimates of uncertainty (e.g. confidence intervals)
☐	☒	For null hypothesis testing, the test statistic (e.g. *F*, *t*, *r*) with confidence intervals, effect sizes, degrees of freedom and *P* value noted<br>*Give P values as exact values whenever suitable.*
☒	☐	For Bayesian analysis, information on the choice of priors and Markov chain Monte Carlo settings
☐	☒	For hierarchical and complex designs, identification of the appropriate level for tests and full reporting of outcomes
☒	☐	Estimates of effect sizes (e.g. Cohen's *d*, Pearson's *r*), indicating how they were calculated

Our web collection on statistics for biologists contains articles on many of the points above.

Software and code

Policy information about availability of computer code

Data collection	n/a
Data analysis	R (v4.2.2)<br>RStudio (v2023.06.1)<br>CellRanger (v 7.0.0)<br>CellBender (117) (v3.0)<br>SAMap (v1.0.15)<br>Curioseekeer Pipeline (v2.0.0)<br>Nextflow (v24.10.3, build 5933)<br>Star (c2.6.1d)<br>hisat2 (v2.2.1)<br>HTSeq (v2.0)

For manuscripts utilizing custom algorithms or software that are central to the research but not yet described in published literature, software must be made available to editors and reviewers. We strongly encourage code deposition in a community repository (e.g. GitHub). See the Nature Portfolio guidelines for submitting code & software for further information.

Data

Policy information about availability of data

All manuscripts must include a data availability statement. This statement should provide the following information, where applicable:

- Accession codes, unique identifiers, or web links for publicly available datasets
- A description of any restrictions on data availability
- For clinical datasets or third party data, please ensure that the statement adheres to our policy

All raw sequencing data in this study have been deposited in the European Nucleotide Archive at European Molecular Biology Laboratory European Bioinformatics Institute (www.ebi.ac.uk/ena/) under accession number ERP173390. Additional data including source data is accessible on Zenodo (https://doi.org/10.5281/zenodo.21492585).
The datasets presented in the study can also be visualized and explored on https://vectorbase.org/ and https://cellxgene.cziscience.com/ associated with the publication reference.

Research involving human participants, their data, or biological material

Policy information about studies with human participants or human data. See also policy information about sex, gender (identity/presentation), and sexual orientation and race, ethnicity and racism.

Reporting on sex and gender	n/a
Reporting on race, ethnicity, or other socially relevant groupings	n/a
Population characteristics	n/a
Recruitment	n/a
Ethics oversight	n/a

Note that full information on the approval of the study protocol must also be provided in the manuscript.

Field-specific reporting

Please select the one below that is the best fit for your research. If you are not sure, read the appropriate sections before making your selection.

[x] Life sciences [ ] Behavioural & social sciences [ ] Ecological, evolutionary & environmental sciences

For a reference copy of the document with all sections, see nature.com/documents/nr-reporting-summary-flat.pdf

Life sciences study design

All studies must disclose on these points even when the disclosure is negative.

Sample size	Each condition in the single cell data arose from 20 different mosquitoes sequenced on two different chromium assays. A preliminary dataset of 2k cells was also obtained with the same protocol, yielding similar results in terms of gene expression and markers. For single brain bulk RNAseq, there were single brains from 10 individual females for each condition (virgin, mated and bloodfed; oocyst-infected, sporozoite-infected and controls). The same bulk RNA-seq protocol was used on independent preliminary tests before obtaining the libraries for the life stages and infection conditions, and results were obtained at three years distance, independently. For all these experiments we performed no sample size calculation, and sample sizes of 10 or 20 biological replicates per condition were selected to exceed the minimal commonly used exploratory threshold and to provide substantially improved precision and robustness for estimating effect sizes and variability. The spatial transcriptomics data analyzed 8 brain independent head section, analyzed on two arrays. No replication was performed for the spatial transcriptomics experiments. FISH experiments were performed on no less than 3 independent head sections. Representative images are shown. H&E staining on histological slices were repeated more than ten times with similar results during both preliminary tests and main experiments. ISH staining for each probe set was evaluated during both preliminary tests and main experiments over at least 20 different head slices each coming from a different mosquito head.
Data exclusions	For life stage analysis, one virgin condition sample was filtered out as it had a read count that indicated a failed amplification (21,961 reads vs 12,748,569 reads on average in other samples).<br>For infection data, one uninfected sample was removed as outlier (9,055 reads vs 5,970,229 reads on average in other samples).
Replication	ScRNA-seq experiment and bulk RNA-seq experiment on infected mosquitoes arose from no less than two mosquito batches. Plasmodium-infected mosquitoes were from 2 independent batches of parasites cultures.
Randomization	Allocation of samples to experimental groups was random.
Blinding	Blinding was not performed, as it was necessary to easily keep track of sample identity for a fast sample collection and processing to preserve sample quality.

Reporting for specific materials, systems and methods

We require information from authors about some types of materials, experimental systems and methods used in many studies. Here, indicate whether each material, system or method listed is relevant to your study. If you are not sure if a list item applies to your research, read the appropriate section before selecting a response.

Materials & experimental systems

n/a	Involved in the study	
☐	☒	Antibodies
☐	☒	Eukaryotic cell lines
☒	☐	Palaeontology and archaeology
☐	☒	Animals and other organisms
☒	☐	Clinical data
☒	☐	Dual use research of concern
☒	☐	Plants

Methods

n/a	Involved in the study	
☒	☐	ChIP-seq
☒	☐	Flow cytometry
☒	☐	MRI-based neuroimaging

Antibodies

Antibodies used	- nc82, DSHB<br>- Alexa Fluor 547-conjugated goat α-mouse, Jackson Immunoresearch
Validation	nc82, confirmed species reactivity: Aedes aegypti, Drosophila, Mosquito.

Eukaryotic cell lines

Policy information about cell lines and Sex and Gender in Research

Cell line source(s)	Plasmodium falciparum, NF54.
Authentication	none of the cell lines used were authenticated
Mycoplasma contamination	not present
Commonly misidentified lines (See ICLAC register)	n/a

Animals and other research organisms

Policy information about studies involving animals; ARRIVE guidelines recommended for reporting animal research, and Sex and Gender in Research

Laboratory animals	Anopheles gambiae, kisumu
Wild animals	n/a
Reporting on sex	Only females mosquitoes are included the study, since they are the ones responsible for malaria transmission
Field-collected samples	n/a
Ethics oversight	Authorization was obtained for use of rabbit blood to feed the mosquitoes (APAFIS #50999-2024090823306605 v4)

Note that full information on the approval of the study protocol must also be provided in the manuscript.

Plants

Seed stocks	n/a
Novel plant genotypes	n/a
Authentication	n/a
