# Supplementary material for: The brain of the female Anopheles gambiae mosquito at spatial and single-cell resolution
Source: Nat Commun. 2026 Sep 17;17:10940. doi: 10.1038/s41467-026-76839-z (PMC13680466; doi:10.1038/s41467-026-76839-z)
Supplement: Supplementary file 2 [file 41467_2026_76839_MOESM1_ESM.pdf]

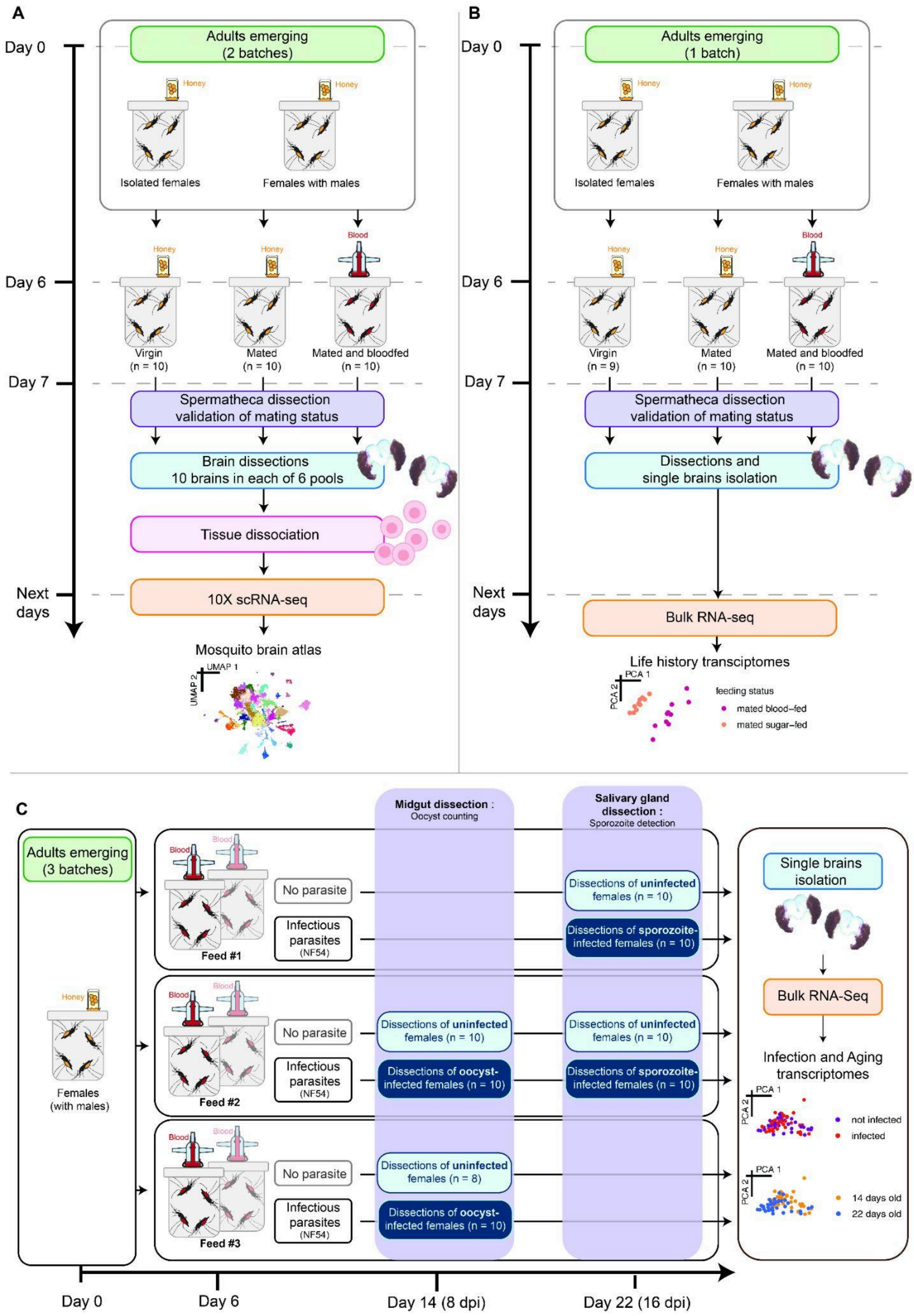

Figure S1. Experimental design of brain transcriptomics experiments. (A) Experimental workflow for sc-RNA-seq data. One batch of mosquitoes corresponds to adults emerging from a single egg-laying batch. One

pool is composed of n=10 brains obtained from mosquitoes that were either all virgin, all mated, or all mated and blood-fed. Adult mosquitoes from each of the two batches were separated on the morning of day 1 post-emergence into cages with virgin females only, or with females and males, allowing mating. At day 6 post-adult emergence a subset of the females that were allowed to mate were offered a bloodmeal, and the engorged ones later isolated. Eventually, scRNA-seq samples were obtained and processed. **(B)** Experimental workflow for single brain bulk RNA-seq of life cycle stages. Mosquitoes from one batch were separated regarding their status virgin, mated, mated and bloodfed as described for panel A. Eventually, whole brains were isolated and samples processed for bulk RNA-seq. **(C)** Experimental workflow for single brain bulk RNA-seq of *P. falciparum* infection stages and mosquito age. For each of the 3 batches, females were kept with males and on day 6 post-adult emergence, they were fed with blood with or without infectious *P. falciparum* gametocytes. Eventually, whole brains were isolated at either 8 or 16 days post-bloodmeal, and samples processed for bulk RNA-seq.

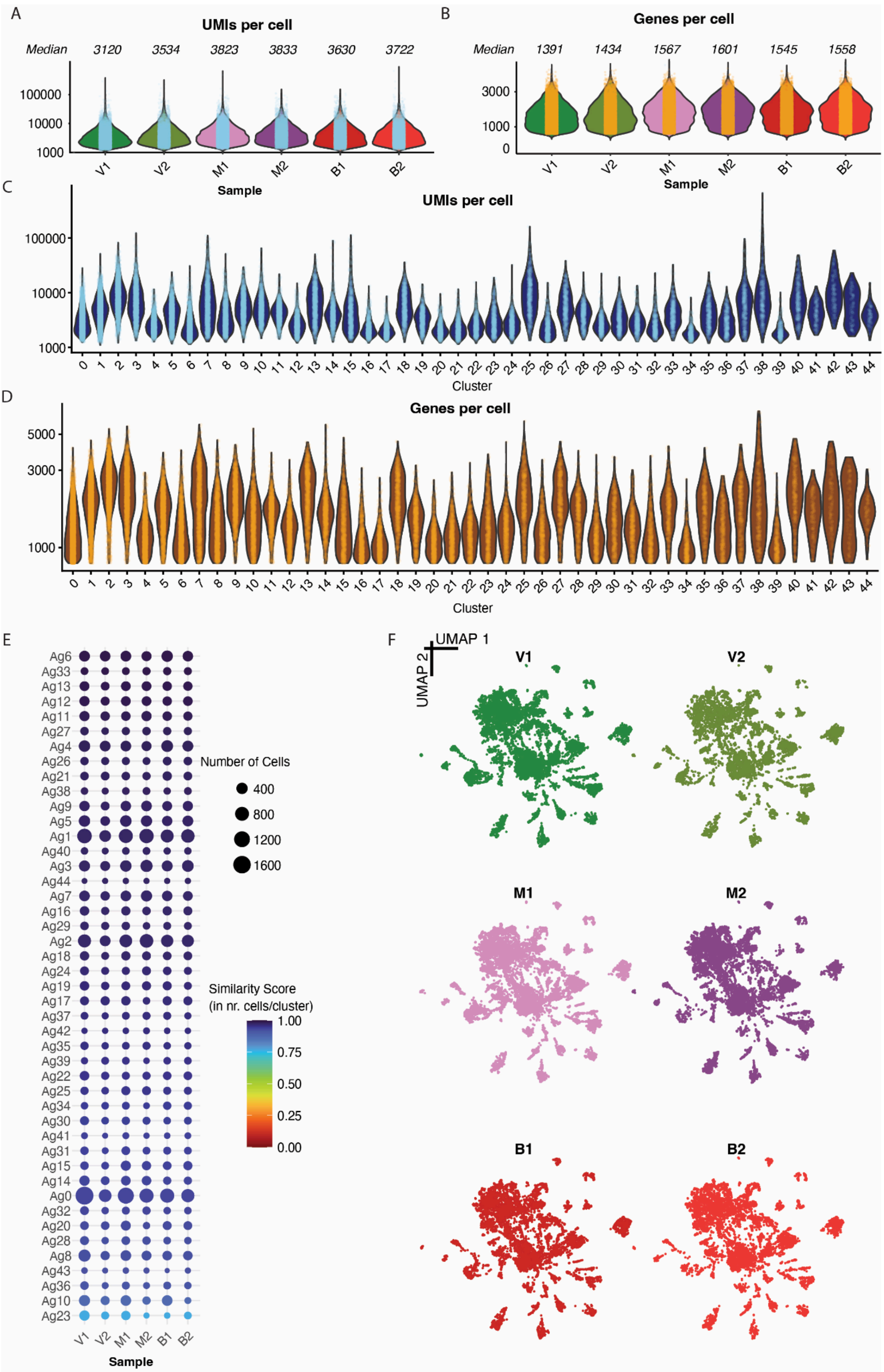
A
UMIs per cell
Median 3120 3534 3823 3833 3630 3722
100000
10000
1000
V1 V2 M1 M2 B1 B2
Sample
B
Genes per cell
Median 1391 1434 1567 1601 1545 1558
3000
1000
0
V1 V2 M1 M2 B1 B2
Sample
C
UMIs per cell
100000
10000
1000
Cluster
D
Genes per cell
5000
3000
1000
Cluster
E
Number of Cells
400
800
1200
1600
Similarity Score
(in nr. cells/cluster)
1.00
0.75
0.50
0.25
0.00
Ag6 Ag33 Ag13 Ag12 Ag11 Ag27 Ag4 Ag26 Ag21 Ag38 Ag9 Ag5 Ag1 Ag40 Ag3 Ag44 Ag7 Ag16 Ag29 Ag2 Ag18 Ag24 Ag19 Ag17 Ag37 Ag42 Ag35 Ag39 Ag22 Ag25 Ag34 Ag30 Ag41 Ag31 Ag15 Ag14 Ag0 Ag32 Ag20 Ag28 Ag8 Ag43 Ag36 Ag10 Ag23
V1 V2 M1 M2 B1 B2
Sample
F
UMAP 1
UMAP 2
V1
V2
M1
M2
B1
B2

Figure S2. Quality control of the brain single cell atlas. (A) Violin plot of log-scale UMI counts in each replicate after QC. **(B)** Violin plot of log-scale gene counts in the scRNA-seq replicates after QC. The median count metric is given for each replicate. **(C)** Violin plot of UMI counts in each cluster after QC. **(D)** Violin plot of gene counts in each cluster after QC. **(E)** Dotplot showing degree of similarity (cosine similarity) in the number of cells from each replicate and making up a cluster for all of the 45 clusters. Clusters are ordered from the one having the most similar number of cells per experimental replicate (top) to the one that showed the greater differences among sample replicates (bottom). **(F)** UMAPs showing the distribution of cells in the different sample replicates. Virgin sugar-fed replicate #1: V1; virgin sugar-fed replicate #2: V2; mated sugar-fed replicate #1: M1; mated sugar-fed replicate #2: M2; mated blood-fed replicate #1: B1; mated blood-fed replicate #2: B2.

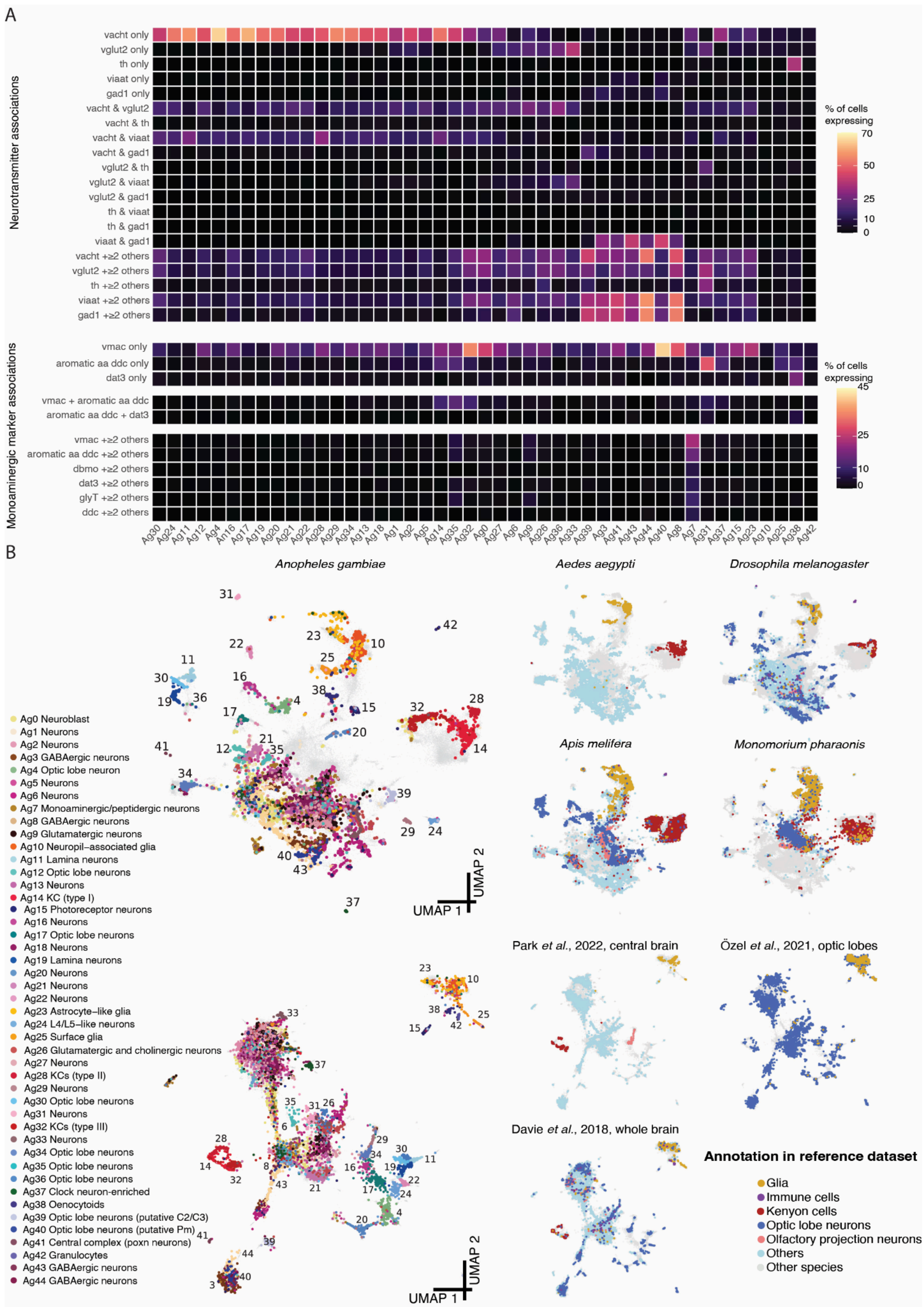

Figure S3. Characterizing *An. gambiae* brain cell types through neurotransmitter type assignment and multispecies cell type homology. (A) Neurotransmitter prevalence in Ag clusters, shown as percentage of cells expressing the marker genes (y axis) with SCTransform normalized counts > 0.5 in each cluster. In the bottom heatmap, rows associated with marker combinations displaying a percentage of expressing cells < 5 % in all clusters are not shown. **(B)** Clustering of cells belonging to the multispecies panels for cross-species homology

assignment. A sampling of 25% of cells from each cluster was applied for representation purposes. In the top panels the UMAPs resulting from the stitched manifolds of our atlas with *Ae. aegypti* [7], *D. melanogaster* [19], *A. mellifera* [8], and *M. pharaonis* [5] are shown and cells highlighted based major type classification. Similarly, the bottom panels represent the stitched manifold of our atlas with *D. melanogaster* central brain [21], optic lobes [20] and whole brain [19,20].

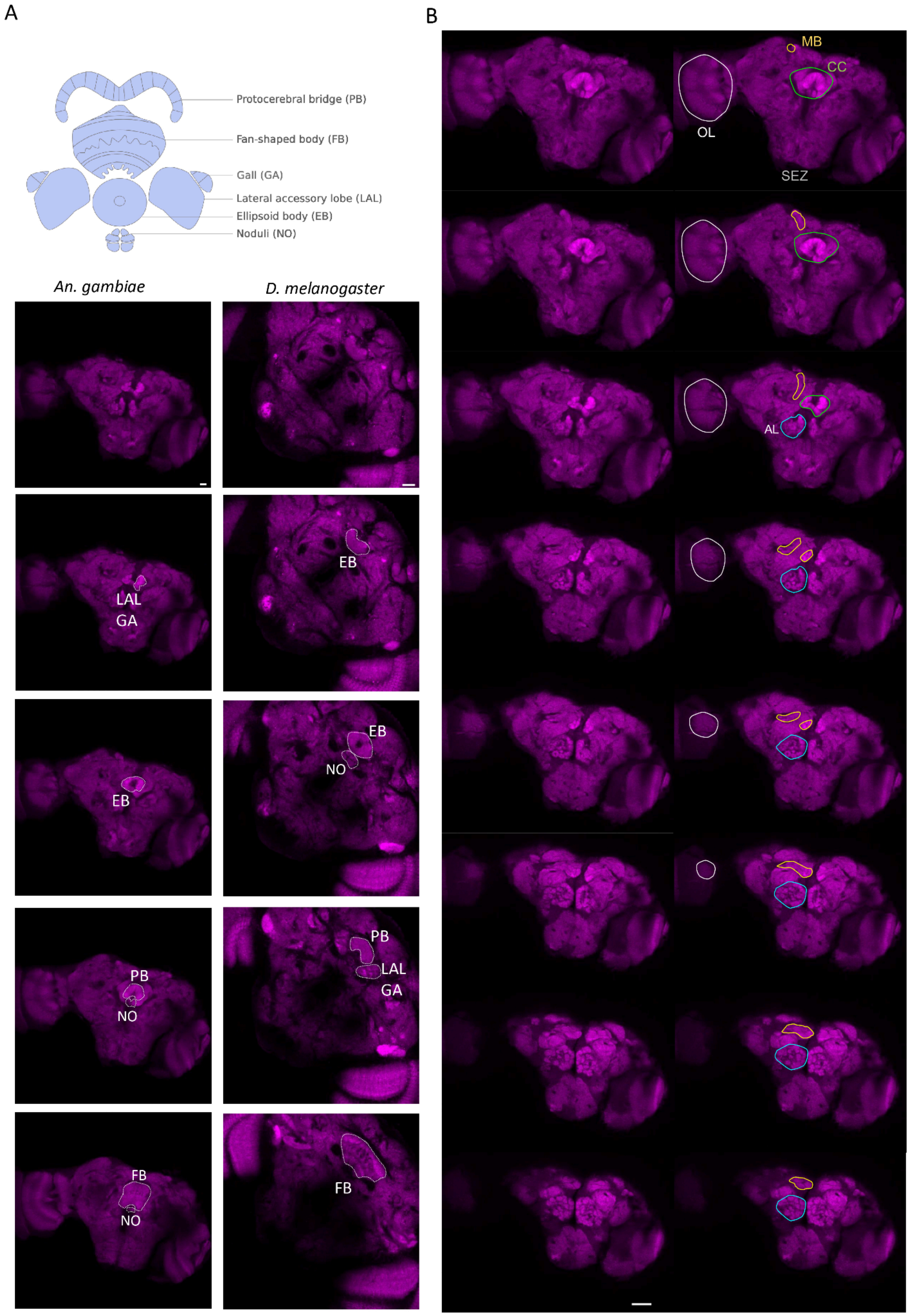

Figure S4. Anatomical structures in the *An. gambiae* brain. Representative confocal microscopy image showing Z-stacks of female *An. gambiae* and *D. melanogaster* brains stained with an anti-Bruchpilot antibody, a component of the presynaptic active zone. (A) Comparative structures in the central complex highlighted in the

brains of both species alongside a schematic, adapted from [143]. (**B**) Other structures observed in the *An. gambiae* female brain, the images are displayed bare (left) or with annotation of identifiable structures (right). SEZ: subesophageal zone; MB: mushroom body; CC: central complex; OL: optic lobe; AL: antennal lobe. Scale bar 50 μm.

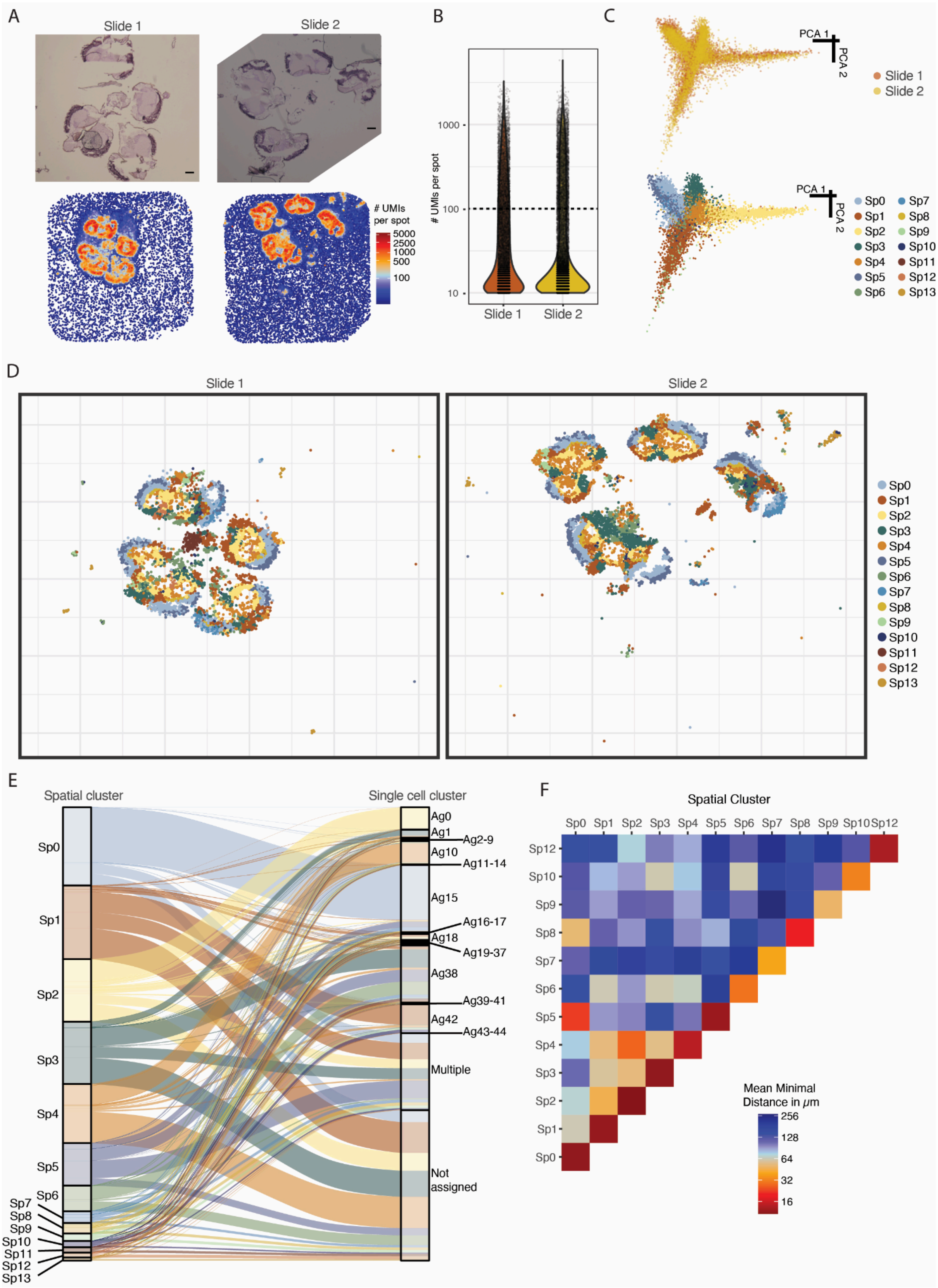

Figure S5. A spatially-resolved transcriptomic dataset of the female *An. gambiae* head. (A) H&E stained sections immediately adjacent sections of the two blocks (top panels) used in spatial transcriptomics (bottom panels) depicting the number of UMIs per spot in eight individual brains. **(B)** Distribution of UMIs per spot in both blocks, the cut-off was set to no less than 100 UMIs per spot. **(C)** PCA of the 9,914 high quality spatially-resolved transcriptomes retained in the final dataset, highlighting their block of origin (top panel) and the spatial cluster

they belong to (bottom panel). **(D)** Spatial representation of spatial clusters for each block. **(E)** Riverplot of the deconvolution of spatial clusters with the single cell clusters. **(F)** Mean minimal non directional distances between different spatially resolved clusters within the same brains, highlighting the relative proximity of clusters in space.

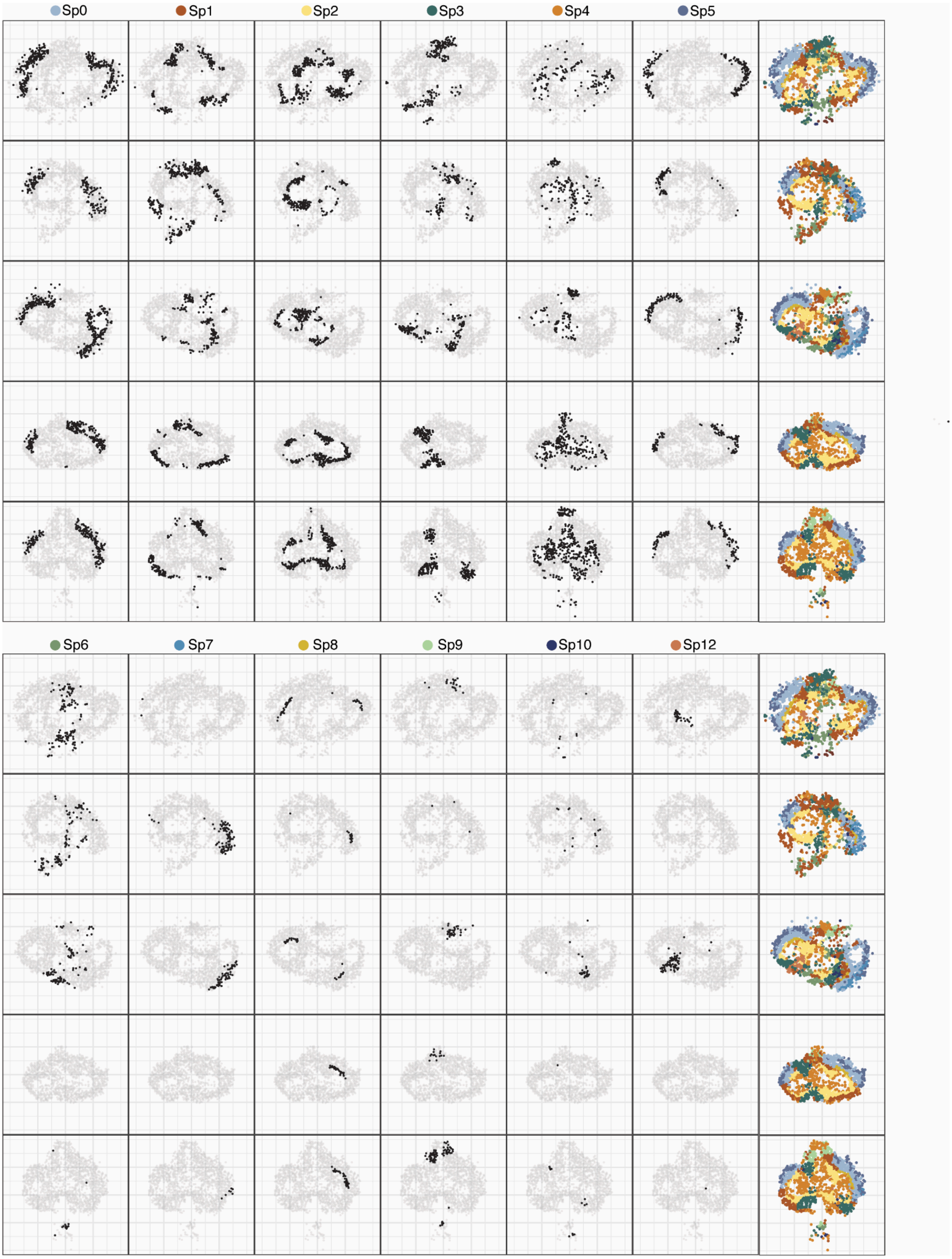

Figure S6. Spatial cluster distribution within the five high quality heads. Sp11 and Sp13 are absent because they were not represented within the head space.

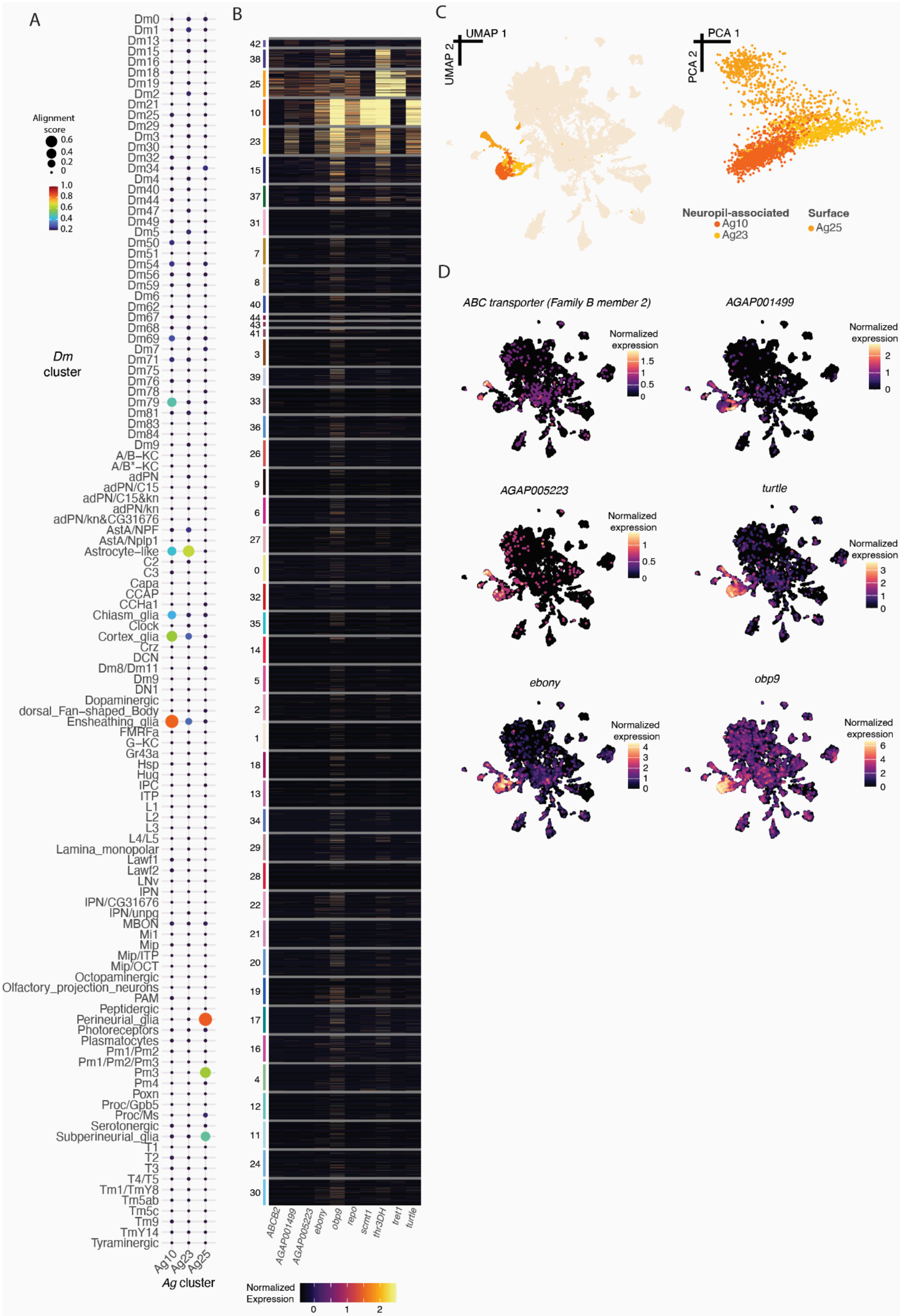

Figure S7. Characterization of glial cell diversity in the female *An. gambiae* brain. (A) *D. melanogaster* cell type assignment (SAMap) to Ag clusters compared to Davie *et al*. 2018 [19]. Dot size and color code correspond to the alignment score value for each Dm cell type - Ag cluster pair. **(B)** Heatmap of expression data (normalized

with SCTransform, scaled with base R scale function) downsampling a maximum of 500 cells per Ag cluster (y-axis). **(C)** Glia clusters identification in the atlas (UMAP, left panel) and their PCA projection after subsetting and application of SCTransform (right panel). **(D)** UMAPs showing expression distribution of cell markers among cell types, expressing cells in foreground.

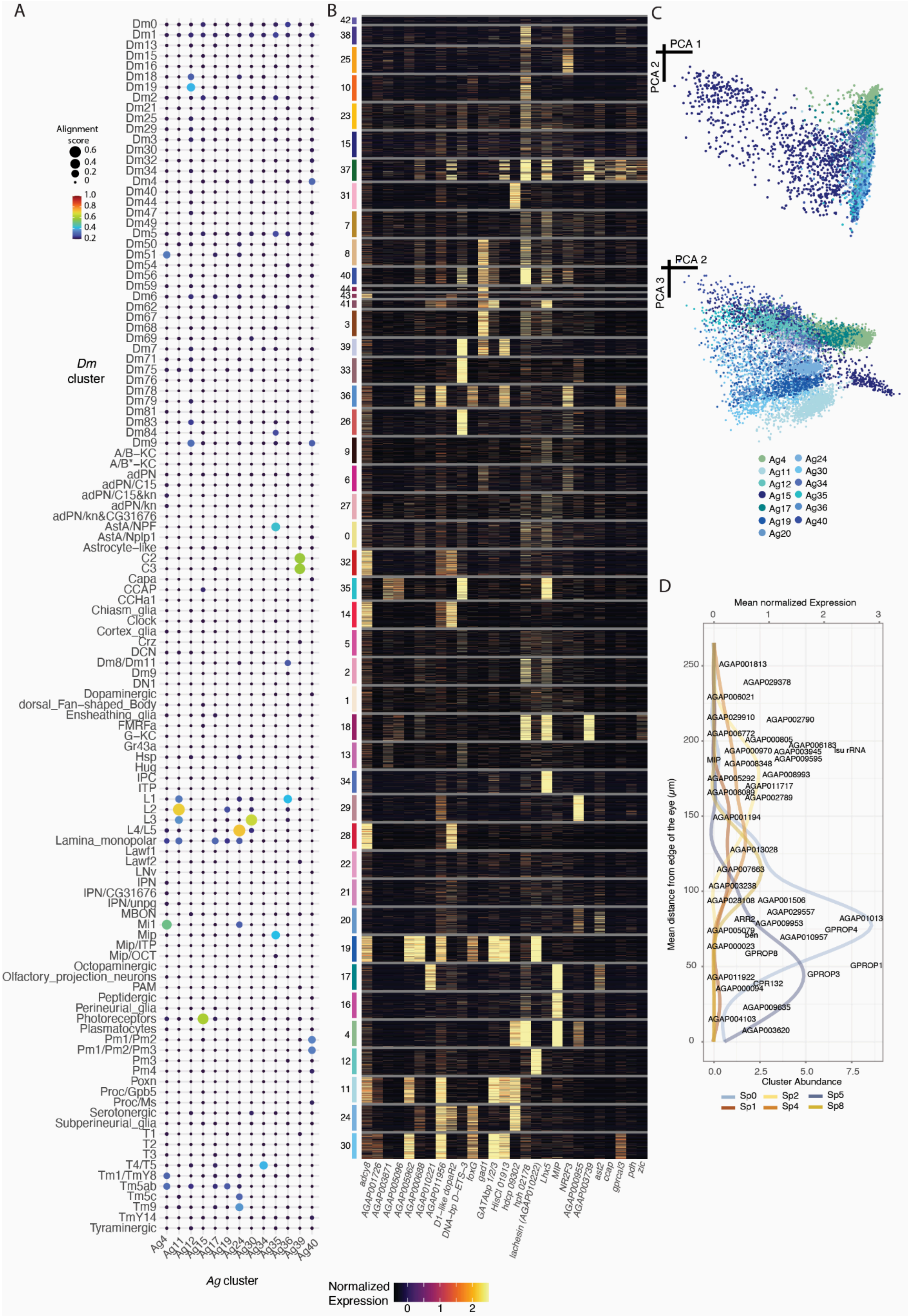

Figure S8. Characterization of optic lobes and clock neurons in the female *An. gambiae* brain. (A) *D. melanogaster* cell type assignment (SAMap) to Ag clusters compared to Davie *et al*. 2018 [19]. Dot size and color code correspond to the alignment score value for each *D. melanogaster* cell type - Ag cluster pair. **(B)**

Heatmap of expression data (normalized with SCTransform, scaled with base R scale function) downsampling a maximum of 500 cells per Ag cluster (y-axis). **(C)** PCA projections after subsetting and application of SCTransform of optic lobe clusters. **(D)** Abundance of cluster signal shown as lines (lower axis) and mean normalized expression of highly spatially variable genes (upper axis) shown as labels in one eye region of one HQ head depending on the distance in µm to the edge of the head. Line colors correspond to clusters present within the eye section. Position of gene labels correspond to their mean normalized expression as well as the position of peak expression within the region.

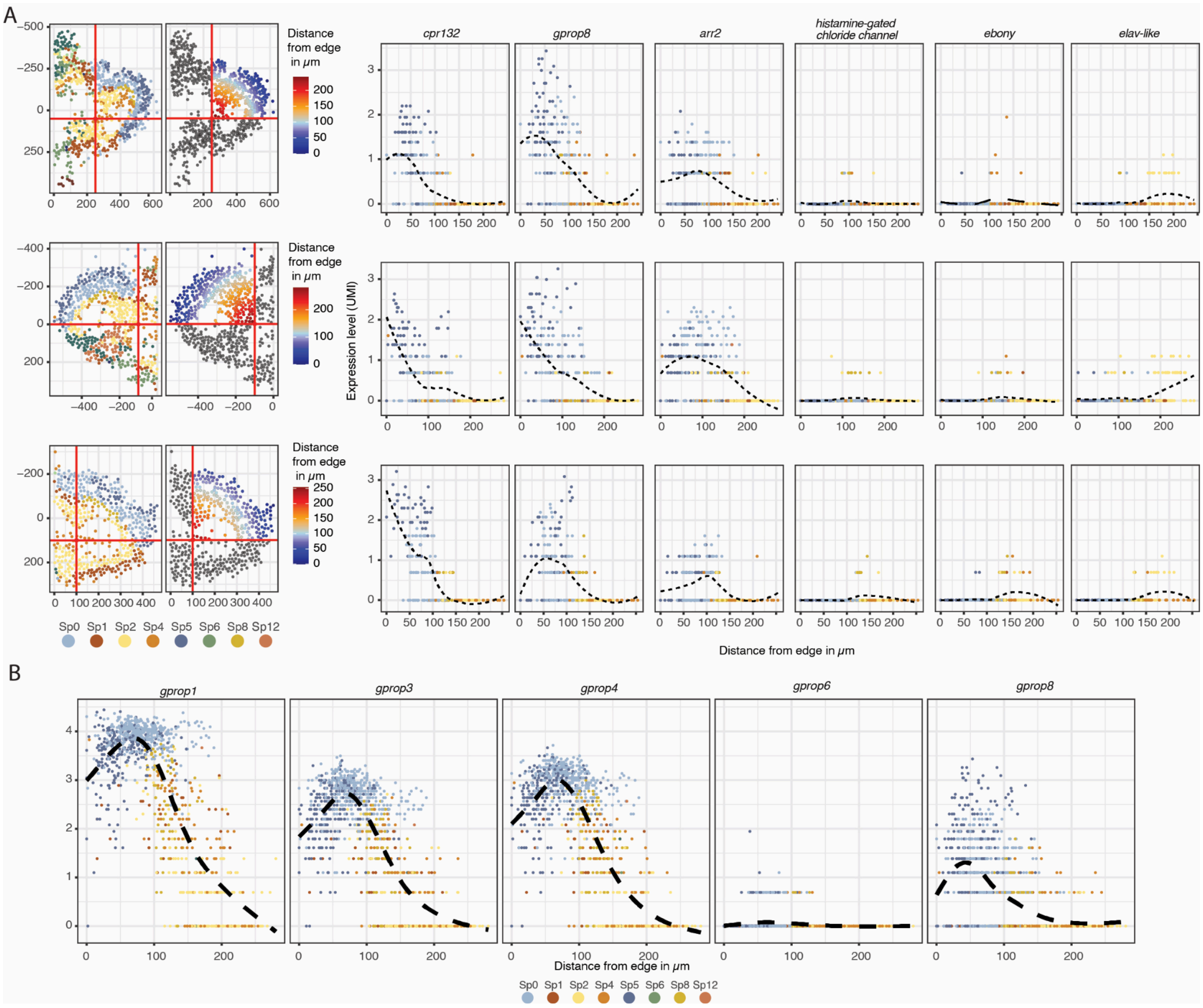

Figure S9. Distribution of vision-related genes in the head of female *An. gambiae*. (A) Section of three heads, showing the distribution of clusters within this section as well as the distance of spots to a centrally-set reference. **(B)** Distribution of opsins expression from the edge of four heads and in relation to cluster distribution.

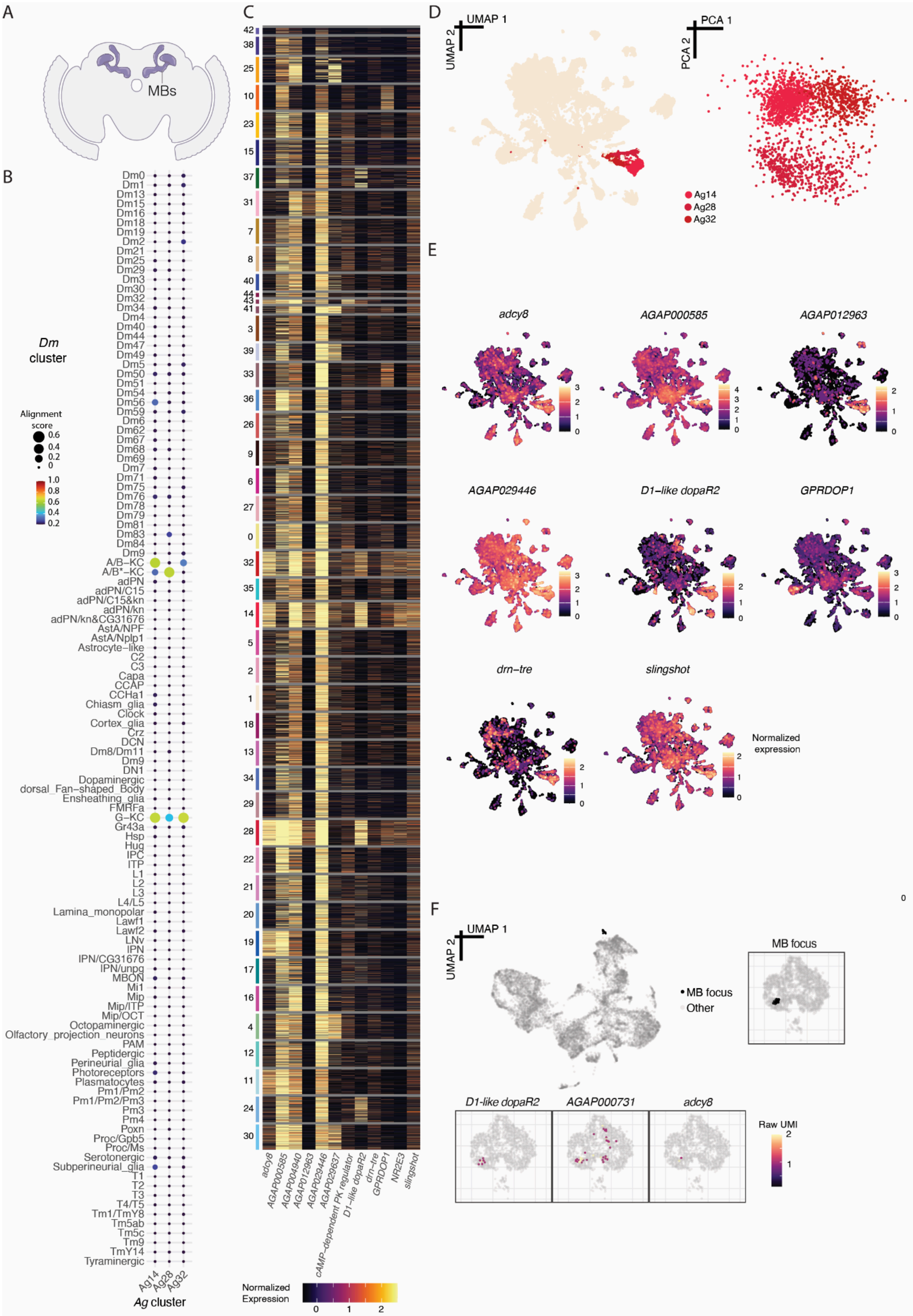

Figure S10. Identification of Kenyon cells and mushroom body in the female *An. gambiae* head. (A) Schematic of the expected putative positions of MBs in the *An. gambiae* brain. **(B)** *D. melanogaster* cell type assignment (SAMap) to Ag clusters compared to Davie *et al.* 2018 [19]. Dot size and color code correspond to the

alignment score value for each *D. melanogaster* cell type - Ag cluster pair. **(C)** Heatmap of expression data (normalized with SCTransform, scaled with base R scale function) downsampling a maximum of 500 cells per Ag cluster (y-axis). **(D)** KC clusters identification in the atlas (UMAP, left panel) and their PCA projection after subsetting and application of SCTransform (right panel). **(E)** UMAPs showing expression distribution of cell markers among cell types, expressing cells in foreground. **(F)** UMAP showing the MB focus and distribution of this MB focus in one high quality head as well as normalized expression of three markers of MBs.

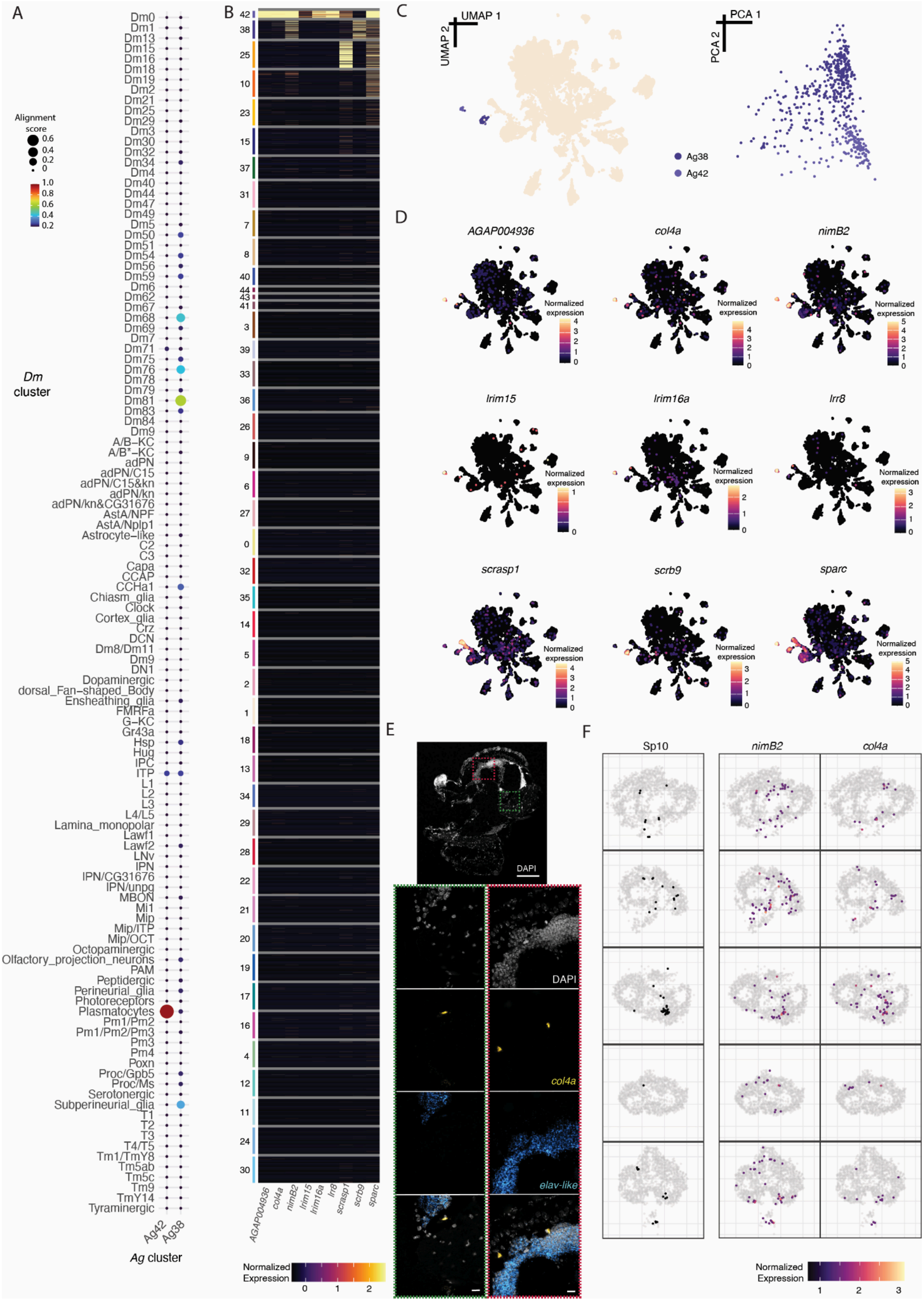

Figure S11. Characterization of immune cell populations in the female *An. gambiae* head. (A) *D. melanogaster* cell type assignment to Ag clusters compared to Davie *et al.* 2018 [19]. **(B)** Heatmap of expression data (normalized with SCTransform, scaled with base R scale function) downsampling a maximum of 500 cells per Ag cluster (y-axis). **(C)** Immune cell clusters identification in the atlas (UMAP, left panel) and their PCA

projection after subsetting and application of SCTransform (right panel). **(D)** UMAPs showing expression distribution of the oenocytoids marker *scrb9* [26] and of granulocyte marker genes (all the others) [26,75]. **(E)** ISH images on histological sections of mosquito heads. The whole section is shown on the top panel with nuclear staining (DAPI, scale bar = 100 µm, front of the head on top), and highlights the position of the insets below; transcripts of the granulocyte marker gene *col4a* are stained alongside those of the neuronal marker *elav-like* and nuclear staining (scale bar = 10 µm), highlighting granulocyte position in association with muscle (left) and cell body rind surface (right). **(F)** Distribution of Sp10 associated spots in five high quality brains (left) and normalized expression of two immune associated markers (right).

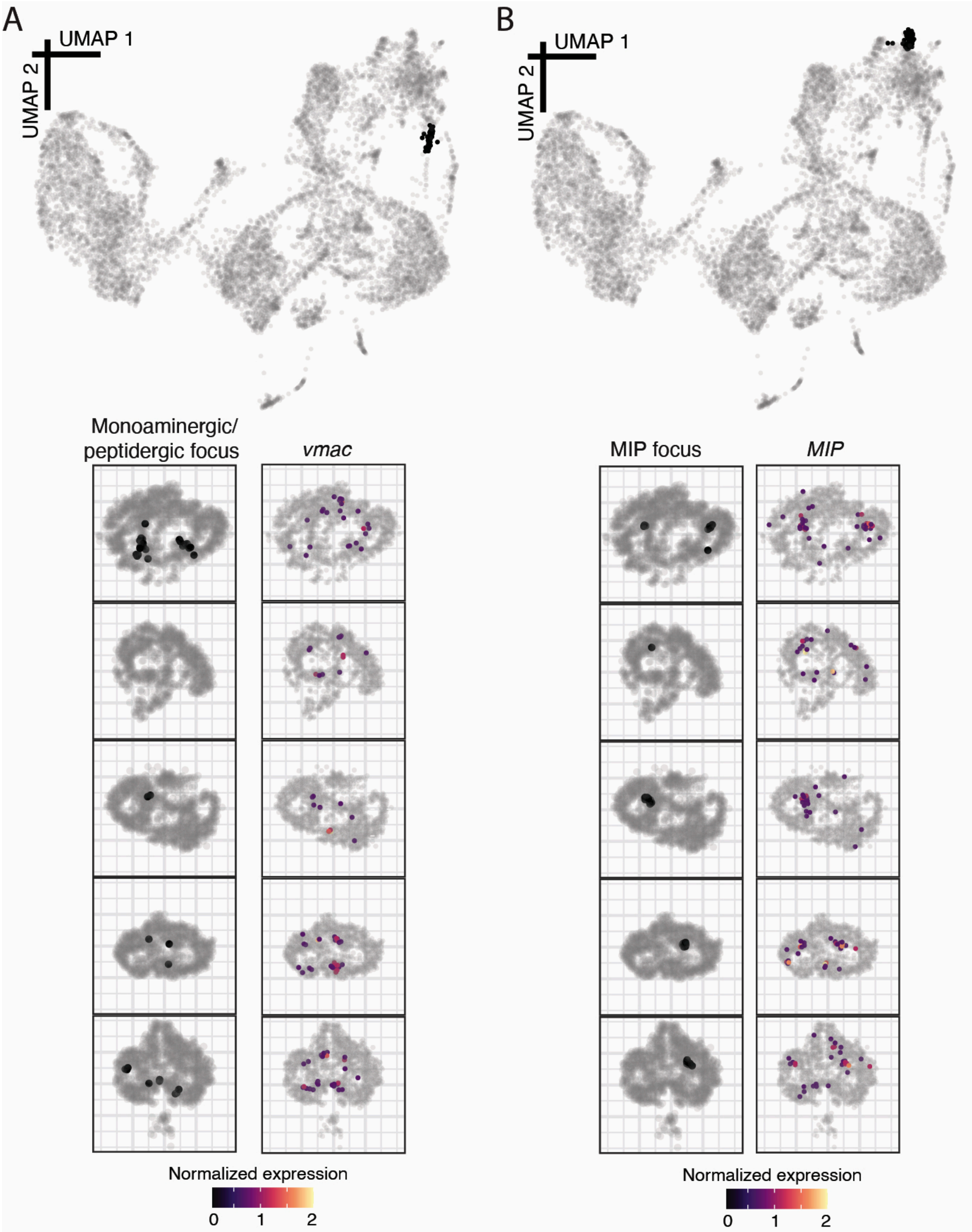

Figure S12. Spatial support for specialized neuronal foci in the cell body rind. (A) UMAP of the spatial transcriptomic dataset highlighting the *vmac* expressing focus identifying monoaminergic/peptidergic related spots shown in their spatial context**. (B)** *MIP*-expressing focus identifying MIP-related spots shown in their spatial context.

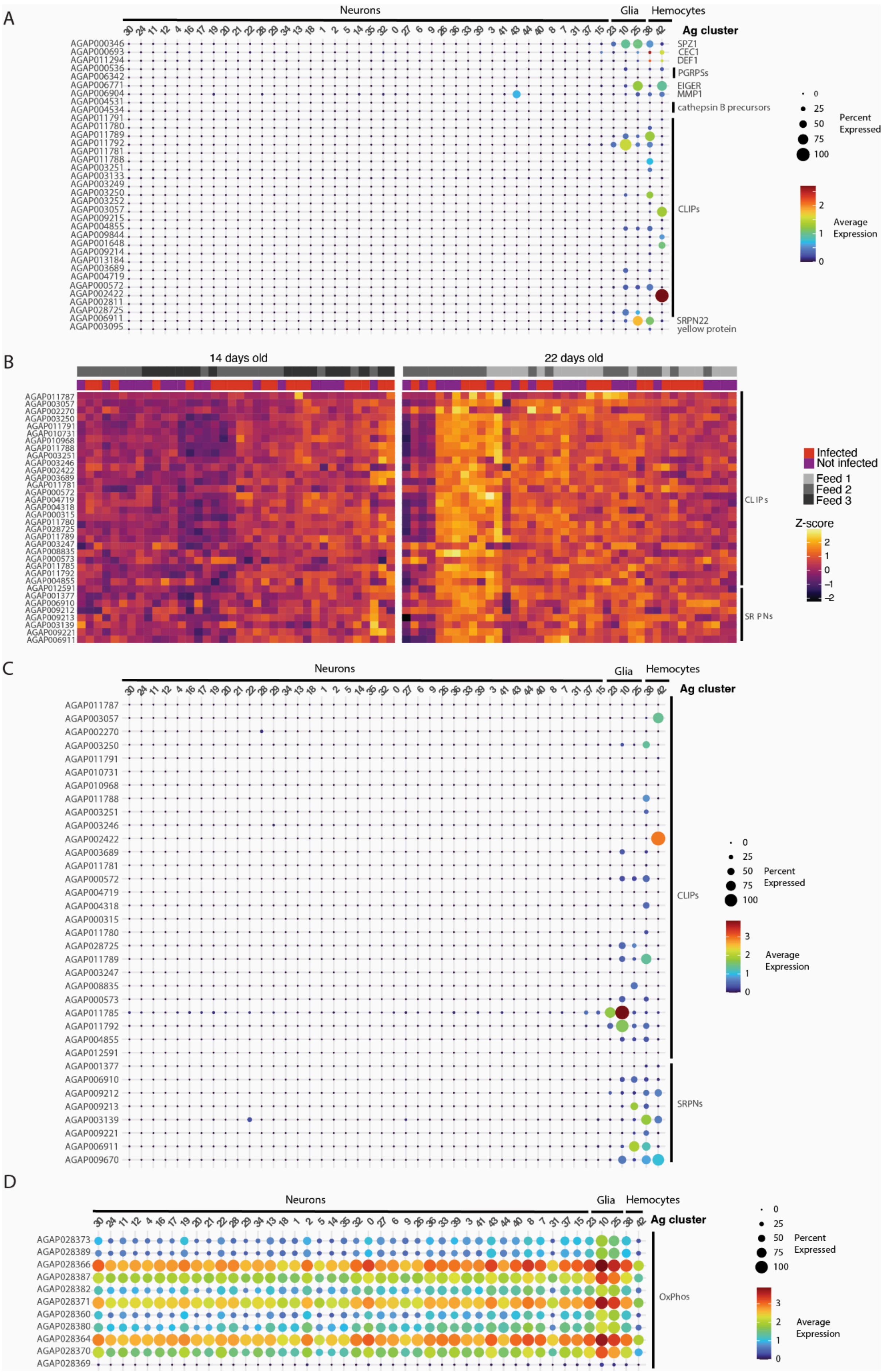
A
Neurons
Glia
Hemocytes
Ag cluster
30 24 11 12 4 16 17 19 20 21 22 28 29 34 13 18 1 2 5 14 35 32 0 27 6 9 26 36 33 39 3 41 43 44 40 8 7 31 37 15 23 10 25 38 42
AGAP000346
AGAP000693
AGAP011294
AGAP000536
AGAP006342
AGAP006771
AGAP006904
AGAP004531
AGAP004534
AGAP011791
AGAP011780
AGAP011789
AGAP011792
AGAP011781
AGAP011788
AGAP003251
AGAP003133
AGAP003249
AGAP003250
AGAP003252
AGAP003057
AGAP009215
AGAP004855
AGAP009844
AGAP001648
AGAP009214
AGAP013184
AGAP003689
AGAP004719
AGAP000572
AGAP002422
AGAP002811
AGAP028725
AGAP006911
AGAP003095
SPZ1
CEC1
DEF1
PGRPSs
EIGER
MMP1
cathepsin B precursors
CLIPs
SRPN22
yellow protein
0
25
50
75
100
Percent Expressed
2
1
0
Average Expression
B
14 days old
22 days old
AGAP011787
AGAP003057
AGAP002270
AGAP003250
AGAP011791
AGAP010731
AGAP010968
AGAP011788
AGAP003251
AGAP003246
AGAP002422
AGAP003689
AGAP011781
AGAP000572
AGAP004719
AGAP004318
AGAP000315
AGAP011780
AGAP028725
AGAP011789
AGAP003247
AGAP008835
AGAP000573
AGAP011785
AGAP011792
AGAP004855
AGAP012591
AGAP001377
AGAP006910
AGAP009212
AGAP009213
AGAP003139
AGAP009221
AGAP006911
CLIPs
SRPNs
Infected
Not infected
Feed 1
Feed 2
Feed 3
Z-score
2
1
0
-1
-2
C
Neurons
Glia
Hemocytes
Ag cluster
AGAP011787
AGAP003057
AGAP002270
AGAP003250
AGAP011791
AGAP010731
AGAP010968
AGAP011788
AGAP003251
AGAP003246
AGAP002422
AGAP003689
AGAP011781
AGAP000572
AGAP004719
AGAP004318
AGAP000315
AGAP011780
AGAP028725
AGAP011789
AGAP003247
AGAP008835
AGAP000573
AGAP011785
AGAP011792
AGAP004855
AGAP012591
AGAP001377
AGAP006910
AGAP009212
AGAP009213
AGAP003139
AGAP009221
AGAP006911
AGAP009670
CLIPs
SRPNs
0
25
50
75
100
Percent Expressed
3
2
1
0
Average Expression
D
Neurons
Glia
Hemocytes
Ag cluster
AGAP028373
AGAP028389
AGAP028366
AGAP028387
AGAP028382
AGAP028371
AGAP028360
AGAP028380
AGAP028364
AGAP028370
AGAP028369
OxPhos
0
25
50
75
100
Percent Expressed
3
2
1
0
Average Expression

Figure S13. Differentially expressed genes of the female *An. gambiae* brain in different life stages and aging, and association of DEGs to putative cell types of origin. ***(A)*** Mapping of immune genes upregulated after bloodmeal to the cell atlas clusters. Notable gene expression (z-score) shown in the 45 Ag clusters of the atlas (Ag cluster ID and main cell type classification on the top line); gene annotation shown on the right. **(B)** Heatmap showing the genes belonging to immune gene families (on the right line) enriched in 22 day-old compared to 14 day-old mosquitoes (right and left panels, respectively), samples hierarchically clustered with a complete-distance method. **(C)** Mapping of immune genes upregulated with aging (in 22 day-old compared to 14 day-old mosquitoes) to the cell atlas clusters. Notable gene expression (z-score) shown in the 45 Ag clusters of the atlas (Ag cluster ID and main cell type classification on the top line); gene annotation shown on the right. **(D)** Mapping of the most upregulated genes with aging (in 22 day-old compared to 14 day-old mosquitoes) to the cell atlas clusters. Notable gene expression (z-score) shown in the 45 Ag clusters of the atlas (Ag cluster ID and main cell type classification on the top line); gene annotation shown on the right.
