# Supplementary material for: The brain of the female Anopheles gambiae mosquito at spatial and single-cell resolution
Source: Nat Commun. 2026 Sep 17;17:10940. doi: 10.1038/s41467-026-76839-z (PMC13680466; doi:10.1038/s41467-026-76839-z)
Supplement: Supplementary file 3 [file 41467_2026_76839_MOESM2_ESM.pdf]

nature portfolio

Peer Review File

The brain of the female Anopheles gambiae mosquito at spatial and single-cell resolution

Corresponding Author: Dr Arthur Talman

This file contains all reviewer reports in order by version, followed by all author rebuttals in order by version.

Version 0:

Reviewer comments:

Reviewer #1

(Remarks to the Author)
This manuscript presents a single-cell and spatial transcriptomics atlas of the Anopheles gambiae brain, aiming to provide insight into mosquito neuroanatomy and gene expression. While the topic is interesting and relevant for both vector biology and comparative studies across insects, the quality of the work presented here falls well below the methodological and analytical standards expected for publication in Nature Communications. The findings are neither robust nor sufficiently novel to be of broad interest, and many of the conclusions are not convincingly supported by the evidence.

Major comments:
• The use of the Davie et al. 2018 Drosophila dataset for cross-species cell-type mapping is unfortunate. This dataset is shallow compared to more recent atlases, and fails to reliably identify certain key neuronal populations. Data from Park et al., 2022 for the central brain and Konstantinides et al., 2018 or Ozel et al., 2020 for the optic lobes would have been more suitable.
• The rationale for including spatial transcriptomics is unclear, and the resulting data does not contribute meaningfully to this study. The analysis mostly confirms expected anatomical patterns, for instance, photoreceptor clusters appearing in the optic lobe region, with little new insight. The resolution is too low to accurately resolve relevant structures, making several conclusions from this experiment unreliable.
• The cluster annotation contains several major issues that question the reliability of the entire analysis, with several orthologue assignments between fly and mosquito being plainly incorrect. For example, AGAP000585 is a more likely orthologue of CG6118 than mamo. And even if it was a real mamo orthologue, mamo is expressed in both γ and α′β′ Kenyon cells in Drosophila, so this gene cannot be considered a specific α′β′ marker. Similarly, AGAP006107, not AGAP029446, is the trio orthologue in Anopheles. The gene Bx (AGAP000731) is absent from α′β′ Kenyon cells in Drosophila, contradicting the proposed annotation of cluster 28. These errors indicate a lack of biological validation throughout the whole manuscript. While the proposed Kenyon cell clusters 14, 28, and 32 are plausible, the supporting evidence for their subdivision into αβ, α′β′, and γ types is not credible.
• The absence of several memory-relevant genes in KCs, including Dop1R1 as well as elements of memory-relevant signal transduction such as rut and PKA subunits, is a concern. The authors should discuss what consequences the absence of these crucial genes might have on how mosquitoes learn.
• The OA neuron cluster (cluster 7) is also not convincingly identified. Although this cluster expresses Vmat, it lacks octopaminergic markers such as Tbh and Tdc2; and Dh31 is not an OA neuron marker in flies. The reported number of OA neurons is also inconsistent with expected numbers from Drosophila. Cluster 7 likely contains a mixture of cell types, including multiple peptidergic types, and therefore must not be reliably called octopaminergic.
• The possibility that clusters 37 and 18 could represent LNvs is inconsistent with expected cell numbers: flies have only 16 LNvs, and a sudden expansion in mosquitoes seems unlikely. Instead, these clusters likely represent a mixture of cell types, including possibly a minority of LNv cells. Related to this, statements such as “LNv cellular bodies have a spatially restricted distribution in the Anopheles brain” are trivial – aren’t all cell bodies spatially restricted?
• The analysis of mating and blood feeding effects is interesting but unfortunately represents a too small portion of the study, and lacks sufficient validation or further exploration of the results. Crucially, one could argue that mosquitoes maintained on sugar-only diets for 6 days after mating are effectively protein-deprived, raising questions about whether the observed transcriptional effects reflect genuine blood meal-induced physiological transitions, or simply the fulfilment of protein requirements. Better controls would include a blood meal immediately after mating, or providing a protein-supplemented sugar solution instead of blood.

Minor comments:
• Table S2 lacks essential statistical information (LogFC, p-values) for marker genes, making it impossible to evaluate marker strength without having to constantly refer to table S3.
• The authors attribute unidentifiable clusters to "technological limitations of cell state capture, […]." They should better explain what they mean here. If they believe that their approach cannot capture cell states accurately, this should be addressed experimentally, rather than speculated.
• The manuscript provides too little analysis of fast-acting neurotransmitters in individual clusters. A more detailed characterization would be helpful.
• The statement "An2 was associated to 9 D. melanogaster olfactory projection neuron subtypes (Fig. S3) suggesting it is composed of cells of the antennal lobe" is inaccurate, since olfactory PNs are not uniquely localised in the antennal lobe: only their dendrites are.
• "This could be due either to (i) a transcriptionally-silent parasite manipulation phenotype, (ii) subtle changes below the detection threshold (e.g., changes only in a small group of cells), (iii) an ineffective parasitic manipulation in our specific parasite-mosquito strains combination and lab conditions." These possibilities must be further explored to provide a valid explanation for the unexpected absence of clear gene regulation in the brains of Plasmodium-infected animals.
• A mention of the circadian phase would be useful for all experiments, especially as transcription in clock neurons is being assessed.

Reviewer #2

(Remarks to the Author)
This manuscript provides a comprehensive single-cell RNA-sequencing atlas of the female Anopheles gambiae brain, complemented by spatially resolved transcriptomics using Slide-seq. The authors identify 45 cell clusters and map their spatial organization within the mosquito head. They further explore transcriptional signatures associated with key life history events, including mating and blood-feeding, using bulk RNA-seq on individual brains. The work establishes a foundational resource for understanding the neural basis of mosquitoes, with implications for non-model insect neurobiology. The study is innovative, technically robust, and addresses a critical gap in the neuroanatomy of disease vectors. The integration of scRNA-seq with spatial data is strong, offering novel insights into glial diversity and visual system adaptations in Anopheles. However, there are opportunities to enhance the analysis of physiological state-dependent changes.
1. This work emphasizes transcriptional changes linked to mosquito life history events (mating, blood-feeding, Plasmodium falciparum infection, and age). The single-cell atlas is derived from different physiological states of individuals, but only bulk RNA-seq results are compared. The authors should clarify in the abstract and results that these life history comparisons are bulk-based, while explicitly addressing how they inform cell-type-specific interpretations (e.g., via mapping DEGs to the atlas clusters). More critically, the significance of these comparisons lies in their potential to reveal state-dependent shifts in cell-type composition or gene expression profiles—e.g., whether mating or blood-feeding alters neuronal subtype proportions, glial activation, or cluster-specific transcriptional states. Currently, it remains unclear how such differences manifest at the single-cell level, limiting insights into dynamic neural remodeling during vectorial behaviors. The study could be strengthened by analyzing scRNA-seq data from virgin, mated, and blood-fed conditions, which would directly link bulk observations to cellular heterogeneity.
2. Accurate identification of cell types is paramount in this first-of-its-kind single-cell atlas of the Anopheles brain. The authors' use of SAMap for mapping to a Drosophila brain dataset is a solid foundation. However, the analysis relies solely on a single Drosophila brain dataset, which may limit resolution at the subcluster level, particularly for specialized regions. Incorporating additional Drosophila datasets (e.g., PMID: 33125872 and PMID: 33149298) would potentially resolve ambiguities in unannotated clusters and strengthen claims of Anopheles-specific adaptations. Furthermore, leveraging single-cell brain atlases from other insect species could broaden the comparative framework. For instance, integrating data from ants (PMID: 35711063) and honeybees (PMID: 40199930) would facilitate multi-species homology assessments and highlight mosquito-specific divergences. This would elevate the atlas's utility as a cross-insect resource without substantial new data generation.
3. All downstream analyses, including spatial mapping, neurotransmitter profiling, and life history event integrations, hinge on the accuracy of cell-type annotations. I have several questions and concerns regarding specificity, particularly for olfactory and visual neuron subtypes. The evidence for the olfactory projection neuron identity of cluster An2 (labeled as adPN-IP-OPN in Fig. 1C) is not fully convincing. While vacht is appropriately highlighted as a cholinergic marker, it is not a canonical OPN-specific gene. In Drosophila, VAChT broadly denotes excitatory projection neurons but lacks subtype specificity for antennal-driven or inter/intra-projection classes. Moreover, vacht exhibits widespread expression across multiple clusters (e.g., An0, An4, An11, An12, An16, An17, An19, An20, An21, An22, An24, An28, An29, An30, An32, An34, An35; Table S1), diluting its utility as a defining feature for An2. Compounding this, Fig. S3 indicates high SAMap similarity of An2 to diverse Drosophila types, including Proc/Grob5 progenitors, suggesting potential overlap with precursor states rather than mature OPNs. To bolster this annotation, the authors should evaluate canonical OPN markers from Drosophila studies, such as oaz or GH146/acj6, assessing their expression in An2. If absent or low, consider reclassifying An2 as a broader cholinergic PN or uncommitted cluster.
Similarly, marker overlap complicates other assignments. For instance, drgx (AGAP008868) is proposed as a marker for Tm1/TmY8 (An16) and T4/T5 (An20) neurons (Fig. S8A, B), yet expression data show it in both clusters (Fig. S8B). However, SAMap aligns Tm1/TmY8 more strongly to An20 (Fig. S8A), casting doubt on An16's designation and risking misattribution of visual processing roles. Broader scrutiny of Table S1 reveals several such multi-cluster markers, which may reflect technical noise or annotation artifacts.

Minor Points:
1. It would be helpful to include information on the number of replicates (N = #) in the figure legends.

2. Displaying Drosophila homolog names alongside Anopheles gene IDs would greatly improve interpretability.
3. The mosquitoes used for spatial transcriptomics are described as seven-day-old An. gambiae females, but their physiological state (e.g., virgin, mated, or blood-fed) is not specified.
4. In Fig. 4D, the authors describe gene expression patterns corresponding to the optic lobe (OL) layers. To support this interpretation, the authors could provide additional examples across multiple brain sections to demonstrate that the observed spatial relationships are reproducible across samples.
5. Marker Gene Expression Analysis. Given the inherently sparse nature of single-cell data, randomly selecting 500 cells for visualization (Fig. S7B, S8B, S9C, S10B) may not fully capture the overall expression patterns. The authors could consider averaging expression levels across defined cell groups or performing repeated random sampling to assess the consistency of the observed trends.
6. The Methods section needs more precise descriptions. The current protocols lack many details.

Reviewer #3

(Remarks to the Author)
This study presents the first cellular and spatial atlas of the Anopheles gambiae female brain, establishing a foundational resource for understanding neurobiology in a critical malaria vector. Researchers generated a comprehensive single-cell and spatially-resolved transcriptomic map of the Anopheles gambiae brain by combining single-cell RNA sequencing with Slide-seq, allowing them to precisely map cellular diversity and gene expression patterns onto the brain's physical structure. This integration of techniques represents a significant technical achievement in non-model insect neuroscience, and, to my knowledge, it is the first spatial transcriptomics work in mosquitoes. Beyond mapping brain anatomy, the study aims to identify how the brain's transcriptome responds to mating, blood-feeding, Plasmodium infection, and aging using bulk RNA seq.

In my opinion, this is an important and interesting dataset that would be a critical resource for researchers investigating the malaria mosquito biology and physiology, and can help future researchers in combining multiple transcriptomics data sets.

Below, I include suggestions for the authors that I think would be useful to implement.

1. Currently the introduction lacks scholarship and the importance of this work. To make this paper more suited for a general reader, it should be expanded. Some information that can be added: What was previously known in the literature, and what was previously done. How can a combination of single-cell sequencing, spatial transcriptomics, and bulk RNA sequencing inform new insights and expand on what they did and find? What is the importance of this study?

2. Annotation of all neurons is a major part of the single-cell brain atlas. The authors write that "...expression of cellular markers for neurons (elav-like) and glia (repo) as these markers are widely conserved across the animal kingdom 15,16, including in dipterans 13." Is there direct evidence of elav-specific and pan-neuronal expression in the adults An. gambiae? If so, please cite. If not, did the data reveal other pan-nuronal markers that can be used to classify neurons (brp/Syt1/nSyb see: PMID: 34590074, PMID: 35239393, PMID: 40060408), and why weren't they used?

3. The annotation of the single-cell brain atlas combines six 10x runs from three biological conditions (virgin, mated, blood-fed). While increasing cell numbers enables enhanced annotation power, combining different conditions may introduce batch effects or technical and biological variability. Did the authors use an approach to check and account for these effects across the six pools? Please clarify in the main text or methods. Also, explicitly state in the main text and figure legend that the atlas merges data from multiple conditions.

4. The section titled "Life history is associated with distinct brain transcriptional states" in the paper is the weakest and should be reanalyzed. First, there is currently no analysis presented for the single-cell brain conditions (virgin, mated, blood-fed), and it isn't clear why those experimental conditions were performed. Instead, this section is focused on single brain bulk RNA seq. Was there any cluster-based differential analysis (MAST or any other) performed between these conditions in the single-cell data? If such analyses were performed, did they identify differentially expressed genes at the cluster or cell-type level? Clarifying whether these fundamental analyses were done is essential, as they are at the base of the single-cell experiments. Do the differential analysis results from the single-cell analyses align with the findings from the bulk RNA data in Figure 5? If not, what are the observed differences, and what might explain these discrepancies? Clarify whether and how these datasets agree or diverge.

5. The single brain bulk RNA-seq aims to link virgin, mated, and blood-fed conditions to gene expression changes. Using a single brain reduces variation and was well executed. However, how can cell types be reliably linked to bulk sequencing results (Figure 5B, E, and J)? Did any computational deconvolution analysis to estimate cell type proportions or changes in cell type-specific expression across conditions using the single-cell atlas was performed? Please clarify this in the main text and methods. Additionally, discuss the substantial limitations of such analysis in the main text and discussion.

6. Given the lack of transcriptional changes after P. falciparum infection, how do the authors interpret these results in the context of existing literature? What possible explanations, such as sample timing, sample size, or other factors, might account for these findings? Further discussion of the implications and how these results relate to previous work should be added.

7. Finally, this work shows the complexity of the mosquito brain, using both single-cell RNA sequencing and a spatial

transcriptomics approach. Given these advancements, the use of bulk RNA sequencing alone may be insufficient to capture the full complexity of transcriptional responses to mating, blood-feeding, or infection in the mosquito brain, potentially missing critical cell-type-specific or spatially localized changes and should be clarified and further discussed in the paper.

8. The discussion part of this paper is practically missing, while the authors added a conclusion of their work; discussing their findings is crucial to understanding their importance. This part may include a comparison to recent literature, hypotheses about the gene families and specific genes found in Figure 5, and the importance or implications that the work might have. In addition, as this work is focused on computational analysis, a limitation of those experiments and analyses should be discussed. Other discussion points are suggested in some of the points raised.

9. The paper will benefit from deeper discussion of mosquito-specific neurobiological features and comparisons within Diptera. The authors should discuss their findings in context of other mosquito brain studies, such as PMID: 36277452, PMID: 40060408, PMID: 37182106.

Minor comments:

10. Please change the title of this atlas to "The Anopheles gambiae female mosquito brain at spatial and single-cell resolution". As anopheles research advances, it would be beneficial to know which species and sex the paper refers to.

11. Similarly, there is an inconsistency in using Anopheles throughout the paper and figure titles. In some places, the authors are using only Anopheles, and in other places, An. gambiae (and other times An gambiae). As this work relates only to Anopheles gambiae, the authors should state the species name everywhere and spell it “An. gambiae”.

12. The use of CNS/central nervous system in the results of this work is misleading, as the work profiles only the brain and doesn’t touch any parts of the ventral nerve cord or other ganglia.

13. The first paragraph in the results (and Figure S1A) mentions 6 pools - it is not clear which pools are being referred to. Is a pool a single 10x run? Please clarify in the text. In addition, Figure S1A/C states that 2/3 batches were used. Please clarify what batches are.

14. Figure S1 visualization is informative and clear. However, it lacks an appropriate figure legend to help the reader follow the experimental workflow without turning to the method section.

15. In Table S1, the brackets after the references do not correlate to the correct citation number and should be fixed.

16. In Figure 1A, the pie charts can use more information on them - how many clusters they represent and how many total cells each section represents.

17. In Figure 1A legend, there is a tilde (~) next to the 48,806 cells - is this an estimated number?

18. QC matrices for the single-cell atlas should be more readable and should include more information (how many cells were collected per sample?). In Figure S2, the violin plots aren’t readable - using a log scale will enable showing the distribution better. In addition, the average numbers (both total and per sample) should be noted on the figure/legend or in a separate supplementary table to enable comparison.

19. The authors used SAMap to compare their data to a fly brain data set to help with annotating their cluster. This should be explained in detail in the method section, including the strategy and parameters used, and which of the data from PMID: 29909982 was used. In addition, they need to explain if and how differences in the data set were evaluated (for instance, sample size, male fly brains).

20. In the “Spatial organisation of the female An. gambiae head“ results, the authors write “However, we noted minor differences compared to the Drosophila brain, such as the different substructures of the central complex, the number of antennal glomeruli, or the different lobes of the mushroom body (Fig. S4)”. The authors should expand on the differences they noted.

21. The functional roles of identified glial subtypes in Anopheles gambiae mosquitoes lack direct experimental validation. This is particularly important given the unexpected co-expression patterns of markers that are mutually exclusive in Drosophila, suggesting potential divergence in glial cell functions. While molecular signatures suggest conservation of glial functions, direct experimental validation will be needed to assess if those observations are correct. I don’t think that there is need for additional experimental validation, in this paper, however the authors should acknowledge and discuss this in the text.

22. Similarly, the authors should discuss how the spatial organization of the vision-associated and clock genes aligns with insect brain regions known to be involved in vision and circadian rhythms. Acknowledge the need for functional studies as a key future direction.

23. In Figure 2A, the white arrow isn’t visible and should be changed to a more visible color.

24. In Figure 4E and F, the data describe the localization of identified clock genes. Was this seen in the single-cell data?

How does the expression of those genes look? and can you use it to identify clock cells outside cluster An37?

25. Data accessibility:
As both the raw sequencing data and Github page were not available at the time of this review, I couldn't assess them.
To increase data accessibility and to allow mosquito researchers easy visualization, the annotated brain single-cell data (including condition information) should be upload to one of the freely available visualization tools like: SCope, ASAP or UCSC Cell Browser.

26. In addition, adding a supplementary table of gene expression from all bulk RNA-seq data (as TPM or other normalized counts) will allow researchers easy access to the data (similar to what was uploaded to the drive.ird.fr).

27. In the method section, mosquito sample conditions - "FigS1A" should be Fig. S1A.

28. In Figure S3 legend, there is a space missing after the word each, "eachD. melanogaster" should be each D. melanogaster.

29. In the "A spatially-resolved map of cellular diversity in the An. gambiae brain", Glia section, the gene threonine 3-dehydrogenase (AGAP011948) is missing the gene name thr3DH that appears on the figure- should be threonine 3-dehydrogenase (thr3DH; AGAP011948).

30. Currently, the text mixes American and British English spellings for example, we both "behavior" and "behaviour," "organization" and "organisation," and "color" and "colour"are spelled in different sections. This should be consistent throughout.

Version 1:

Reviewer comments:

Reviewer #1

(Remarks to the Author)
The manuscript has improved substantially. The updated cell type annotation is now much more accurate and the added analyses on various cell types and fast-acting neurotransmitters make the study more complete. The section about life history is also improved, even if it feels like this section could stand as one or several separate studies. However, the following concerns remain:

1. A few issues in terms of cluster annotation and choice of marker genes. First, given the diversity of genes expressed in Ag7, I recommend that the authors annotate it as "monoaminergic and peptidergic" rather than purely monoaminergic. Further, even though it appears as a marker of Ag7, Dh31 is not an appropriate marker for monoaminergic neurons, and Vmat should be used in Fig S12 to identify these cells instead. Regarding Ag37, expression of key markers is not sufficiently uniform to justify naming the whole cluster "clock neurons". I would recommend a more careful annotation (e.g., "clock neuron enriched").

2. I am not convinced by the authors' response to my comments regarding the spatial transcriptomics data, which focuses mostly on the novelty (which I recognise) but brushes off my concerns about the limited resolution and usefulness of this dataset. Importantly, Fig S5E suggests only minimal correlation between single-cell and spatial clusters, an issue that is not sufficiently addressed or discussed. Several figures obtained from this data (3C, 4E, etc) provide little interpretable information beyond very basic mapping of gene expression, mostly restricted to the optic lobes. As a result, many of the conclusions drawn from the spatial transcriptomics data appear confusing or limited in strength. For example, mapping of Sp12 shows a lateral aggregate of putative clock neurons similar to that observed in related species, but the absence of a dorsal population is not addressed, leaving the reader guessing whether this population is missing in An. gambiae or simply not visible in the data. In this situation, I recommend a clear shift in emphasis: the spatial transcriptomics should be presented explicitly as a preliminary, low-resolution mapping resource. In contrast, greater weight should be placed on the in situ hybridization experiments, which appear more accurate and informative for validation and mapping. Accordingly, the authors should revise the manuscript to carefully temper and clarify ambiguous conclusions arising from the spatial transcriptomics data. Analysis of cellular anatomy would also benefit from clearer signposting of similarities and differences with related species to clarify which features are conserved, or specific to An. gambiae.

3. The response to my concern about mosquitoes being potentially protein deprived is flawed. The fact that protein deprivation in sugar-fed female Anopheles was never reported does not mean that it is not a possibility. Unaffected lifespan also does not rule out a protein deprivation state: lifespan is barely affected in protein-deprived Drosophila, despite them developing strong protein cravings and clear physiological shifts. Therefore, the authors should acknowledge that transcriptional changes attributed to the blood meal may be influenced by prolonged protein deprivation in sugar-fed controls.

Additional (minor) comment: Fig S5: cluster names are still 'An', not 'Ag'.

Reviewer #2

(Remarks to the Author)
The authors have provided a comprehensive revision that addresses my concerns.

Reviewer #3

(Remarks to the Author)
I thank the authors for their thorough and constructive revision. They have addressed all of my original comments satisfactorily. The introduction now provides the scholarly framing and motivation that were previously missing. The addition of pan-neuronal classification, the documentation of batch-effect handling and merged design, and the new cluster-level single-cell differential expression analysis integrated with the bulk RNA-seq results substantially strengthen the "Life history" section and address my central concern regarding the linkage of transcriptional changes to cell types. The Discussion has been developed into a well-contextualized section, and the data and code have been made publicly available. This revised manuscript represents a valuable resource and a clear improvement over the original submission. I am happy to support publication once a few minor remaining points are addressed.

Minor comments

1. Two editing artifacts remain in the figure legends and should be removed. The Figure S4 legend ends with the note "Scale bar 50 µm update legend." The Figure S9 legend contains a duplicated fragment from the Figure 4 caption: "Distribution of vision-related genes… Figure 4. Optic lobe and circadian neurons in the visual system of An. gambiae…"

2. There is a gene-ID inconsistency for the brp ortholog. Figure 1B refers to AGAP010286, while the Figure 1B legend lists AGAP010296. Please reconcile these identifiers and confirm the correct one is used consistently throughout the manuscript.

3. The replicate numbers for the bulk life-history data appear inconsistent. The Figure 5 legend states n = 39 single-brain samples, whereas the Methods section lists virgin (n = 9), mated (n = 10), and blood-fed (n = 10), totaling 29. Please verify the sample count and reconcile the figure legend with the Methods section.

4. In Table S7, the Ag37 genes described as down-regulated after mating (GPROP3, arrestin-1, GPROP4) have positive log2FC values, indicating upregulation under the table's mated-versus-virgin convention. Additionally, AGAP029557 does not appear in the Ag37 significant list. Please reconcile the direction of regulation described in the text with the supplementary table and confirm the accuracy of the gene list.

Open Access This Peer Review File is licensed under a Creative Commons Attribution 4.0 International License, which permits use, sharing, adaptation, distribution and reproduction in any medium or format, as long as you give appropriate credit to the original author(s) and the source, provide a link to the Creative Commons license, and indicate if changes were made.
In cases where reviewers are anonymous, credit should be given to 'Anonymous Referee' and the source.
The images or other third party material in this Peer Review File are included in the article’s Creative Commons license, unless indicated otherwise in a credit line to the material. If material is not included in the article’s Creative Commons

license and your intended use is not permitted by statutory regulation or exceeds the permitted use, you will need to obtain permission directly from the copyright holder.
To view a copy of this license, visit https://creativecommons.org/licenses/by/4.0/

Point-by-Point Response to Reviewers

We are deeply grateful to all three reviewers for their thorough and constructive assessment of our manuscript. Their critiques have been invaluable in guiding a comprehensive revision that substantially strengthens the robustness and breadth of our work. In response to the reviewers' concerns, we have undertaken an extensive series of new analyses that expand and deepen the original study. These include: (i) a systematic multi-species cross-comparison of our single-cell atlas against six insect brain datasets — spanning *Drosophila* central brain, *Drosophila* visual system, *Aedes aegypti*, ant, and honeybee — yielding a markedly more robust and biologically validated consensus annotation; (ii) a full single-cell RNA-seq differential expression analysis (DEA) across the three experimental conditions (virgin, mated, blood-fed), revealing cell-type-specific transcriptional reprogramming invisible to bulk approaches; (iii) a revised and substantially more conservative cluster annotation pipeline incorporating multi-atlas SAMap alignment, orthology validation, and neurotransmitter profiling; and (iv) an expanded and rewritten *Discussion* section contextualizing our findings within the broader landscape of insect neurobiology. Below we address each point in detail.

Please note that *Anopheles* clusters have been renamed as 'Ag clusters' (instead of 'An clusters') throughout the manuscript to improve readability.

REVIEWER #1

We thank Reviewer #1 for their rigorous and detailed critique. Addressing each point necessitated a thorough re-examination of our analytical framework and led to substantial improvements in annotation quality, cross-species comparisons, and the overall depth of the manuscript. We have broadened the analytical strategy, provided additional comparisons to multiple insect systems, revised the cell-type annotation using a more stringent multi-evidence framework, and deepened the differential expression analysis. We address each major and minor comment systematically below.

Major Comment 1: Dataset choice for cross-species mapping

The use of the Davie et al. 2018 Drosophila dataset for cross-species cell-type mapping is unfortunate. This dataset is shallow compared to more recent atlases, and fails to reliably identify certain key neuronal populations. Data from Park et al., 2022 for the central brain and Konstantinides et al., 2018 or Ozel et al., 2020 for the optic lobes would have been more suitable.

We thank the reviewer for this important recommendation. We have now performed a comprehensive, systematic SAMap analysis against each of the specifically suggested datasets — Park e*t al.* 2022 (central brain), Ozel e*t al.* 2020 (optic lobes) alongside the original Davie *et al.* 2018 (whole brain) — as well as three additional insect brain atlases: the *Ae. aegypti* brain (PMID: 41172998), the ant brain (PMID: 35711063), and the honeybee brain (PMID: 40199930). This six-dataset multi-species SAMap framework represents a fundamental upgrade to our annotation strategy. Crucially, using datasets with varying "cellular resolution levels" — from the fine-grained *Drosophila* atlases to the broader mosquito, ant, and bee datasets — allowed us to triangulate cell-type assignments with far greater confidence than any single reference could provide. Clusters that received convergent annotations across multiple independent datasets were assigned high-confidence identities (*e.g.* Fig. 1C); clusters with discordant or weak cross-species alignments were deliberately annotated more conservatively, with their top marker genes explicitly listed in the updated Table S2. The results of all pairwise SAMap comparisons are presented in Fig. 1 and Fig. S3, and a new consensus annotation incorporating all evidence streams is presented in Fig. 1 and Table S2.

Major Comment 2: Rationale and value of spatial transcriptomics

The rationale for including spatial transcriptomics is unclear, and the resulting data does not contribute meaningfully to this study. The analysis mostly confirms expected anatomical patterns, for instance, photoreceptor clusters appearing in the optic lobe region, with little new insight. The resolution is too low to accurately resolve relevant structures, making several conclusions from this experiment unreliable.

We respectfully maintain that the spatial transcriptomics data make a substantive and novel contribution to this study, and we have strengthened this argument in the revised manuscript. While mosquito brain anatomy has been described previously, this work was performed almost exclusively in *Ae. aegypti* — a species estimated to have diverged from *Anopheles* approximately 179 million years ago (PMID: 37803007). Dedicated studies on brain anatomy specific to *An. gambiae* are exceptionally sparse. Our Slide-seq data provide the first spatially resolved molecular map of a mosquito brain, generating untargeted spatial validation of conserved markers and offering an independent, orthogonal line of evidence that strengthens the robustness of our single-cell atlas. The spatial data confirm the broad anatomical organization of the mosquito head, validate cluster assignments for well-defined structures (e.g., optic lobe cell types), and, critically, reveal spatial patterns not predicted *a priori* from the single-cell data. We have significantly expanded the rationale for this experiment and its interpretive value in the revised manuscript, and we believe the spatial component now makes a more clearly articulated and justified contribution.

Major Comment 3: Annotation errors and ortholog assignments

The cluster annotation contains several major issues that question the reliability of the entire analysis, with several orthologue assignments between fly and mosquito being plainly incorrect. For example, AGAP000585 is a more likely orthologue of CG6118 than mamo. And even if it was a real mamo orthologue, mamo is expressed in both γ and α'β' Kenyon cells in Drosophila, so this gene cannot be considered a specific α'β' marker. Similarly, AGAP006107, not AGAP029446, is the trio orthologue in Anopheles.

We thank the reviewer for this meticulous scrutiny of our ortholog assignments, which motivated a formalization of our entire orthology pipeline. Regarding AGAP000585 (submitted manuscript, page 12, lines 344-345): this gene is a reciprocal BLAST top hit with the highest coverage against *D. melanogaster mamo* (e-value: 4E–71), is syntenic, and is classified in the same ORTHOMCL orthogroup as *mamo*. We could find no supporting evidence for AGAP000585 being an ortholog of *CG6118* despite both proteins sharing a BTB domain. AGAP000585 is a marker of Ag28, and our new multi-species SAMap results identify Ag28 as a Kenyon cell (KC) cell subtype (also matching an *Ae. aegypti* KC cluster for which *mamo* was independently identified as a major marker (Fig. 1C, Table S2). However we agree with the reviewer that AGAP000585 is not specific to α′β′ KC. Therefore, we have revised the annotation of the KC cell types to be more conservative and robust (page 12, lines 335-368). Regarding AGAP006107 and AGAP029446: these are alias IDs of the same gene, which is indeed the *trio* ortholog. We recognize that this ambiguity is unfortunate. In the revised manuscript, we have included a detailed, explicit description of our orthology determination strategy in the Methods section. We applied this rigorous framework to all marker genes discussed in the manuscript to ensure the integrity of every cross-species claim.

The gene Bx (AGAP000731) is absent from α'β' Kenyon cells in Drosophila, contradicting the proposed annotation of cluster 28. These errors indicate a lack of biological validation throughout

the whole manuscript. While the proposed Kenyon cell clusters 14, 28, and 32 are plausible, the supporting evidence for their subdivision into αβ, α'β', and γ types is not credible.

We thank the reviewer for this important correction. In the revised manuscript, we have substantially reworked the KC annotation to be both more precise and more conservative. While *Bx* is not detected in α′β′ KCs in scRNA-seq studies (PMID: 29671739), we note that the gene is present in a subset of α′β′ KCs as well as in αβ and γ KC at the protein level, where it plays a role in 24-hour long-term memory formation (PMID: 27841260, Fig. S7A). We have nonetheless substantially reduced our reliance on single SAMap-based subtype assignments for the final annotation. Ag28 is now conservatively annotated as a KC cluster without further subtype specification, consistent with the multi-species evidence available (page 12, lines 344-368). More broadly, in the new annotation framework, definitive subtype assignments are only made when convergent evidence from multiple independent datasets, multiple marker genes, and functional gene expression patterns collectively support a specific identity. Where this bar is not met, we explicitly state the uncertainty and list the supporting markers without overinterpreting the assignment.

Major Comment 4: Memory-relevant genes in Kenyon cells

The absence of several memory-relevant genes in KCs, including Dop1R1 as well as elements of memory-relevant signal transduction such as rut and PKA subunits, is a concern. The authors should discuss what consequences the absence of these crucial genes might have on how mosquitoes learn.

We thank the reviewer for directing attention to this question. We have now performed a systematic analysis of memory-relevant gene expression across all KC clusters. We find that the key memory signaling components are indeed detectable in our data. The *Dop1R1* ortholog AGAP004613 is a positive marker of ten clusters in our atlas, including all three KC clusters (Ag14, Ag28, and Ag32). The *rut* ortholog (*adcy8*; AGAP002262) is among the top 20 markers of all three KC clusters (Table S3, Fig. S9C and E). PKA subunit orthologs AGAP029637, AGAP006448, and AGAP004940 are all detected as markers of Ag14, Ag28, and Ag32. The identification of this complete core dopaminergic memory signaling module — *Dop1R1*, *rut*, and PKA subunits — in the *An. gambiae* KC clusters, strongly supports the conservation of associative learning machinery in this malaria vector. We have incorporated a substantive new discussion of these findings and their implications for mosquito learning behavior in the revised manuscript (page 12, lines 347-357; and page 18, lines 554-561).

Major Comment 5: OA neuron cluster (Ag7)

The OA neuron cluster (cluster 7) is also not convincingly identified. Although this cluster expresses Vmat, it lacks octopaminergic markers such as Tbh and Tdc2; and Dh31 is not an OA neuron marker in flies. The reported number of OA neurons is also inconsistent with expected numbers from Drosophila. Cluster 7 likely contains a mixture of cell types, including multiple peptidergic types, and therefore must not be reliably called octopaminergic.

After careful re-evaluation, we fully agree with the reviewer's assessment. The expression of the key octopaminergic markers — the *tdc2* and *tbh* orthologs, as well as the *DAT* and *ddc* orthologs — is predominantly confined to subsets of Ag7 rather than being expressed broadly across the cluster, alongside a wider expression of the Drosophila *VMAT* ortholog (*AGAP004476*). This expression architecture is more consistent with a heterogeneous monoaminergic cluster than a pure octopaminergic population. Furthermore, two top marker genes of Ag7 are the *Drosophila Dh31* ortholog (a neuropeptide hormone) and *amon* (a prohormone convertase) and are broadly expressed in the *Anopheles* brain (figure below). This is indicative of active neuropeptidergic

signaling. Taken together, the molecular profile of Ag7 most robustly supports a general monoaminergic and peptidergic identity with strong signs of active neurotransmission, and we have revised the annotation accordingly (page 13, lines 396-399; Fig. 1, Fig. S3A, Fig. S12).

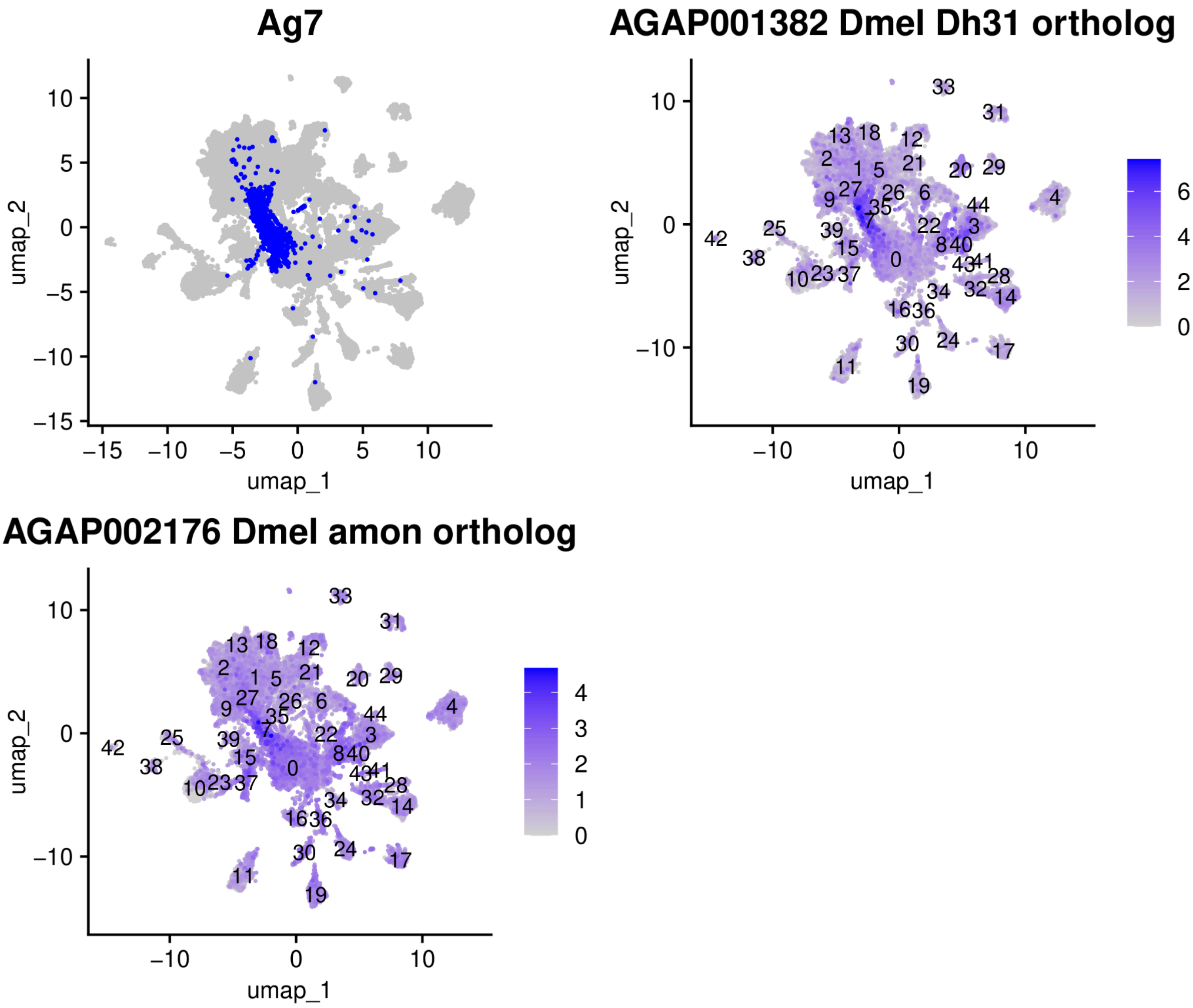

Major Comment 6: LNv clusters and clock neurons (An37/An18)

The possibility that clusters 37 and 18 could represent LNvs is inconsistent with expected cell numbers: flies have only 16 LNvs, and a sudden expansion in mosquitoes seems unlikely. Instead, these clusters likely represent a mixture of cell types, including possibly a minority of LNv cells. Related to this, statements such as “LNv cellular bodies have a spatially restricted distribution in the Anopheles brain” are trivial – aren’t all cell bodies spatially restricted?

We agree with the reviewer's concern. Indeed, only ~ 40 LNv neurons are thought to be present in the *Anopheles* brain (PMID: 32619483), a number inconsistent with assigning entire clusters this identity. In the original version, Ag37 and Ag18 were annotated as CCAP/clock neurons with LNv being the top SAMap match on the Davie dataset; we have now more explicitly differentiated the final annotation from top SAMap assignments in our multi-species comparison to avoid this ambiguity (Fig. 1 and Table S2). In the revised annotation, we restrict the clock neuron designation to Ag37 alone, supported by a convergent body of evidence: GPRCAL3 (the *Drosophila pdfr* ortholog) is the third-ranked top marker of Ag37 (Table S2); *pdh*-expressing (*pdf* ortholog) neurons are confined to a subset of Ag37, consistent with the expected low numbers

(figure below). Additionally, this subset expresses *cycle* (AGAP005655), a gene also expressed in glia, which is consistent with previous knowledge in *Drosophila* (PMID: 38263460). This multi-marker consensus provides a high-confidence annotation for Ag37 as the clock neuron cluster (pages 9-10, lines 295-313). We have also corrected the phrasing around spatial restriction of cell bodies as pointed out by the reviewer.

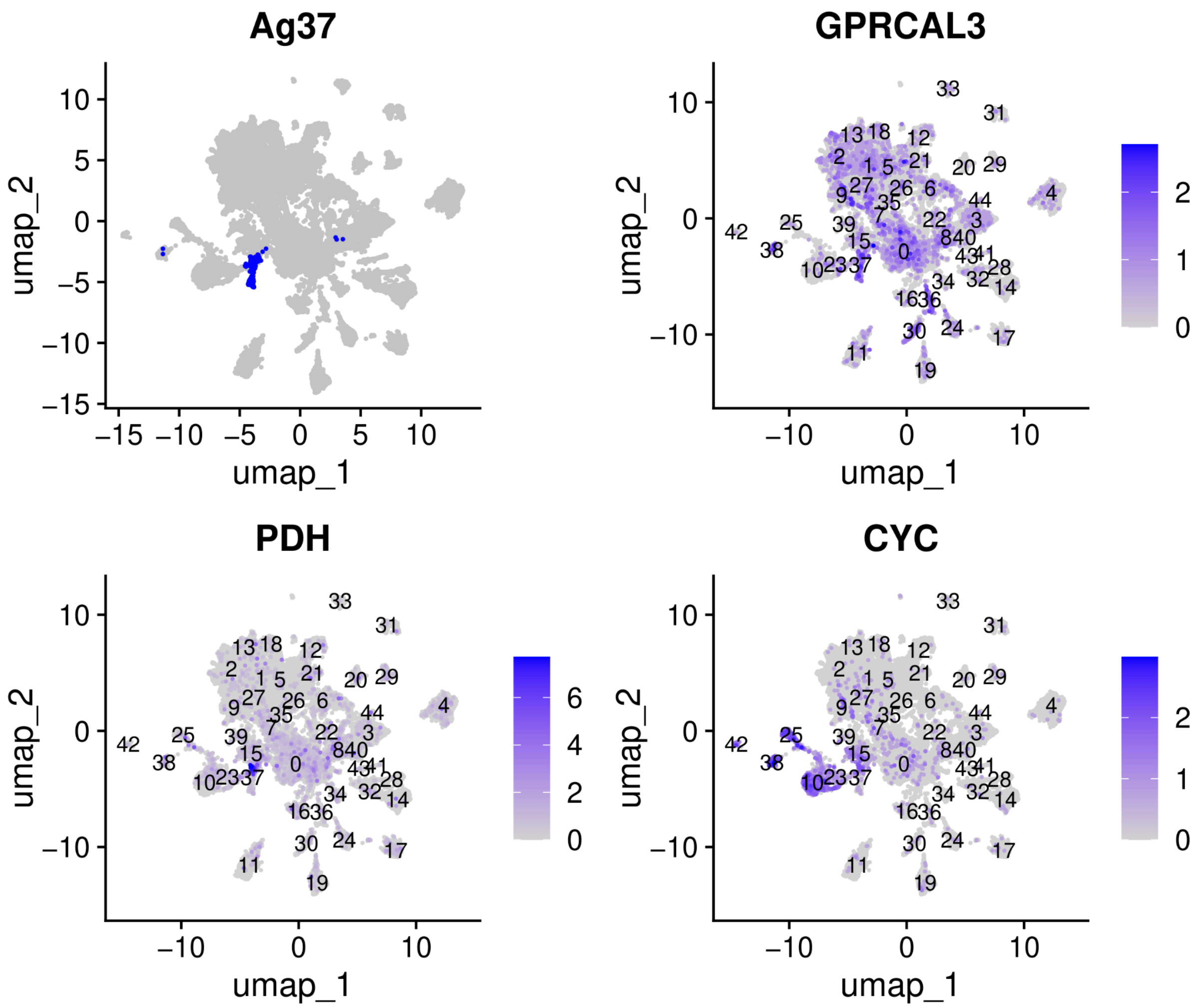

Major Comment 7: Mating and blood meal analysis

The analysis of mating and blood feeding effects is interesting but unfortunately represents a too small portion of the study, and lacks sufficient validation or further exploration of the results. Crucially, one could argue that mosquitoes maintained on sugar-only diets for 6 days after mating are effectively protein-deprived, raising questions about whether the observed transcriptional effects reflect genuine blood meal-induced physiological transitions, or simply the fulfilment of protein requirements. Better controls would include a blood meal immediately after mating, or providing a protein-supplemented sugar solution instead of blood.

This critique motivated one of the most substantial new analyses in the revised manuscript. We have now performed a full single-cell DEA across all three experimental conditions (virgin, mated, and blood-fed), leveraging the power of our multi-condition atlas to identify cell-type-specific transcriptional reprogramming at unprecedented resolution (Table S7 and Table S8). This analysis revealed transcriptional changes associated with specific cell populations

across life stages that are completely invisible in bulk RNA-seq profiles — for example, post-mating transcriptional reprogramming in clock neurons (page 13, lines 422-429). The integrated comparison between bulk and single-cell DEA (pages 13-14, lines 404-463) demonstrated complementarity between the two approaches: for conditions inducing subtle changes (mated), scRNA-seq reveals cell type-specific signals masked in bulk data; for major perturbations (blood meal), both approaches converge on consistent, high-confidence differential expression signatures. These new results are presented in Figs. 5 of the revised manuscript.

Regarding the protein deprivation concern, there is no literature that supports that sugar-fed female *Anopheles* mosquitoes are in a state of nutritional deprivation. While blood feeding is an integral component of the mosquito reproductive cycle required for egg maturation, it is not essential for basal female metabolism. Sugar-fed females have life expectancies comparable to blood-fed females (PMID: 38166422, 38533352), indicating that blood feeding primarily drives a switch toward reproductive processes — resting, oocyte development, and oviposition — rather than recovery from a generalized nutrient stress state.

Minor Comments — Reviewer #1

Minor 1: Table S2 statistical information

Table S2 lacks essential statistical information (LogFC, p-values) for marker genes, making it impossible to evaluate marker strength without having to constantly refer to table S3.

We have integrated complete statistical information — log2fold change, adjusted p-values — into the new version of Table S2.

Minor 2: Unidentifiable clusters

The authors attribute unidentifiable clusters to "technological limitations of cell state capture, […]." They should better explain what they mean here. If they believe that their approach cannot capture cell states accurately, this should be addressed experimentally, rather than speculated.

The heterogeneity in cell capture efficiency is a well-documented property of single cell RNA-seq dissociation protocols, and is a technical characteristic of dissociation-based approaches shared across all published single-cell atlases (PMID: 32487174). We have now revised our statements and commented on the inter-species cluster similarity in the light of the new multi-dataset homology analysis (page 18, lines 549-533).

Minor 3: Fast-acting neurotransmitter characterization

The manuscript provides too little analysis of fast-acting neurotransmitters in individual clusters. A more detailed characterization would be helpful.

In full agreement with this suggestion, we have substantially expanded the neurotransmitter profiling analysis. A comprehensive heatmap integrating fast-acting neurotransmitter markers across all 45 clusters is now presented in the updated Fig. S3, with detailed commentary added to the main text (page 2, lines 97-99;page 13, lines 393-402). This expanded profiling provides an important functional annotation layer complementary to the cross-species SAMap assignments.

Minor 4: Ag2 olfactory projection neuron annotation

The statement "An2 was associated to 9 D. melanogaster olfactory projection neuron subtypes (Fig. S3) suggesting it is composed of cells of the antennal lobe" is inaccurate, since olfactory PNs are not uniquely localised in the antennal lobe: only their dendrites are.

We thank the reviewer for this comment. The annotation of Ag2 has been revised in the updated manuscript. As discussed below in response to Reviewer #2, we find that while Ag2 shows characteristics consistent with projection neurons, canonical OPN-specific markers from *Drosophila* studies show broad expression across multiple clusters in our data, precluding a specific OPN designation. The annotation has been updated accordingly (Fig. 1, Table S2).

Minor 5: *Plasmodium* infection discussion

"This could be due either to (i) a transcriptionally-silent parasite manipulation phenotype, (ii) subtle changes below the detection threshold (e.g., changes only in a small group of cells), (iii) an ineffective parasitic manipulation in our specific parasite-mosquito strains combination and lab conditions." These possibilities must be further explored to provide a valid explanation for the unexpected absence of clear gene regulation in the brains of Plasmodium-infected animals.

We have revised the discussion of this unexpected negative result which can be explained by (i) genuine absence of transcriptional manipulation in the tested conditions at the sampled time points and with our specific pathogen-vector combination; or (ii) manipulation is occurring but was undetectable here, either due to the cell-type specificity of the signal (requiring single-cell resolution to unmask) or because it operates at the post-transcriptional or proteomic level (page 18, lines 586-593).

Minor 6: Circadian phase

A mention of the circadian phase would be useful for all experiments, especially as transcription in clock neurons is being assessed.

We thank the reviewer for this essential methodological clarification. All brain dissections for both scRNA-seq and bulk RNA-seq experiments were performed in the same time window, between 8:30 AM and 11:00 AM, to minimize circadian variation. Three operators alternated dissections across experimental conditions to avoid systematic dissection-order biases. This information has been explicitly added to the Methods section (page 19, lines 638-639, recalled at page 21, lines 702-704; page 20, lines 673-675).

REVIEWER #2

We are grateful to Reviewer #2 for the positive assessment of the study's technical rigor and for a set of incisive recommendations that have substantially strengthened the manuscript. In direct response to the major suggestions, we have (1) performed and fully integrated a single-cell DEA across life stages, revealing cell type-specific transcriptional dynamics inaccessible to bulk approaches; (2) expanded our cross-species SAMap analysis to five additional datasets spanning four insect species; and (3) comprehensively revised our cluster annotation pipeline to maximize specificity and minimize annotation artifacts. Each point is addressed in detail below.

Major Comment 1: Single-cell DEA across physiological conditions

This work emphasizes transcriptional changes linked to mosquito life history events (mating, blood-feeding, Plasmodium falciparum infection, and age). The single-cell atlas is derived from different physiological states of individuals, but only bulk RNA-seq results are compared. The authors should clarify in the abstract and results that these life history comparisons are bulk-based, while explicitly addressing how they inform cell-type-specific interpretations (e.g., via mapping DEGs to the atlas clusters). More critically, the significance of these comparisons lies in their potential to reveal state-dependent shifts in cell-type composition or gene expression profiles—e.g., whether mating or blood-feeding alters neuronal subtype proportions, glial activation, or cluster-specific transcriptional states. Currently, it remains unclear how such

differences manifest at the single-cell level, limiting insights into dynamic neural remodeling during vectorial behaviors. The study could be strengthened by analyzing scRNA-seq data from virgin, mated, and blood-fed conditions, which would directly link bulk observations to cellular heterogeneity.

This recommendation has been fully implemented and represents one of the most significant analytical additions to the revised manuscript. We performed a comprehensive single-cell DEA across all three experimental conditions identifying differentially expressed genes at the individual cluster level (Table S7 and Table S8). This analysis revealed striking cell-type-specific transcriptional reprogramming that is entirely masked in bulk profiles. Key findings include differential gene expression in clock neuron cluster Ag37 following mating (page 13, lines 422-429) — a finding not captured in bulk RNA-seq — as well as widespread but heterogeneously distributed transcriptional responses to blood feeding across neuronal, glial, and immune cell clusters (pages 13-14, lines 404-463). The complementarity and divergence between bulk and single-cell DEA results are now explicitly discussed, with the key scRNA-seq findings highlighted in the abstract (page 1, lines 33-38). These results are presented in full in Fig. 5 and Fig. 6 of the revised manuscript.

Major Comment 2: Multi-dataset cross-species annotation

Accurate identification of cell types is paramount in this first-of-its-kind single-cell atlas of the Anopheles brain. The authors' use of SAMap for mapping to a Drosophila brain dataset is a solid foundation. However, the analysis relies solely on a single Drosophila brain dataset, which may limit resolution at the subcluster level, particularly for specialized regions. Incorporating additional Drosophila datasets (e.g., PMID: 33125872 and PMID: 33149298) would potentially resolve ambiguities in unannotated clusters and strengthen claims of Anopheles-specific adaptations. Furthermore, leveraging single-cell brain atlases from other insect species could broaden the comparative framework. For instance, integrating data from ants (PMID: 35711063) and honeybees (PMID: 40199930) would facilitate multi-species homology assessments and highlight mosquito-specific divergences. This would elevate the atlas's utility as a cross-insect resource without substantial new data generation.

We are grateful for this recommendation and have implemented it in full. In the revised manuscript, we present SAMap analyses against six reference datasets: (1) the Davie et al. 2018 whole *Drosophila* brain; (2) the Park et al. 2022 *Drosophila* central brain; (3) Ozel et al. 2020 *Drosophila* visual system; (4) the *Ae. aegypti* brain; (5) the *Monomorium pharaonis* ant brain (PMID: 35711063); and (6) the *Apis mellifera* honeybee brain (PMID: 40199930). The multi-atlas comparison proved very informative. Datasets with general, broad annotations (mosquito, ant, bee) and those with fine-grained subtype resolution (fly) complement one another: broad-annotation species provide high-confidence functional category assignments, while *Drosophila* datasets enable subtype disambiguation where evidence is sufficient. Triangulating across all six references substantially reduced annotation uncertainty and revealed several mosquito-specific divergences that could not have been identified from any single reference. These results are presented in the new Fig. 1 and S3 and Supplementary Table S1 and S2.

Major Comment 3: Annotation specificity — An2 and marker overlap

All downstream analyses, including spatial mapping, neurotransmitter profiling, and life history event integrations, hinge on the accuracy of cell-type annotations. I have several questions and concerns regarding specificity, particularly for olfactory and visual neuron subtypes. The evidence for the olfactory projection neuron identity of cluster An2 (labeled as adPN-lPN-OPN in Fig. 1C) is not fully convincing. While vacht is appropriately highlighted as a cholinergic marker, it

is not a canonical OPN-specific gene. In Drosophila, VAChT broadly denotes excitatory projection neurons but lacks subtype specificity for antennal-driven or inter/intra-projection classes. Moreover, vacht exhibits widespread expression across multiple clusters (e.g., An0, An4, An11, An12, An16, An17, An19, An20, An21, An22, An24, An28, An29, An30, An32, An34, An35; Table S1), diluting its utility as a defining feature for An2. Compounding this, Fig. S3 indicates high SAMap similarity of An2 to diverse Drosophila types, including Proc/Grob5 progenitors, suggesting potential overlap with precursor states rather than mature OPNs. To bolster this annotation, the authors should evaluate canonical OPN markers from Drosophila studies, such as oaz or GH146/acj6, assessing their expression in An2. If absent or low, consider reclassifying An2 as a broader cholinergic PN or uncommitted cluster.

Similarly, marker overlap complicates other assignments. For instance, drgx (AGAP008868) is proposed as a marker for Tm1/TmY8 (An16) and T4/T5 (An20) neurons (Fig. S8A, B), yet expression data show it in both clusters (Fig. S8B). However, SAMap aligns Tm1/TmY8 more strongly to An20 (Fig. S8A), casting doubt on An16's designation and risking misattribution of visual processing roles. Broader scrutiny of Table S1 reveals several such multi-cluster markers, which may reflect technical noise or annotation artifacts.

We have undertaken a comprehensive re-evaluation of all cluster annotations with particular attention to the issue of multi-cluster markers. For Ag2 specifically: while the cluster showed SAMap similarity to *Drosophila* OPNs and broad expression of cholinergic markers, we could not identify consistent expression of canonical OPN-specific marker genes (*oaz*, *acj6*/GH146, *cut*) from *Drosophila* studies (PMID: 29671739). These genes showed broad, non-specific expression patterns in our data, likely reflecting genuine evolutionary divergence in marker gene expression between *Drosophila* and mosquitoes rather than a technical artifact (figure below). In the revised manuscript, Ag2 is annotated as a neuron cluster, without specifying an OPN identity. More broadly, our revised annotation framework now requires that a marker gene show both statistical enrichment and expression sufficiently specific to be biologically informative before it is used to support a specific annotation. Clusters where no such markers exist are annotated by their top expressed genes without imposing a cell-type label (Fig. 1D, Table S2). This approach substantially reduces the risk of annotation artifacts while still providing maximum utility to the community.

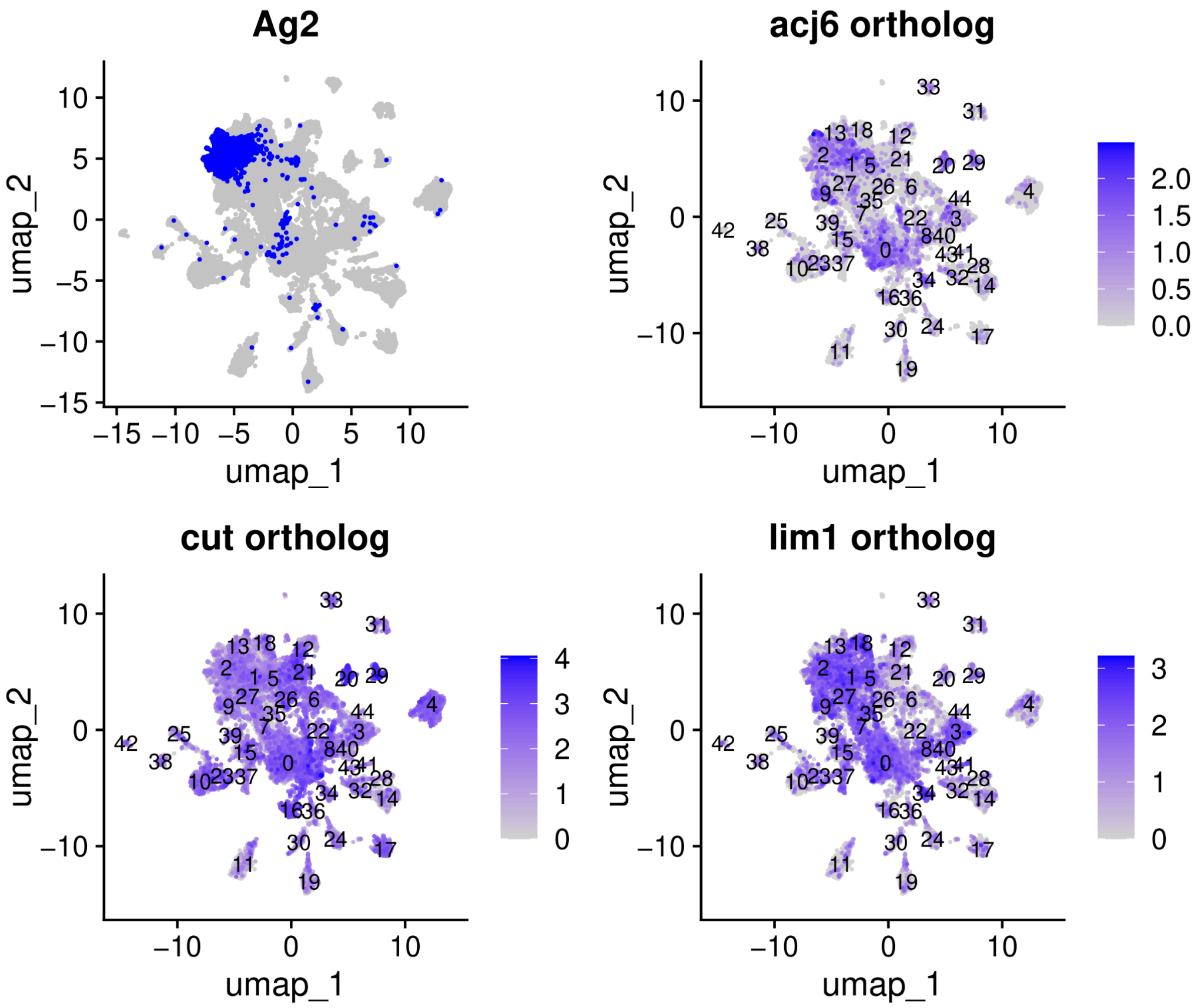

Minor Comments — Reviewer #2

Minor 1: Replicate numbers in figure legends

It would be helpful to include information on the number of replicates (N = #) in the figure legends.

Replicate number values have been added to the legends of main Figures 1, 5, and 6, as well as all relevant supplementary figures.

Minor 2: *Drosophila* ortholog names alongside *Anopheles* gene IDs

Displaying Drosophila homolog names alongside Anopheles gene IDs would greatly improve interpretability.

We have introduced *Drosophila* ortholog names throughout the revised manuscript: in Table S1, Table S2 (top 5 markers per cluster), and in the main text wherever specific gene functions are discussed.

Minor 3: Physiological state of spatial transcriptomics mosquitoes

The mosquitoes used for spatial transcriptomics are described as seven-day-old An. gambiae females, but their physiological state (e.g., virgin, mated, or blood-fed) is not specified.

The mosquitoes used for spatial transcriptomics were seven-day-old virgin *An. gambiae* females fed ad libitum on a 10% honey solution. This information has been explicitly added to the Methods section (page 19, lines 607-610; page 20, line 673).

Minor 4: Reproducibility of spatial patterns in Fig. 4D

In Fig. 4D, the authors describe gene expression patterns corresponding to the optic lobe (OL) layers. To support this interpretation, the authors could provide additional examples across multiple brain sections to demonstrate that the observed spatial relationships are reproducible across samples.

We thank the reviewer for this suggestion. Additional Slide-seq sections confirming the reproducibility of the optic lobe layering patterns are now presented in Figure S9. The consistency of gene expression spatial organization across independent brain sections supports the biological validity of the observed patterns.

Minor 5: Cell visualization with sparse single-cell data

Marker Gene Expression Analysis. Given the inherently sparse nature of single-cell data, randomly selecting 500 cells for visualization (Fig. S7B, S8B, S9C, S10B) may not fully capture the overall expression patterns. The authors could consider averaging expression levels across defined cell groups or performing repeated random sampling to assess the consistency of the observed trends.

We thank the reviewer for raising this methodological point. After careful consideration, we are confident that the heatmap representations faithfully reflect the underlying expression patterns. Our marker gene visualization relies exclusively on genes that pass a stringent expression threshold (pct ≥ 25% of cells in the cluster, as identified by FindAllMarkers), ensuring that displayed patterns are not driven by dropouts or sparse sampling artifacts. In support of this we have performed the averaging that the reviewer suggests and have noticed consistent patterns. The pct values for all displayed markers are reported in Table S3 for independent verification. We have added this information explicitly to the Methods section (page 23, lines 825-827).

Minor 6: Methods section detail

The Methods section needs more precise descriptions. The current protocols lack many details.

We have substantially expanded the Methods section throughout, adding detailed descriptions of: mosquito dissection timing and operator rotation procedures (page 19, lines 606-640); the SAMap implementation including parameters, datasets and subsets (healthy females where possible) used, and alignment score interpretation (page 23, lines 830-848); the orthology assessment (page 23, lines 849-851); the Seurat integration workflow (pages 22-23, lines 815-824); the DEA framework for single cell data (page 23, lines 828-830) and the Slide-seq data processing pipeline (page 23-24, lines 852-890).

REVIEWER #3

We are grateful to Reviewer #3 for the positive assessment of this work and for a comprehensive and constructive set of recommendations. The resulting revision is substantially improved in terms of scholarly framing (introduction), interpretive depth (discussion), analytical rigor (annotation, DEA), and data accessibility. We address each point below.

Major Comment 1: Introduction expansion

Currently the introduction lacks scholarship and the importance of this work. To make this paper more suited for a general reader, it should be expanded. Some information that can be added: What was previously known in the literature, and what was previously done. How can a combination of single-cell sequencing, spatial transcriptomics, and bulk RNA sequencing inform new insights and expand on what they did and find? What is the importance of this study?

We have substantially rewritten the Introduction to better frame the importance and novelty of this work for a broad readership, whilst trying to stay synthetic to remain within the word limit. The revised introduction now provides: (1) an overview of what was previously known about insect and mosquito brain anatomy and transcriptomics (page 1-2, lines 45-60); (2) a clear articulation of what remained unknown especially in malaria vectors (page 2, lines 60-61, 68-71). The implications of this resource for vector biology, malaria control, and comparative insect neuroscience have been discussed (Discussion, page 17-19, lines 530-602).

Major Comment 2: Pan-neuronal markers — elav-like validation

Annotation of all neurons is a major part of the single-cell brain atlas. The authors write that "...expression of cellular markers for neurons (elav-like) and glia (repo) as these markers are widely conserved across the animal kingdom 15,16, including in dipterans 13." Is there direct evidence of elav-specific and pan-neuronal expression in the adults An. gambiae? If so, please cite. If not, did the data reveal other pan-nuronal markers that can be used to classify neurons (brp/Syt1/nSyb see: PMID: 34590074, PMID: 35239393, PMID: 40060408), and why weren't they used?

We thank the reviewer for raising this important point. To our knowledge, this study provides the first demonstration that elav-like (AGAP000965, an ortholog of *D. melanogaster fne* and *rbp9*) functions as a pan-neuronal marker in *An. gambiae*. The ELAV/RBP gene family (*elav*, *fne*, *rbp9*) is predominantly pan-neuronal across metazoans (PMID: 35429136; PMID: 27802174), and we originally assessed AGAP000965 as the most broadly and consistently expressed family member across our neuronal clusters, making it the most appropriate choice for pan-neuronal classification. Following the reviewer's suggestion, we additionally assessed the expression patterns of the *Ae. aegypti* and *D. melanogaster* orthologs of *Syt1*, *nSyb*, and *brp* in our data. *Synaptotagmin-1* and *nSyb* show expression patterns broadly comparable to AGAP000965 (figure below), providing independent validation of our neuronal cluster assignments. We also characterize the expression of the *brp* ortholog AGAP010286. These additional pan-neuronal markers have been incorporated into Fig. 1 and added to Table S1, substantially strengthening the foundation of our neuron-glia classification.

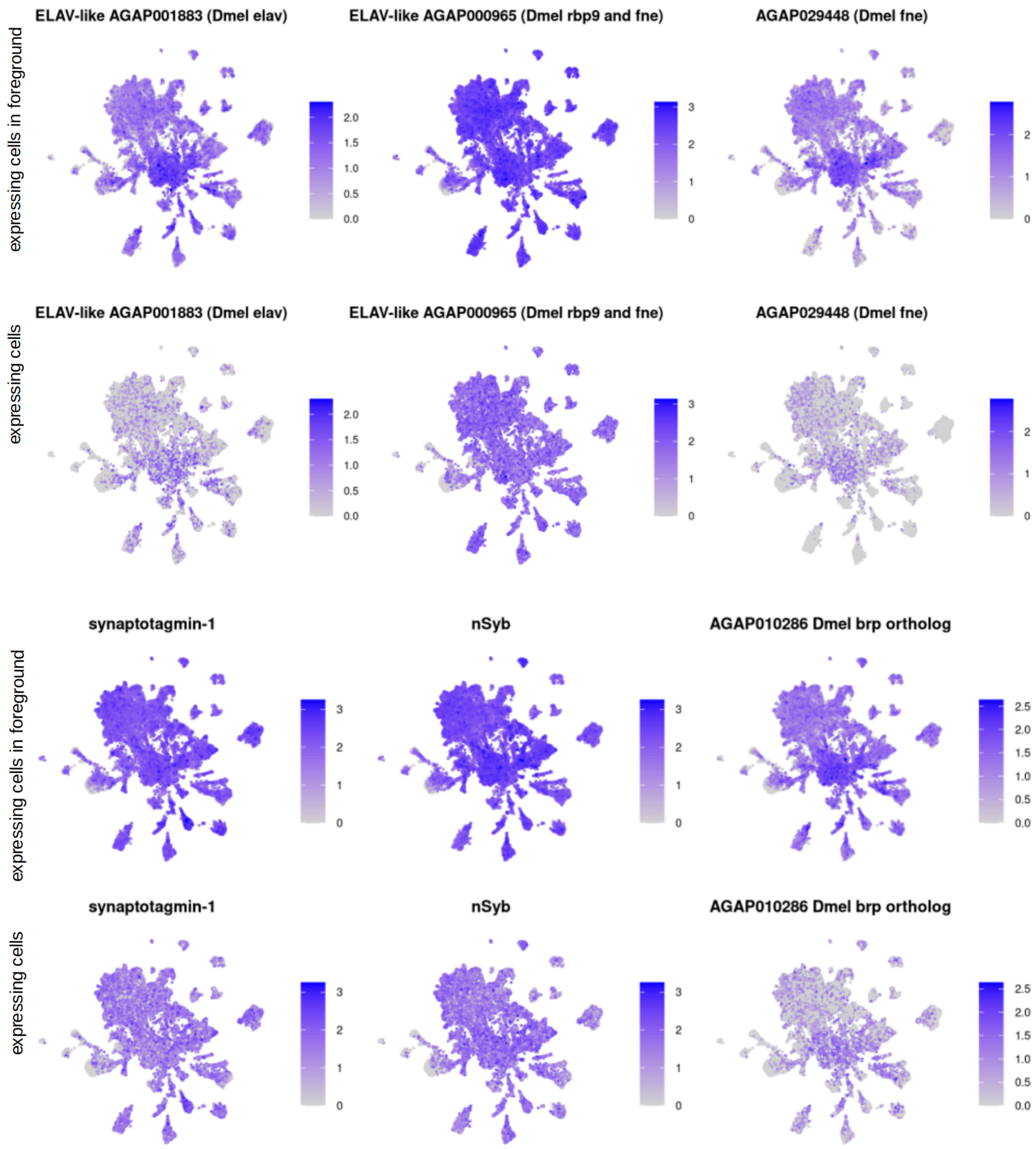

Major Comment 3: Batch effects across experimental conditions

*The annotation of the single-cell brain atlas combines six 10x runs from three biological conditions (virgin, mated, blood-fed). While increasing cell numbers enables enhanced annotation power, combining different conditions may introduce batch effects or technical and biological variability. Did the authors use an approach to check and account for these effects across the six pools? Please clarify in the main text or methods. Also, explicitly state in the main text and figure legend that the atlas merges data from multiple condition*s.

To control for batch effects, we used three approaches (i) 2 replicate single cell pools per condition (ii) controlling for rearing batch effects (iii) using vetted batch correction analysis. Specifically, we have integrated the information on breeding conditions and handling procedures applied to avoid rearing batch effects in the Materials and Methods section (page 19, lines 607-617, and lines 638-639, recalled at page 21, lines 702-704; page 20, lines 673-675). During data analysis, we applied Seurat's dataset integration workflow to correct for technical batch effects introduced during sample dissociation and library preparation across the six 10X runs. This procedure is specifically designed to identify cell populations consistently present across all samples while preserving biologically meaningful variation. Following integration, we verified that

cells from all six runs and all three experimental conditions are uniformly distributed across all clusters (Fig. S2E and F), confirming that clustering is driven by cell identity rather than technical or condition-specific batch effects. We have deepened the description of the scRNA-seq data processing, including the parameters used and the validation strategy, to the Materials and Methods section (page 23, lines 819-821; page 24, lines 897-899), and explicitly note in the main text that the atlas integrates data from three experimental conditions (page 2, lines 81-82).

Major Comment 4 & 5: Single-cell DEA and integration with bulk results

The section titled "Life history is associated with distinct brain transcriptional states" in the paper is the weakest and should be reanalyzed. First, there is currently no analysis presented for the single-cell brain conditions (virgin, mated, blood-fed), and it isn't clear why those experimental conditions were performed. Instead, this section is focused on single brain bulk RNA seq. Was there any cluster-based differential analysis (MAST or any other) performed between these conditions in the single-cell data? If such analyses were performed, did they identify differentially expressed genes at the cluster or cell-type level? Clarifying whether these fundamental analyses were done is essential, as they are at the base of the single-cell experiments. Do the differential analysis results from the single-cell analyses align with the findings from the bulk RNA data in Figure 5? If not, what are the observed differences, and what might explain these discrepancies? Clarify whether and how these datasets agree or diverge.

The single brain bulk RNA-seq aims to link virgin, mated, and blood-fed conditions to gene expression changes. Using a single brain reduces variation and was well executed. However, how can cell types be reliably linked to bulk sequencing results (Figure 5B, E, and J)? Did any computational deconvolution analysis to estimate cell type proportions or changes in cell type-specific expression across conditions using the single-cell atlas was performed? Please clarify this in the main text and methods. Additionally, discuss the substantial limitations of such analysis in the main text and discussion.

This is addressed by one of the most substantial new additions to the revised manuscript. We have now performed a full cluster-level single-cell DEA across all three experimental conditions that accounts for within-condition variability and is robust to the statistical artifacts that can affect cell-level tests. This analysis revealed clear cell-type-specific transcriptional programs associated with mating and blood feeding that are either absent or attenuated in bulk RNA-seq data. Notably, mating-associated transcriptional changes are preferentially detected in specific neuronal clusters (including clock neurons) in the single-cell data (page 13, lines 422-429) despite being below the detection threshold of bulk RNA-seq, underscoring the power of single-cell resolution for detecting subtle but cell-type-specific regulatory events. In contrast, blood-meal-induced DEGs show substantial expression trends concordance between bulk and single-cell datasets, validating both approaches for major transcriptional transitions (pages 13-14, lines 404-463). A fully integrated comparison of bulk and scRNA-seq DEA results, including discussion of convergence and divergence, is now presented in the revised Results (Fig. 5) and Discussion sections (page 18, lines 562-585).

Major Comment 6: *Plasmodium* infection discussion

Given the lack of transcriptional changes after P. falciparum infection, how do the authors interpret these results in the context of existing literature? What possible explanations, such as sample timing, sample size, or other factors, might account for these findings? Further discussion of the implications and how these results relate to previous work should be added.

The question of behavioral manipulation by *P. falciparum* remains a pivotal one that has yielded variable results in the literature. We envisage two main scenarios: (i) genuine absence of transcriptional manipulation in the tested conditions and with our specific pathogen-vector combination; or (ii) manipulation is occurring but was undetectable here, either due to the cell-type specificity of the signal (requiring single-cell resolution to unmask) or because it operates at the post-transcriptional or proteomic or metabolomic level. Although we considered performing single-cell sequencing in the infection context, the biosafety containment requirements for *P. falciparum*-infected mosquitoes make this technically and logistically challenging. We have included a paragraph in the Discussion to address these hypotheses and to outline how our atlas could serve as a reference framework for future targeted studies designed to distinguish among them (pages 18, lines 586-593).

Major Comment 7: Limitations of bulk RNA-seq for complex transcriptional responses

Finally, this work shows the complexity of the mosquito brain, using both single-cell RNA sequencing and a spatial transcriptomics approach. Given these advancements, the use of bulk RNA sequencing alone may be insufficient to capture the full complexity of transcriptional responses to mating, blood-feeding, or infection in the mosquito brain, potentially missing critical cell-type-specific or spatially localized changes and should be clarified and further discussed in the paper.

We fully agree with the reviewer's comment. We have now integrated both bulk and scRNA-seq data for mosquito life stages DE analyses to take advantage of the complementary strengths of the two techniques in the context of this study. More precisely, we leveraged: (1) the power of bulk RNA-seq for detecting high-magnitude, whole-organ transcriptional shifts (*e.g.*, nutrient-guided blood meal response); and (2) the essential role of single-cell DEA for resolving cell-type-specific and subtle condition-dependent changes. We recognize that both approaches have limitations in capturing fragile cell types, rapidly transient states, or spatially restricted expression changes (discussed above in major comments 4 & 5 of Reviewer 3 and in minor comment 2 of Reviewer 1).

Major comments 8 & 9: Discussion expansion and comparative neurobiology

The discussion part of this paper is practically missing, while the authors added a conclusion of their work; discussing their findings is crucial to understanding their importance. This part may include a comparison to recent literature, hypotheses about the gene families and specific genes found in Figure 5, and the importance or implications that the work might have. In addition, as this work is focused on computational analysis, a limitation of those experiments and analyses should be discussed. Other discussion points are suggested in some of the points raised.

The paper will benefit from deeper discussion of mosquito-specific neurobiological features and comparisons within Diptera. The authors should discuss their findings in context of other mosquito brain studies, such as PMID: 36277452, PMID: 40060408, PMID: 37182106.

The *Discussion* section has been expanded in the revised manuscript. The new *Discussion*: (1) contextualizes our cell-type annotations and mosquito-specific adaptations within recent literature on insect brain organization, including PMID: 36277452, PMID: 40060408, and PMID: 37182106 as specifically recommended; (2) provides detailed interpretation of the DEA findings in the context of mating-induced reproductive transitions and blood-meal-driven physiological reprogramming; (3) addresses the implications of our results for understanding mosquito memory, learning, and vectorial behavior (4) expand the discussion of our result on the lack of observable manipulation in our experiments.

Minor Comments — Reviewer #3

We have addressed all minor points raised by Reviewer #3 as follows:

Minor 10: Title

Please change the title of this atlas to "The Anopheles gambiae female mosquito brain at spatial and single-cell resolution". As anopheles research advances, it would be beneficial to know which species and sex the paper refers to.

We have updated the title to “The brain of the female *Anopheles gambiae* mosquito at spatial and single-cell resolution” incorporating species and sex as recommended.

Minor 11: Nomenclature consistency

Similarly, there is an inconsistency in using Anopheles throughout the paper and figure titles. In some places, the authors are using only Anopheles, and in other places, An. gambiae (and other times An gambiae). As this work relates only to Anopheles gambiae, the authors should state the species name everywhere and spell it “An. gambiae”.

All instances of “*Anopheles*” have been standardized to “*An. gambiae*” throughout the manuscript, figures, and tables with the exception of when we discuss genus level aspects.

Minor 12: CNS usage

The use of CNS/central nervous system in the results of this work is misleading, as the work profiles only the brain and doesn’t touch any parts of the ventral nerve cord or other ganglia.

All instances of “CNS/central nervous system” that referred exclusively to the brain have been corrected to “brain.”

Minor 13: Pool/batch definitions

The first paragraph in the results (and Figure S1A) mentions 6 pools - it is not clear which pools are being referred to. Is a pool a single 10x run? Please clarify in the text. In addition, Figure S1A/C states that 2/3 batches were used. Please clarify what batches are.

We have added explicit definitions of “pool” (10 brains from one experimental condition, one 10X run) and “batch” (one adult emergence event from a single egg-laying batch) to the Fig. S1 legend and Methods, and further specified numbers of samples and individual brains used in Fig. 1, Fig. 5 and Fig. 6.

Minor 14: Fig. S1 legend

Figure S1 visualization is informative and clear. However, it lacks an appropriate figure legend to help the reader follow the experimental workflow without turning to the method section.

The experimental workflow legend has been substantially expanded to allow interpretation without reference to Methods.

Minor 15: Table S1 citation numbers

In Table S1, the brackets after the references do not correlate to the correct citation number and should be fixed.

Reference bracket numbers have been corrected throughout Table S1.

Minor 16: Fig. 1A pie charts

In Figure 1A, the pie charts can use more information on them - how many clusters they represent and how many total cells each section represents.

Fig. 1A has been redesigned in the context of the new multi-species analysis; cluster size information is now presented in Fig. S2.

Minor 17: Tilde in Fig. 1A

In Figure 1A legend, there is a tilde (~) next to the 48,806 cells - is this an estimated number?

The approximate symbol has been removed; the exact cell count is now stated.

Minor 18: QC metrics

QC matrices for the single-cell atlas should be more readable and should include more information (how many cells were collected per sample?). In Figure S2, the violin plots aren't readable - using a log scale will enable showing the distribution better. In addition, the average numbers (both total and per sample) should be noted on the figure/legend or in a separate supplementary table to enable comparison.

Log-scale violin plots and per-sample cell count statistics (median) have been added to Fig. S2.

Minor 19: SAMap documentation

The authors used SAMap to compare their data to a fly brain data set to help with annotating their cluster. This should be explained in detail in the method section, including the strategy and parameters used, and which of the data from PMID: 29909982 was used. In addition, they need to explain if and how differences in the data set were evaluated (for instance, sample size, male fly brains).

A detailed description of the SAMap implementation — including software version, parameters, dataset subsets (healthy females where possible), and alignment score interpretation — has been added to the Methods (page 23, lines 830-848).

Minor 20: Brain structural differences

In the "Spatial organisation of the female An. gambiae head" results, the authors write "However, we noted minor differences compared to the Drosophila brain, such as the different substructures of the central complex, the number of antennal glomeruli, or the different lobes of the mushroom body (Fig. S4)". The authors should expand on the differences they noted.

We have expanded the discussion of *An. gambiae*-specific brain structural differences from *Drosophila*, including differences in central complex substructures, antennal glomerulus number, and mushroom body lobe organization (Fig. S4 and main text, page 3, lines 137-147).

Minor 21: Glial marker co-expression

The functional roles of identified glial subtypes in Anopheles gambiae mosquitoes lack direct experimental validation. This is particularly important given the unexpected co-expression patterns of markers that are mutually exclusive in Drosophila, suggesting potential divergence in glial cell functions. While molecular signatures suggest conservation of glial functions, direct experimental validation will be needed to assess if those observations are correct. I don't think that there is need for additional experimental validation, in this paper, however the authors should acknowledge and discuss this in the text.

We have added an explicit discussion acknowledging the unexpected co-expression of mutually exclusive *Drosophila* glial markers in *An. gambiae* clusters, noting this as evidence of potential glial function divergence and highlighting the need for experimental validation (page 7, lines 234-243; page 17, lines 534-541).

Minor 22: Spatial organization of vision/clock genes

Similarly, the authors should discuss how the spatial organization of the vision-associated and clock genes aligns with insect brain regions known to be involved in vision and circadian rhythms. Acknowledge the need for functional studies as a key future direction.

We have added discussion contextualizing the spatial distribution of vision-associated and circadian gene expression relative to known insect brain anatomy (Fig. S9), and explicitly identified functional validation as a key future direction (page 18, lines 564-574).

Minor 23: Fig. 2A arrow visibility

In Figure 2A, the white arrow isn't visible and should be changed to a more visible color.

The white arrow has been replaced with a high-contrast color for visibility.

Minor 24: Fig. 4E/F clock genes in scRNA-seq

In Figure 4E and F, the data describe the localization of identified clock genes. Was this seen in the single-cell data? How does the expression of those genes look? and can you use it to identify clock cells outside cluster An37?

AGAP000955 and *ast2* are confirmed as markers of Ag37 in our single-cell data (Table S3) and have been added to the marker heatmap in Fig. S8.

Minor 25: Data accessibility

Data accessibility:

As both the raw sequencing data and Github page were not available at the time of this review, I couldn't assess them.

To increase data accessibility and to allow mosquito researchers easy visualization, the annotated brain single-cell data (including condition information) should be upload to one of the freely available visualization tools like: SCope, ASAP or UCSC Cell Browser.

We apologise for this oversight, the deposited data and code have now been made public. Raw sequencing data are now public at ENA (PRJEB90373). The GitHub page is now public (https://github.com/Parasitedevbio/Agambiae_Brain_Atlas.git). For broader accessibility, the data and visualisation is in the process of being made available via Vectorbase with a cellxgene solution.

Minor 26: Bulk RNA-seq data table

In addition, adding a supplementary table of gene expression from all bulk RNA-seq data (as TPM or other normalized counts) will allow researchers easy access to the data (similar to what was uploaded to the drive.ird.fr).

These data as well as further count matrices and meta data are now available online (https://drive.ird.fr/s/Lr4trcwLWasZpNK) and referenced on the Github repository as well.

Minor 27–30: (Typographic/formatting corrections)

In the method section, mosquito sample conditions - "FigS1A" should be Fig. S1A.

In Figure S3 legend, there is a space missing after the word each, "eachD. melanogaster" should be each D. melanogaster.

In the "A spatially-resolved map of cellular diversity in the An. gambiae brain", Glia section, the gene threonine 3-dehydrogenase (AGAP011948) is missing the gene name thr3DH that appears on the figure- should be threonine 3-dehydrogenase (thr3DH; AGAP011948).

Currently, the text mixes American and British English spellings for example, we both "behavior" and "behaviour," "organization" and "organisation," and "color" and "colour"are spelled in different sections. This should be consistent throughout.

All identified typographic and formatting errors — figure reference formatting, missing spaces, gene name inconsistencies, and British/American English mixing — have been corrected. The entire manuscript is now consistently written in American English.

REVIEWER COMMENTS

Reviewer #1 (Remarks to the Author):
The manuscript has improved substantially. The updated cell type annotation is now much more accurate and the added analyses on various cell types and fast-acting neurotransmitters make the study more complete. The section about life history is also improved, even if it feels like this section could stand as one or several separate studies.
Answer to Reviewer's 1 remarks
We are glad we could address most of the Reviewer's comments satisfactorily, and want to renew our gratitude for the usefulness of their feedback.

However, the following concerns remain:
1. A few issues in terms of cluster annotation and choice of marker genes. First, given the diversity of genes expressed in Ag7, I recommend that the authors annotate it as "monoaminergic and peptidergic" rather than purely monoaminergic. Further, even though it appears as a marker of Ag7, Dh31 is not an appropriate marker for monoaminergic neurons, and Vmat should be used in Fig S12 to identify these cells instead. Regarding Ag37, expression of key markers is not sufficiently uniform to justify naming the whole cluster "clock neurons". I would recommend a more careful annotation (e.g., "clock neuron enriched").
Answer to Reviewer 1, comment 1.
Following the Reviewer suggestion, we have updated Ag7 and Ag37 annotations in both Fig. 1, Fig. S3, text and figure legends (lines: 413, 416, 561, 901, 1341, 1467). We have now also removed *Dh31* and included the expression of the *vmat* ortholog in Fig. S12.

2. I am not convinced by the authors' response to my comments regarding the spatial transcriptomics data, which focuses mostly on the novelty (which I recognise) but brushes off my concerns about the limited resolution and usefulness of this dataset. Importantly, Fig S5E suggests only minimal correlation between single-cell and spatial clusters, an issue that is not sufficiently addressed or discussed. Several figures obtained from this data (3C, 4E, etc) provide little interpretable information beyond very basic mapping of gene expression, mostly restricted to the optic lobes. As a result, many of the conclusions drawn from the spatial transcriptomics data appear confusing or limited in strength. For example, mapping of Sp12 shows a lateral aggregate of putative clock neurons similar to that observed in related species, but the absence of a dorsal population is not addressed, leaving the reader guessing whether this population is missing in An. gambiae or simply not visible in the data. In this situation, I recommend a clear shift in emphasis: the spatial transcriptomics should be presented explicitly as a preliminary, low-resolution mapping resource. In contrast, greater weight should be placed on the in situ hybridization experiments, which appear more accurate and informative for validation and mapping. Accordingly, the authors should revise the manuscript to carefully temper and clarify ambiguous conclusions arising from the spatial transcriptomics data. Analysis of cellular anatomy

would also benefit from clearer signposting of similarities and differences with related species to clarify which features are conserved, or specific to An. gambiae.

Answer to Reviewer 1, comment 2.

We thank the Reviewer for encouraging us to add a more precise description of the approaches we applied, and agree that the manuscript would benefit from this. As tissue slices for both ISH and spatial transcriptomics are generally 10 µm thick, the techniques based on histological sectioning of tissues provide limited information on the 3D anatomy of whole organs, failing to highlight multiple sub-populations located in different areas of the brain, such as the lateral and the dorsal clock neurons, as the Reviewer evidenced. In addition to this, spatial transcriptomics approaches allow to explore a tissue gene expression with just a 10 µm resolution, and more than one cell population could be captured in the same spatial spot. To clarify these limitations, we have added to the main text, in the introductory paragraph of spatial transcriptomics, a statement on how we complemented the high-resolution ISH with a lower resolution spatial transcriptomics (line 161-162), and commented on how many Sp spots possibly captured multiple cell signatures (lines 170 and 570-574), as suggested by the Reviewer. This is now immediately followed by the details on Slide-seq spatial resolution and the number of high-quality spots recovered, providing the reader with all the information needed to understand the relevance of the approaches we used. We also state that the tight packing of the cell body rind in combination with current technical restriction of spatial transcriptomics could lead to multiple cell types being captured in the same cell body rind cluster and render the identification of specific structures difficult, especially those composed of a few neurons in each brain (lines 407-410). We have also discussed how future studies could provide a higher resolution mapping of gene expression in the *A. gambiae* brain (lines 573-574). As suggested by the reviewer, we have now also stated the incomplete overlap of spatial clusters and marker genes for Sp1 and Sp4 and glia (lines 230-231, 240), and identified Sp12 as a “clock neuron-enriched cluster” (line 316, 324-325, 331, 439, 494). Finally, we have included a clearer description of the interspecies anatomical differences (lines 141-154).

3. The response to my concern about mosquitoes being potentially protein deprived is flawed. The fact that protein deprivation in sugar-fed female Anopheles was never reported does not mean that it is not a possibility. Unaffected lifespan also does not rule out a protein deprivation state: lifespan is barely affected in protein-deprived Drosophila, despite them developing strong protein cravings and clear physiological shifts. Therefore, the authors should acknowledge that transcriptional changes attributed to the blood meal may be influenced by prolonged protein deprivation in sugar-fed controls.

Answer to Reviewer 1, comment 3.

We thank the Reviewer for clarifying this observation. To report this possibility, we now have a statement specifying the possibility that a delayed blood meal after mating may be a stressor (lines 655-658).

Additional (minor) comment:

Fig S5: cluster names are still ‘An’, not ‘Ag’.
Answer to Reviewer 1, minor comment.
We thank the reviewer for noticing this typo and have corrected Fig. S5.

Reviewer #2 (Remarks to the Author):
The authors have provided a comprehensive revision that addresses my concerns.
Answer to Reviewer 2
We are glad we managed to answer satisfactorily to the observations of the Reviewer, and would like to renew our gratitude for the valuable comments on the manuscript.

Reviewer #3 (Remarks to the Author):
I thank the authors for their thorough and constructive revision. They have addressed all of my original comments satisfactorily. The introduction now provides the scholarly framing and motivation that were previously missing. The addition of pan-neuronal classification, the documentation of batch-effect handling and merged design, and the new cluster-level single-cell differential expression analysis integrated with the bulk RNA-seq results substantially strengthen the "Life history" section and address my central concern regarding the linkage of transcriptional changes to cell types. The Discussion has been developed into a well-contextualized section, and the data and code have been made publicly available. This revised manuscript represents a valuable resource and a clear improvement over the original submission. I am happy to support publication once a few minor remaining points are addressed.
Answer to Reviewer’s 3 remarks
We are sincerely grateful to the Reviewer for the time spent in assessing this manuscript and his thoughtful comments and we are glad we managed to address all the original questions.

Minor comments
1. Two editing artifacts remain in the figure legends and should be removed. The Figure S4 legend ends with the note "Scale bar 50 µm update legend." The Figure S9 legend contains a duplicated fragment from the Figure 4 caption: "Distribution of vision-related genes… Figure 4. Optic lobe and circadian neurons in the visual system of An. gambiae…"
Answer to Reviewer 3, minor comment 1.
We want to thank the reviewer for the careful revision down to this level of detail. We have corrected the descriptions of Fig. S4 and Fig. S9 accordingly.

2. There is a gene-ID inconsistency for the brp ortholog. Figure 1B refers to AGAP010286, while the Figure 1B legend lists AGAP010296. Please reconcile these identifiers and confirm the correct one is used consistently throughout the manuscript.
Answer to Reviewer 3, minor comment 2.
We thank the Reviewer for the thorough review and have now corrected the typo in the Fig. 1 legend.

3. The replicate numbers for the bulk life-history data appear inconsistent. The Figure 5 legend states n = 39 single-brain samples, whereas the Methods section lists virgin (n = 9), mated (n = 10), and blood-fed (n = 10), totaling 29. Please verify the sample count and reconcile the figure legend with the Methods section.

Answer to Reviewer 3, minor comment 3.

We thank the reviewer for noticing this typo and have corrected it in the legend of Fig. 5.

4. In Table S7, the Ag37 genes described as down-regulated after mating (GPROP3, arrestin-1, GPROP4) have positive log2FC values, indicating upregulation under the table's mated-versus-virgin convention. Additionally, AGAP029557 does not appear in the Ag37 significant list. Please reconcile the direction of regulation described in the text with the supplementary table and confirm the accuracy of the gene list.

Answer to Reviewer 3, minor comment 4.

We thank the reviewer for noticing this inconsistency. We have now updated the text discussing the relevant genes (line 438-452).
