# Supplementary material for: The brain of the female Anopheles gambiae mosquito at spatial and single-cell resolution
Source: Nat Commun. 2026 Sep 17;17:10940. doi: 10.1038/s41467-026-76839-z (PMC13680466; doi:10.1038/s41467-026-76839-z)
Supplement: Supplementary file 4 [file 41467_2026_76839_MOESM3_ESM.pdf]

Description of Additional Supplementary Files

File Name: Supplementary Data 1
Description: Notable cell type marker genes.

File Name: Supplementary Data 2
Description: Cell type annotation and spatial cluster annotation.

File Name: Supplementary Data 3
Description: All marker genes of cell and spatial clusters.

File Name: Supplementary Data 4
Description: Spatially variable genes in eye region.

File Name: Supplementary Data 5
Description: MIP markers, KC markers, Monoaminergic/peptidergic neurons markers.

File Name: Supplementary Data 6
Description: Differentially expressed genes in life history, bulk RNA-seq.

File Name: Supplementary Data 7
Description: Genes differentially expressed after mating, scRNA-seq.

File Name: Supplementary Data 8
Description: Genes differentially expressed 24 h after bloodmeal, scRNA-seq.
